# Characterization of testis-specific serine/threonine kinase 1-like (TSSK1-like) gene and expression patterns in diploid and triploid Pacific abalone (*Haliotis discus hannai*; Gastropoda; Mollusca) males

**DOI:** 10.1371/journal.pone.0226022

**Published:** 2019-12-11

**Authors:** Eun Jeong Kim, So Jeong Kim, Choul Ji Park, Yoon Kwon Nam

**Affiliations:** 1 Department of Marine Bio-Materials and Aquaculture, Pukyong National University, Busan, Republic of Korea; 2 Genetics and Breeding Research Center, National Institute of Fisheries Science, Geoje, Republic of Korea; University of Hyderabad, INDIA

## Abstract

Testis-specific serine/threonine kinase 1-like (TSSK1-like), which plays important roles in late-phase spermatogenesis and male fertility, was characterized in Pacific abalone *Haliotis discus hannai*, an important commercial marine gastropod. Further, its expression patterns were assessed in diploid and induced triploid males showing differential degrees of testis maturation. Abalone TSSK1-like shared conserved structural features with mammalian TSSK1s and other potential metazoan orthologs, especially regarding the catalytic STKc domain. Phylogenetically, abalone TSSK1-like displayed a genetic affiliation with its molluscan TSSK1-like orthologs and human TSSK1. Additionally, abalone TSSK1-like gene showed a tetrapartite exon-intron organization, unlike the intronless structure of most amniotic tetrapodian TSSK1s. Molecular phylogenetic analysis in the metazoan lineage suggested a possible revision in the origin of the earliest ancestral TSSK1. Further, abalone TSSK1-like showed testis-predominant expression, which was significantly influenced by both age and seasonal reproductive cycles. Comparative expression analyses between diploid and triploid abalone males suggested that robust TSSK1-like expression occurred primarily at the post-meiotic stage. Additionally, RT-PCR assay indicates that mature abalone sperms retain TSSK1-like transcripts after release. Taken together, this study provides useful insights for further studies to assess male reproduction and sterility and/or partial fertility of induced male triploidy in abalone species.

## Introduction

Testis-specific serine kinases (TSSKs) are a family of serine/threonine kinases that belong to the 5´-adenosine monophosphate-activated protein kinase (AMPK) family. TSSKs are highly expressed in the testis, and increasing evidence from mammalian studies has strongly suggested important roles of TSSKs in the regulation of many spermatogenesis-related protein activities and male fertility [1–3]. Currently, it is generally agreed that most mammals including human possess five TSSK family members (i.e., TSSK1, TSSK2, TSSK3, TSSK4, and TSSK6), although there have been some confusion for their nomenclatures due to different names given to the same proteins [4,5]. Different TSSK members represent, at least partially different spatial and/or temporal expression patterns during the maturation of male gametes, however, each member-specific function or coordinated role(s) between members should be further elucidated [4,5]. Of these, TSSK1, shows specific expression only during spermiogenesis in sexually mature male mammals, implying its crucial role in the preparation of functional spermatozoon, including the control of morphological differentiation of spermatids into mature spermatozoa [5].

Evolution of TSSK1 in the mammalian lineage has been proposed to be based on the retrogene duplication mechanism. As an intronless gene, TSSK1 (or TSSK2) first appeared in the common ancestor of mammalian species, in which an RNA-based retroposition or retrotransposition event of TSSK1 (or TSSK2) occurred, shortly followed by DNA-based gene duplication forming the TSSK1-TSSK2 tandem arrangement. During the duplication event, TSSK1 (and TSSK2) potentially gained a neofunction associated with testis-specific expression in spermatogenesis [6,7]. In the primate lineage, another gene duplication gave rise to TSSK1B (the newest gene of the TSSK branch) through retroposition of TSSK1. The resultant retrogene copy (TSSK1B) has survived as a functional gene, while the parental TSSK1 became the pseudogene TSSK1A in Simiiformes [7,8].

However, in contrast to mammals, little is known of the evolution of TSSK1 and TSSK1-like members in molluscans. Although TSSK1-like sequences have been identified in genome drafts of several commonly studied molluscan species, most are hypothetical or predicted genes based on computational similarity analysis. Compared to well-conserved structural features among mammalian TSSK1s, relatively high structural diversity is currently predicted among molluscan sequences named TSSK1 (or TSSK1-like). Furthermore, within some molluscan species, different proteins with large variations in size and shape have been co-annotated under the same nomenclature of TSSK1 (or TSSK1-like). At the genomic level, all molluscan sequences annotated as TSSK1 (or TSSK1-like) genes may not display the intronless gene organization, which is discordant with the single exon structure that has been invariantly found in most mammalian TSSK1 genes. More importantly, barring only a few previous reports in bivalves [9,10], few studies have characterized TSSK1 (and TSSK1-like) expression in molluscans, particularly in gastropodian organisms.

Pacific abalone, *Haliotis discus hannai* (Gastropoda; Mollusca), is a commercially important marine gastropod species in Korea and other East Asian countries. During the last decade, aquacultural production of Pacific abalone in Korea has rapidly expanded in parallel to the development of its sea cage farming technique [11]. Currently, Pacific abalone is the top ranked shellfish species by value in Korea, with Korea as one of its major suppliers in the global abalone market [12]. Due to economic interests, genetic breeding studies of Pacific abalone are progressing in Korea, including selective breeding, interspecific crossbreeding, and chromosome-set manipulation [11,13]. Therefore, a comprehensive understanding of genes and proteins associated with sexual maturation for precise reproductive control is a fundamental requirement for all these breeding investigations. In particular, triploidization has been considered as a potential means to prevent unwanted reproduction of farmed abalones [13,14]. Massive and uncontrolled spawning of mature abalones (especially males) in intensive sea cage facilities around spawning season often leads to deterioration of water quality, increased disease susceptibility, and hypoxia-related outbreak of mortality. In addition to reproductive confinement, triploidization may be a potential tool to improve quantitative traits of farmed abalones due to the presumed ability of triploids to channel the energy required for gonad maturation to somatic growth [15]. However, despite its importance, the molecular aspects underlying the reproductive characteristics of induced triploid abalones have been rarely explored.

Accordingly, this study aimed to characterize a novel TSSK1-like gene in the Pacific abalone species *Haliotis discus hannai*. Based on the characterization of genomic gene structure and cloning of the full-length cDNA, the potential orthology of this abalone TSSK1-like to human TSSK1B was examined. Phylogenetic relationships of this gastropodian TSSK1-like gene with its potential orthologs in the metazoan lineage were investigated to assess the origin and evolution of TSSK1. To examine the potential involvement of this TSSK1-like in abalone spermatogenesis, various expression assays with regards to testicular developments in diploid and artificially induced triploid abalone males were performed.

## Materials and methods

### Abalone specimens

Abalone *Haliotis discus hannai* specimens used for gene cloning and tissue distribution assays of TSSK1-like transcripts were from experimental stocks maintained at the Experimental Fish Culture Station, Pukyong National University (PKNU), Busan, Republic of Korea. Specimens used for mRNA expression assays regarding ages of male abalones and reproduction cycles were stocks maintained at the Genetics and Breeding Research Center, National Institute of Fisheries Science, Geoje, Gyeongsangnam-do, Republic of Korea. Diploid and triploid males used to compare TSSK1-like mRNA expression levels were produced in the Genetic Research Center (NIFS), transferred to PKNU facility, and then further maintained until assayed. The described experiments were approved by the Animal Care and Use Committee of Pukyong National University. All experimental procedures were performed in accordance with the National Act on Laboratory Animals.

### Molecular cloning of cDNA and genomic genes

From our local NGS database of *Haliotis discus hannai* gonads, a partial clone showing a significant match to animal TSSK1 and/or TSSK1-like was selected, and rapid amplification of the cDNA end (RACE) was performed at both 5´- and 3´-directions using SMARTer RACE 5´/3´ Kit (Takara Bio Inc., Mountain View, CA, USA) according to the manufacturer’s instruction. Based on the contig assembly, a continuous version of the cDNA sequence containing a potentially full-length open reading frame (ORF) was isolated from the total RNA of the testis of a mature male using reverse transcriptase PCR (RT-PCR). The RT-PCR product was cloned into pGEM-T easy vector (Promega, Madison, WI, USA) and sequenced at both directions (four recombinant clones). Subsequently, cDNA fragment containing an ORF was amplified from each testis of eight unrelated male individuals, and the RT-PCR products were directly sequenced again to examine the presence of any polymorphic sites in the coding sequence among individuals. Information on oligonucleotide primers used in this study is listed in S1 Table.

Based on representative cDNA sequence, multiple primer pairs were designed to isolate genomic fragments of abalone TSSK1-like by PCR, and gaps between genomic fragments were filled by the genome walking method using the Universal Genome Walker Kit 2.0 (Clontech Laboratories Inc., Mountain View, CA, USA), as needed. Genomic sequence spanning from 5´-untranslated region (UTR) to putative polyadenylation signal was confirmed by PCR isolation of five overlapping genomic fragments. Each genomic fragment was sequenced at both directions.

### Classification, molecular phylogeny, and bioinformatics analysis

To verify the classification of current TSSK1-like isolated from this abalone species, we first carried out molecular phylogenetic analysis of abalone TSSK1-like against the following five reference sequences of human TSSK members: TSSK1 (TSSK1B), TSSK2, TSSK3, TSSK4, and TSSK6. Different TSSK (and TSSK-like) members from selected mollusk species were retrieved from GenBank database to be included in the phylogenetic analysis. Additionally, one sequence from *Lottia gigantea* (Gastropoda), which had been annotated as a hypothetical protein in GenBank but displayed significant sequence homology to the present abalone TSSK1-like in a BLASTx search, was included in the analysis. Information on the TSSK sequences used for phylogenetic classification analysis is directly indicated on the phylogenetic tree. Molecular phylogenetic trees were reconstructed by maximum likelihood (ML) and neighbor joining (NJ) methods using MEGA software (ver. 10.0.5; https://www.megasoftware.net/), including testing of tree topology with 1,000 bootstrap replicates.

Based on the result from the classification analysis, the molecular phylogenetic relationship of abalone TSSK1-like with its potential orthologs in the metazoan lineage was reassessed. From the molluscan group, TSSK1 (and TSSK1-like) sequences showing a close phylogenetic affiliation with the abalone TSSK1-like in the classification analysis were selected. From other animal groups, TSSK1 (and TSSK1-like) sequences containing the conserved catalytic domain of serine-threonine kinase (STKc domain) were selected based on the conserved domain (CD)-Search (https://www.ncbi.nlm.nih.gov/Structure/cdd/wrpsb.cgi). Sequence clones exhibiting low structural orthology to human TSSK1 and abalone TSSK1-like (i.e., not well aligned due to truncated regions, large deletion/insertion, and/or significant sequence dissimilarity throughout the polypeptide region) and partial sequences were removed from the alignment and phylogenetic analyses. Subsequently, when more than one TSSK1 (or TSSK1-like) sequences existed within a given single species, the protein sequence showing the highest aligned score to abalone TSSK1-like and human TSSK1 was selected using the ClustalW alignment tool (https://www.genome.jp/tools-bin/clustalw). Based on this selection regime, 82 unique TSSK1 (and TSSK1-like) sequences, including the abalone TSSK1-like, were selected: 24 sequences from mammalians (including human TSSK1B), 8 from saurians (reptiles and birds), 22 from actinopterygians (bony fishes), 1 from chondrichthyan (cartilaginous fish), 16 from arthropods (4 arachnidians, 1 crab, and 11 insects), 5 from molluscans (including abalone TSSK1-like), 1 from ascidian (tunicate), 2 from Echinodermata (starfish and sea urchin), 1 from brachiopod, 1 from nematode, and 1 anthozoan (sea anemone) (S2 Table). Each of the 82 protein sequences was subdivided into tripartite regions (i.e., N-terminal region, central catalytic STKc/TSSK domain region, and C-terminal region) to examine length variations of each subdivided region among taxonomic groups. Theoretical isoelectric point (pI) values and molecular weights of each protein sequence were calculated using the ExPASy pI/Mw tool (https://web.expasy.org/compute_pi/). Further, the number of coding exons predicted in the gene sequence of each TSSK1 (and TSSK1-like) was examined from the NCBI GenBank database. Molecular phylogenetic NJ trees for TSSK1/TSSK1-like proteins were drawn using the entire protein sequence or sequence corresponding to the conserved catalytic domain. For tree reconstruction using MEGA software, the Jones-Taylor-Thornton (JTT) model was used as a substitution model, and gaps were treated as partial deletions with a site coverage cutoff of 50%. Tree topology was tested with 1,000 bootstrap replicates.

For three-dimensional structural modeling of abalone TSSK1-like in comparison to human TSSK1B, template search and model building were carried out using the ExPASy SWISS-MODEL tool (https://swissmodel.expasy.org/). Further, putative ATP-binding sites, substrate binding sites, and activation loop region of these models were compared using the Swiss-PdbViewer (ver. 4.1.0; http://www.expasy.org/spdbv/).

### Tissue distribution analysis of TSSK1-like transcripts in adult abalone tissues

To examine the distribution pattern of TSSK1-like transcripts in adult abalone *H*. *discus hannai* tissues, end point RT-PCR analysis was performed. From mature adult individuals (four females and four males; 3-year-old; average total weight = 79.5 ± 8.8 g), gill, gut, heart, hemocyte, hepatopancreas, muscle (shell muscle), ovary, and testis were obtained as described previously [16]. From each tissue, total RNA was purified, and a cDNA template was prepared (see below). The ribosomal protein L5 (RPL5; GenBank no. ABO26701.1) was chosen as an internal control for the normalization of expression data based on our previous studies [16,17]. RT-PCR amplification was conducted for 30 cycles for TSSK1-like and 26 cycles for RPL5. RT-PCR products were electrophoresed onto 1.5% agarose gels and visualized by ethidium-bromide staining.

### Testicular expression analysis of TSSK1-like transcripts

#### Expression assay with differently aged males

Expression levels of TSSK1-like mRNA were examined in three different age groups (12, 24, and 36 months post-hatching; MPH) of males that had been grown in the same culture facility. Testes were sampled from five mature males belonging to each age class in each group. The entire testis-hepatopancreas was surgically removed from each male, and approximately one-third of each sample was fixed in 4% buffered neutral formalin to conduct a histological assessment of the testicular developmental status. The remaining portion was preserved in RNAlater (Ambion, Thermo Fisher Scientific, Waltham, MA, USA) on ice for 6 h, and a testicular biopsy was performed with fine forceps and surgical scalpel blades under a stereomicroscope. Total RNA was extracted, and an RT-qPCR assay was performed to quantify TSSK1-like mRNA expression levels.

#### Examination of TSSK1-like expression levels during reproductive cycle

TSSK1-like mRNA expression levels were examined during the reproductive cycle. Testis development stages in the reproductive cycle were categorized based on the classification described by Kim et al. [18]. During the 2-year-old to 3-year-old age interval, testis samples were obtained at the ripe/spent stage (RS-I and RS-II: 24 and 36 MPH, respectively), degenerative/inactive stage (DI: 29 MPH), and early active stage (EA: 33 MPH). Due to large variations of maturation degrees among individuals under our culture conditions, it was impossible to obtain samples that could be clearly assigned to degenerative stage and late active stage. Thus, only three intervals were selected as above. From each stage selected, testis biopsies were performed in six males as described above, followed by an RT-qPCR assay of the TSSK1-like transcripts.

#### Comparison of TSSK1-like expression levels between diploid and triploid males

TSSK1-like mRNA expression levels were compared between diploid and triploid males (half-siblings) at 24 MPH. Triploid abalones were induced using cold shock treatment to block the extrusion of the first polar body, and the ploidy status of each individual used in this assay was verified by flow cytometry [13] (S1 Fig). At 24 MPH, testis biopsies from six diploid and twelve triploid (six ‘sterile-like’ and six ‘partially matured’) males were obtained to perform gonad histology and RT-qPCR assays. In addition, diploid and triploid males were subjected to artificially induced spawning (sperm release) to verify functional male fertility (for diploids) and sterility (for triploids). Males (10 each for diploid and triploid) with similar external gonadal appearances to those used in histological and RT-qPCR analyses were selected, and sperm release was induced individually using the conventional method of ultraviolet (UV)-irradiated sea water treatment [19].

### Examination of TSSK1-like transcripts in released sperm

We examined first whether or not artificially released abalone sperm may retain TSSK1-like transcripts by using end-point RT-PCR. Three sperm samples and two testis biopsies were subjected to end-point RT-PCR according to conditions described above for tissue distribution analysis of TSSK1-like transcripts, and RT-PCR products were sequenced to confirm the correct amplification of target fragment. For the end-point RT-PCR assay, the test reaction for assessing any gDNA contamination was included using a primer pair (INT2-1F/1R) that was complementary to intron 2 of abalone TSSK1-like gene (i.e., primers used for PCR typing of intronic length polymorphism above) as well as two negative controls including blank (no template) and without RT [RT (−)] reactions.

Conversely, RT-qPCR analysis was carried out with five testis samples from each of two different half sibling groups (N = 5 per group) and five replicate sperm samples each from three independent sperm release trials (N = 5 per trial). Testis sampling was conducted as described above. Sperms released into seawater were collected with centrifugation at 5,000 rpm for 10 min at 4 °C, washed briefly using ice-cold phosphate buffered saline (PBS, pH 7.4) and re-pelleted with the centrifugation. Sperm pellets were immediately frozen on dry ice and stored at -85 °C until used. RT-qPCR conditions are as follows.

### Nucleic acid preparation, RT-qPCR assay and gonad histology

Total RNA was extracted using the TriPure Reagent (Invitrogen). Subsequently, the RNA sample was purified using an RNeasy Plus Mini kit (Qiagen), including the DNA removal step, according to the manufacturer’s instructions. Quantity and purity of total RNA samples were estimated based on absorbance ratios (260/280 nm and 260/230 nm) using the Nano Drop ND 1000 spectrophotometer (Thermo Fisher Scientific). For analyzing differential expression levels between/among tissues, an aliquot of the total RNA sample (2 μg) from each tissue was reverse transcribed into cDNA in a reaction volume of 20 μL using the Omniscript Reverse Transcription kit (Qiagen), according to manufacturer’s protocol. The synthesized cDNA was diluted 8-fold with sterile distilled water to be used as template for PCR amplification. On the other hand, for comparing expression levels between testis and released sperm, only 0.75 μg of input total RNA was used for reverse transcription (RT) reaction (reaction volume = 20 μL), due to a relatively lower RNA purification yield from sperm samples. RT reaction was carried out using the same kit, however, resultant cDNA was diluted 4-fold.

Two μL of the diluted cDNA template was subjected to quantitative PCR amplification using the LightCycler 480 SYBR Green I Master mix and LightCycler 480 Real-Time PCR System (Roche Applied Science, Penzberg, Germany). An *H*. *discus hannai* RPL5 was also amplified as a normalization control for TSSK1-like expression levels [16,17]. PCR efficiency (*E*) of each primer pair (for TSSK1 and RPL5) was 0.94 or higher (coefficient of determination *R*^2^ > 0.991). Relative expression levels of TSSK1-like mRNAs across tissue samples were normalized against RPL5 expression using the 2^-ΔCT^ method [20]. However, relative expression levels of TSSK-like mRNAs between testis and sperm samples were estimated based on the direct quantification of cDNA using alkaline hydrolysis of RNA and RiboGreen (Invitrogen, Thermo Fisher Scientific)-mediated detection method [21], because of no availability of appropriate housekeeping genes showing invariant expression between testis and sperm samples.

For gonad histology, gonad samples preserved in neutral formalin were re-fixed in Bouin’s solution, dehydrated through a graded ethanol series, and embedded in paraplast (Leica, Germany). Embedded tissues were sectioned at 4–6 μm thickness and stained with Mayer’s hematoxylin-eosin. Stages of male germ cell differentiation, including spermatogonia, primary spermatocyte, secondary spermatocyte, spermatid and spermatozoon were identified according to previous descriptions made on *Haliotis* species [18,22].

### Statistics

Differences in TSSK1-like mRNA expression levels were tested by ANOVA, followed by Duncan’s multiple range test and/or Student’s *t*-test. Differences in body weight, shell length, and hepatopancreas-gonadosomatic indices (i.e., hepatopancreas and gonad mass as a proportion of the body mass; HGSI) between diploid and triploid males were tested using Student’s *t*-test. Differences were considered to be significant when p < 0.05.

## Results

### Characteristics of abalone TSSK1-like cDNA and genomic gene sequences

**Fig 1:**
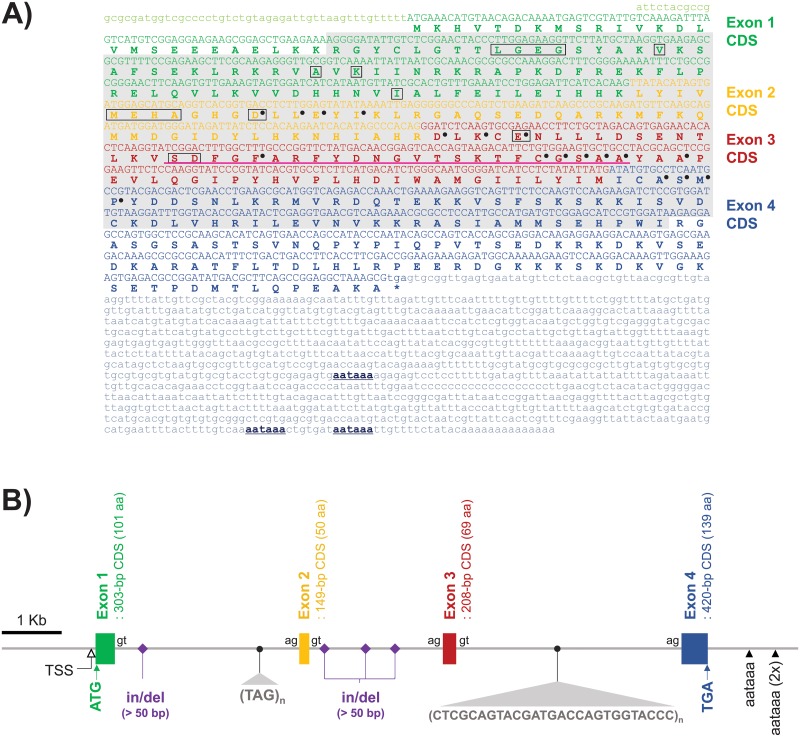
Full-length cDNA sequence and genomic organization of abalone *Haliotis discus hannai* TSSK1-like gene. (A) Coding sequence (CDS) and non-coding sequence of each exon (Exon 1–Exon 4) are indicated by uppercase and lowercase letters, respectively. Deduced amino acid sequence is noted by singlet code below the coding nucleotide sequence. Amino acid residues associated with putative ATP-binding are boxed, while residues for substrate binding are indicated with dots. Putative activation loop is underlined in exon 3. Conserved STKc catalytic domain is shaded grey. Putative polyadenylation signals (AATAAA) in 3´-non-translated region are bolded and underlined. (B) A tetrapartite exon-intron organization of abalone TSSK1-like. Major sites associated with intronic length polymorphisms are noted, including insertion/deletion (in/del) sites (in intron 1 and intron 2), tri-nucleotide (TAG)-repeats (a microsatellite in intron 1), and minisatellite region (in intron 3). Relative positions of transcription start site (TSS) and polyadenylation signals (AATAAA) are indicated. For details on polymorphisms, see S2 Fig.

Full-length cDNA sequence of abalone TSSK1-like was comprised of a 55-bp 5´-untranslated region (UTR), 1080-bp open reading frame (ORF; including TGA stop codon) encoding a polypeptide of 359 amino acids, and 1178-bp 3´-UTR excluding the poly(A+) tail (GenBank no. MG011710.1). In the 3´-UTR, three copies of canonical polyadenylation signal (AATAAA) sequences were predicted at 19, 32, and 463 bp prior to the poly(A+) tail, suggesting possible processing of TSSK1-like transcripts with differential lengths of the 3´-UTR. From the assessment of sequence variation in coding region among individuals (N = 8), five nonsynonymous substitutions were observed in two individuals, while the remaining six individuals shared an identical amino acid sequence, which was further used as a representative sequence of the *H*. *discus hannai* TSSK1-like protein for structure analysis and molecular phylogeny (S2 Fig). Amino acid sequence of abalone TSSK1-like showed conserved features in the STKc domain and for several amino acid residues known to be potentially involved in ATP binding, polypeptides substrate binding, and activation loop formation (Fig 1A).

**Fig 2:**
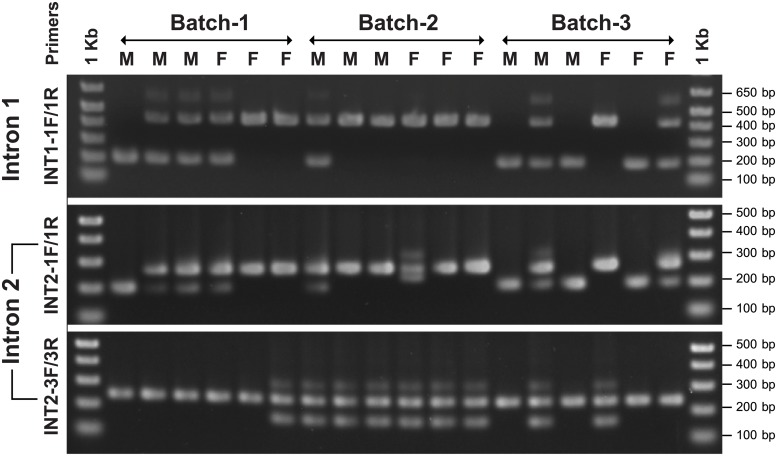
PCR typing of length polymorphisms in introns of abalone *Haliotis discus hannai* TSSK1-like gene associated with large insertion/deletion variation. Mantle genomic DNA of 18 individuals from three unrelated batches [three males (M) and three females (F) per batch] were subjected to PCR testing of intronic length polymorphisms. For the binding site of each PCR primer, sequencing data of polymorphic regions, and amplicon sizes expected from sequencing data, refer to S2 Fig and S1 Table. PCR reaction for the second in/del site in intron 2 failed to successfully generate specific PCR bands in ethidium-bromide stained agarose gel electrophoresis. 1 Kb is the 1 Kb plus DNA ladder (Invitrogen). Original, uncropped raw images supporting all gel results reported in figures of this manuscript are provided as supporting information file S1_raw_images.

Abalone TSSK1-like genomic gene (GenBank no. MN245299) was proven to possess four exons (303 bp, 149 bp, 208 bp, and 420 bp for coding sequences of exon 1–exon 4, respectively) interrupted by three introns (Fig 1B), coding 101, 50, 69, and 139 aa, respectively. Coding sequence of each exon was matched to a representative cDNA sequence, and the consensus GT-AG exon-intron boundary rule was well conserved in each boundary region. In contrast to coding sequences, we found significant intronic length polymorphisms, which were associated with repetitive sequences and insertion/deletion (in/del) polymorphisms in introns. Intron fragments containing presumed polymorphic region(s) were selectively amplified from four individuals (mantle genomic DNA sample each), TA-cloned, and sequenced. As a result, length polymorphisms were observable in every intron. In intron 1, a large in/del region (> 230-bp difference) and a TAG-repeat microsatellite locus were found. Three large in/del length variations differing in 69–259 bp were observed in intron 2, while a minisatellite region comprising differential repeat numbers of a 27-bp consensus unit sequence was detected in intron 3 (S2 Fig). Besides those polymorphisms, several single nucleotide polymorphisms (SNPs) and small in/del mutations were also found in each intron. Based on the sequencing result, selected polymorphic sites were further subjected to PCR typing with 18 individuals chosen from three unrelated batches (three males and three females from each batch) that had been produced with different broodstocks in different years. From the PCR analysis, intronic length polymorphisms tested in this study included both intra- and inter-individual variations. Overall, the sizes of the amplified PCR products were in accordance with those estimated from the sequencing analysis; however, PCR products not matching the sequencing result were also visualized in several individuals (Fig 2 and S2 Fig).

### Phylogenetic classification of abalone TSSK1-like sequence

**Fig 3:**
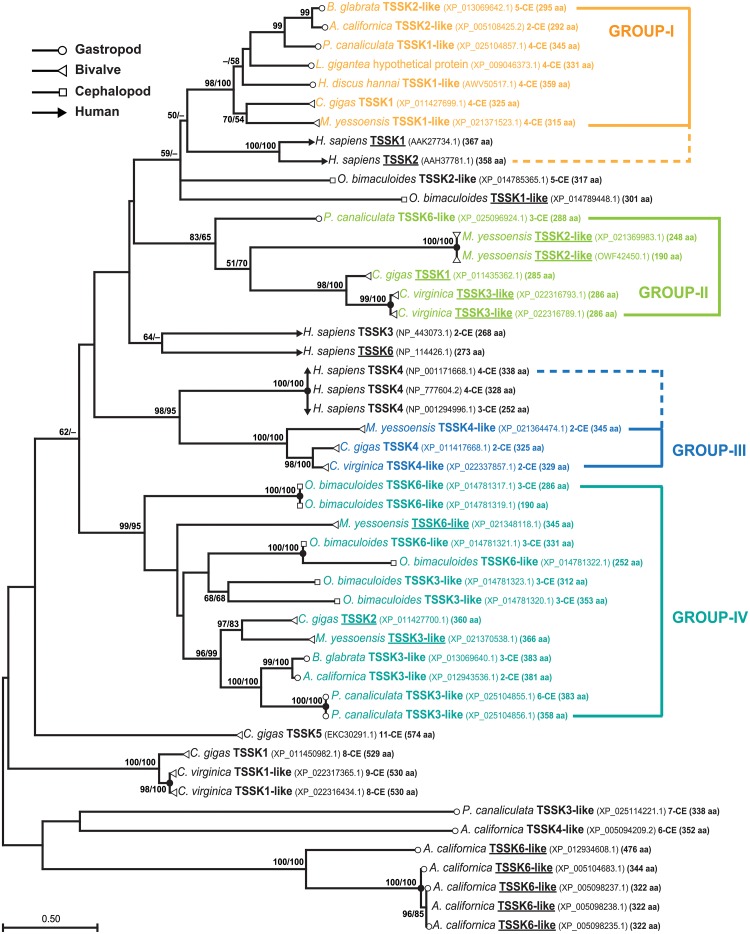
Maximum likelihood (ML) molecular phylogenetic tree constructed using polypeptide sequences of selected molluscan TSSK-like members and human TSSKs as reference sequences. Abalone *Haliotis discus hannai* TSSK1-like protein clustered together with several molluscan TSSK1-/TSSK2-like orthologs and showed a phylogenetic affiliation to human TSSK1 and TSSK2 proteins. Bootstrap scores are estimated based on 1000 replicates, and those equal or higher than 50% are only shown. Bootstrap values in neighbor joining (NJ) tree (S5 Fig) are indicated as ML/NJ. For each taxon, GenBank accession code, number of coding exon (CE), and number of amino acid residues are noted. Intronless gene products are underlined based on the exon prediction in GenBank. Sequence variants and/or alternatively spliced forms of a TSSK member observed within a given species are indicated by closed circle at the node.

**Fig 4:**
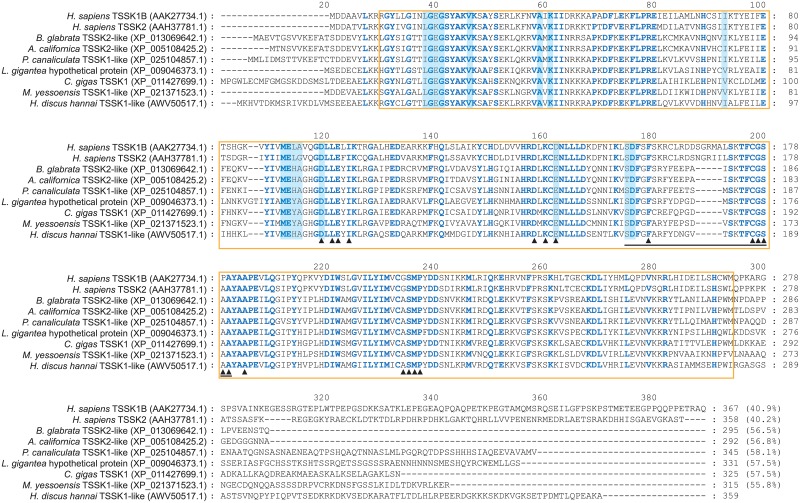
Multiple sequence alignments of abalone *Haliotis discus hannai* TSSK1-like protein against human TSSK1/2 and potential molluscan ortholog (TSSK1/2-like) sequences. Conserved amino acid residues are noted by blue boldface letters. Putative residues associated with ATP binding are shaded *light blue*, while residues for substrate binding are indicated with *arrowheads*. Putative activation loop is underlined and conserved STKc catalytic domain is boxed. Percentage in parenthesis at the end of each sequence indicates sequence identity to abalone TSSK1-like based on calculating the number of identical residues from the length of multiple sequence alignment.

To better classify the abalone TSSK1-like, a molecular phylogenic tree was reconstructed using selected molluscan TSSK-like isoforms and five human TSSKs (TSSK1B, TSSK2, TSSK3, TSSK4, and TSSK6) as reference sequences (Fig 3). As shown in the ML tree, abalone TSSK1-like formed a bootstrap-supported monophyletic clade with other gastropod and bivalve TSSK1/TSSK2-like sequences (labeled Group-I in the ML tree). Further, this molluscan clade was phylogenetically affiliated with human TSSK1B/TSSK2, although the bootstrap support was not strong. Multiple sequence alignments of Group-I sequences with human TSSK1B and TSSK2 demonstrated a considerable number of shared amino acid residues within the conserved STKc domain, but not outside the STKc domain. Within the STKc domain, most residues known to be associated with ATP binding and/or substrate binding were well conserved among sequences aligned (Fig 4). A more direct sequence comparison of abalone TSSK1-like with human TSSK1B and TSSK2 showed that abalone TSSK1-like more closely resembled human TSSK1B than TSSK2 (S3 Fig), and SWISS-MODEL server-based analysis indicated that abalone TSSK1-like and human TSSK1B shared a fairly similar shape in their 3D structural models, regarding overall topologies of α-helices and β-spread sheets, structure of the activation loop, and spatial distribution of binding sites for ATP and polypeptide substrates (S4 Fig).

Besides the Group-I, the second clade contained six molluscan sequences (from 1 gastropod and 3 bivalves; labeled Group-II in the ML tree) that were annotated as TSSK1, TSSK2-, TSSK3-, or TSSK6-like. Except the one gastropodian sequence (TSSK6-like from *P*. *canaliculata*), the remaining five bivalvian TSSK-likes were products of intronless genes. Group-III clade comprising bivalvian TSSK4 (and TSSK4-like) isoforms was closely affiliated with the human TSSK4 sequences. Conversely, Group-IV clade consisted of various molluscan TSSK sequences annotated as TSSK3-like or TSSK6-like (except *C*. *gigas* TSSK2 XP_011427700.1). However, this molluscan group did not show direct phylogenetic affiliation to human TSSK3/TSSK6. In addition to these four Groups, two statistically supported clades formed in a genus or species-specific fashion. The three *Crassostrea* sequences and a monotypic *C*. *gigas* TSSK5 (branched independently of the *Crassostrea* clade) were characterized by long polypeptides (529–574 aa) and a high number of coding exons (8–11 coding exons). Additionally, a species-specific clade consisting of five TSSK6-like sequences from a single species *Astatotilapia calliptera* (gastropod) was formed. Compared to the ML tree, NJ algorithm showed a similar tree topology, in which Groups I–IV formed in the ML tree were reproduced in the NJ tree, although all the subdividing branching patterns were not identical (S5 Fig).

### Structural diversity and genomic organization of metazoan TSSK1/TSSK1-like members

**Fig 5:**
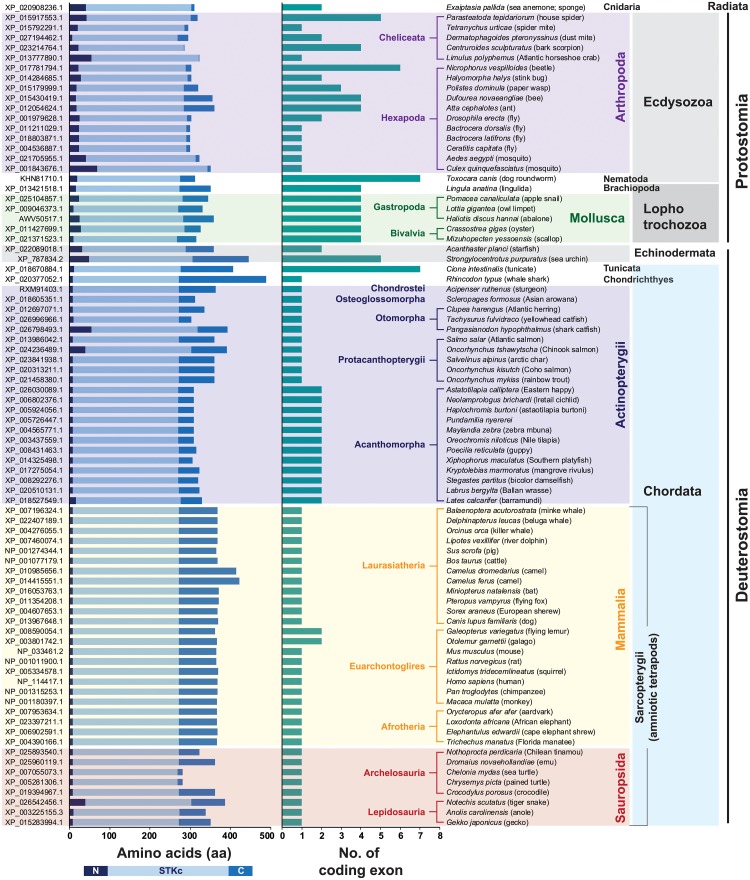
Comparison of primary protein structure and number of coding exons in metazoan TSSK1/TSSK1-like genes.

To determine whether abalone TSSK1-like should be classified as a TSSK1 member, we assessed the molecular phylogenetic relationship of abalone TSSK1-like with its potential orthologs in the metazoan lineage. For this, we first examined structural diversity of metazoan TSSK1/TSSK1-like members selected in this study. Within a set of TSSK1 (and TSSK1-like) protein sequences selected (N = 82), the length of the conserved catalytic domain region was fairly homogenous across all taxa (mean ± SDs = 262.7 ± 3.1 aa; coefficient of variation = 0.01), while lengths of both N-terminal (16.1 ± 13.0 aa; CV = 0.81) and C-terminal (65.8 ± 40.1 aa; CV = 0.61) regions were highly variable depending on the taxonomic groups (Fig 5 and S6 Fig). There was uniformity in length for each tripartite region (N-terminal, catalytic domain, and C-terminal region) in the mammalian group, in which there was no variation in lengths of N-terminal (9 aa) and catalytic (263 aa) domains. For the C-terminal region, lengths of most mammalian TSSK1Bs were 90–99 aa, except two TSSK1Bs from the genus *Camelus* (142 and 149 aa). Length variation for the N-terminal region in other non-mammalian chordates (actinopterygians, chondrichthyan, saurians, and tunicate) was also small (ranging 8–11 aa), excluding a few sequences possessing exceptionally long N-terminal regions in TSSK1-like proteins from *Pangasianodon hypophthalmus* (shark catfish; Siluriformes; 55 aa), *Oncorhynchus tshawytscha* (Chinook salmon; Salmoniformes; 40 aa), *Lates calcarifer* (barramundi; Carangaria incertae; 16 aa), and *Notechis scutatus* (tiger snake; Squamata; 39 aa). However, the lengths of the C-terminal region encompassed a relatively wider range (from 12 aa in turtles belonging to the order Tesudines to 217 aa in a chondrichthyan species). In comparison to chordate TSSK1s (10.9 ± 8.3 aa for N-terminal region), TSSK1/TSSK1-like proteins from non-chordate groups generally possessed longer N-terminal regions (28.3 ± 15.9 aa for Arthropoda; 19.8 ± 9.1 aa for Mollusca; 32 and 48 aa for two echinoderms; 19 aa for one nematode; 16 aa for one brachiopod; 41 aa for one cnidarian). Ecdysozoan (arthropods and a nematode) TSSK-like members had generally shorter C-terminal regions (20.9 ± 22.3 aa) than lophotrochozoans (mollusks and a brachiopod; 61.0 ± 15.3 aa) and chordates (79.6 ± 35.0 aa). Further, theoretical pI values estimated of the entire protein region were the lowest in the mammalian group (pI = 7.15 ± 0.71; CV = 0.10) and the highest in the arthropodian group (pI = 9.15 ± 0.47; CV = 0.05) (S7 Fig). Amino acid sequence identity between the abalone TSSK1-like and its potential orthologs is provided in S2 Table. At amino acid levels, abalone shared the highest sequence identity of TSSK1/TSSK1-like with molluscan members (ranging 69.6 to 71.2%; average 70.6%) and then with a brachiopod species (63.1%). Meanwhile, the average sequence identities with orthologs from arthropodian, actinopterian, mammalian and saurian group were 53.3, 56.6, 58.4 and 59.5%, respectively (S8 Fig).

**Fig 6:**
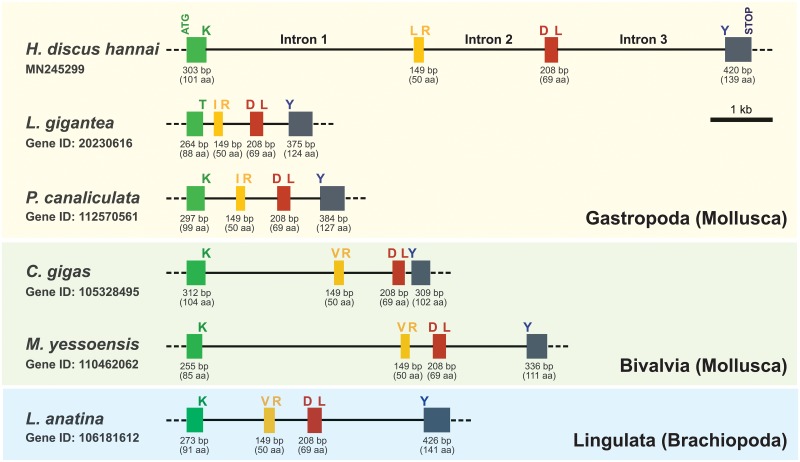
Genomic organization of phylogenetically affiliated lophotrochozoan TSSK1-like genes with four coding exons. Boundary amino acid residues for each exon are noted by singlet code. Conserved positions of exon-intron junctions indicated in multiple sequence alignments of amino acid sequences are also provided in S9 Fig.

At the genomic level, variability in the number of coding exons differed depending on taxonomic groups (Fig 5). With an exception in tunicate (7 coding exons), all chordate TSSK1/TSSK1-like genes were examined to have either 1 (*i*.*e*., intronless) or 2 coding exons. Further, the two-exon TSSK1 genes observed in Sarcopterygii were found only in two mammalian species belonging to the superorder Euarchontoglires, while all other amniotic tetrapods possessed an intronless gene. Additionally, in Actinopterygii, TSSK1-like genes with two exons were found commonly in acanthopterygians (Acanthomorpha), while relatively earlier teleost groups, such as Protacanthopterygii, Otomorpha, and Osteoglossomorpha, and non-teleost fish groups, such as chondrostean and chondrichthyan, represented intronless TSSK1-like genes. Within the protostomic animal group, lophotrochozoans (molluscans and brachiopod) shared a common tetrapartite exon-intron organization pattern for their TSSK1-like genes. These TSSK1-like genes exhibited a conserved pattern of exon-intron partition, in which numbers of amino acids encoded in exons 2 and 3 and boundary amino acid residues between exons (i.e., exon 2/exon 3 and exon 3/exon 4) were clearly conserved, suggesting that they might have evolved from a common ancestral gene (Fig 6). However, ecdysozoans displayed various gene organization patterns, in which variable numbers of coding exons (from intronless to seven exons) were observed.

### Molecular phylogenetic tree of metazoan TSSK1/TSSK1-like sequences

**Fig 7:**
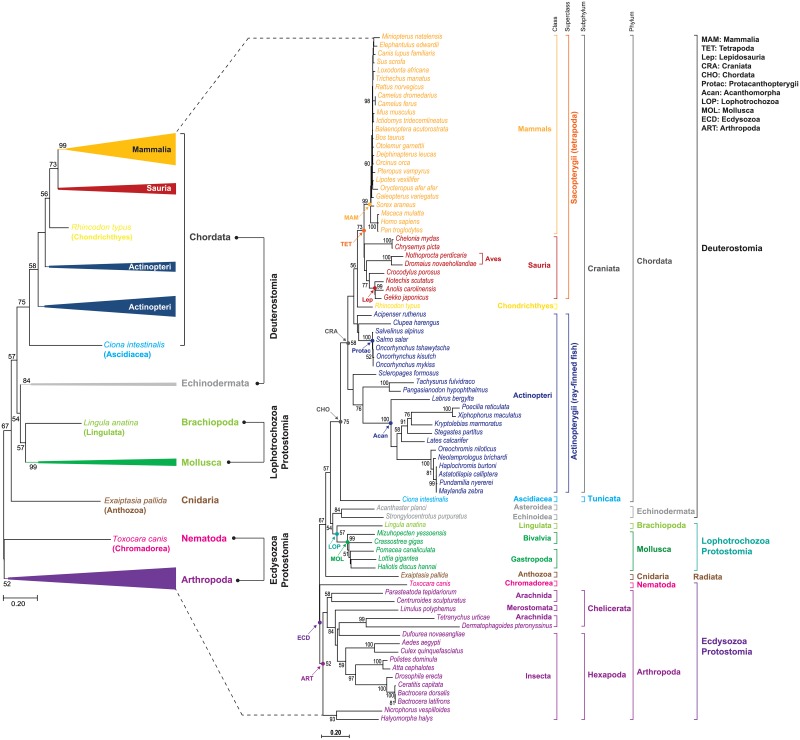
Neighbor-joining (NJ) phylogenetic tree inferred using conserved STKc catalytic domain sequences of TSSK1 (TSSK1-like) proteins in the metazoan lineage. A compressed tree is provided to the left. Bootstrap values (1000 replicates) above 50% are shown in each node. Information on the sequence from each taxon is provided in S2 Table. The phylogenetic tree constructed using the full protein region is provided in S10 Fig.

The phylogenetic tree (82 taxa) was reconstructed using the conserved catalytic domain sequence of each TSSK1/TSSK1-like protein (Fig 7). In the NJ tree, mammalian, tetrapodian, and vertebrate (Craniata) groups were recovered as monophyletic clades. However, within a vertebrate group, actinopterygians formed non-monophyletic clades. Meanwhile, tunicate TSSK-like was placed at the most primitive position of the monophyletic chordate clade in the reconstructed trees. Despite being supported by a weak bootstrap score (less than 50%), the saurian group formed a monophyletic clade in the tetrapodian lineage. For protostomic taxa, monophyletic grouping of molluscan sequences containing the abalone TSSK1-like was consistent, and lophotrochozoan group (*i*.*e*., affiliation of molluscan with brachiopod groups) was also recovered as a monophyletic clade with moderate bootstrap support. Further, despite variable dependence on branching algorithms, arthropodians tended to form monophyletic grouping. Additionally, a monotypic anthozoan (Cnidaria; Radiata) placed at the basal position branched from a large clade containing all deuterostomes and protostomic lophotrochozoans.

From the molecular phylogenetic analysis using the entire protein region of TSSK1/TSSK1-like sequences from the same taxa, phylogenetic relationships according to known appraisal of species were less clearly resolved, in which saurian, ecdysozoan and lophotrochozoan groups were not recovered as monophyletic clades. Moreover, the chordate clade was not monophyletically recovered because the basal status of Tunicata within a phylum Chordata was unresolved in this tree (S10 Fig).

### Tissue distribution pattern of TSSK1-like transcripts

**Fig 8:**
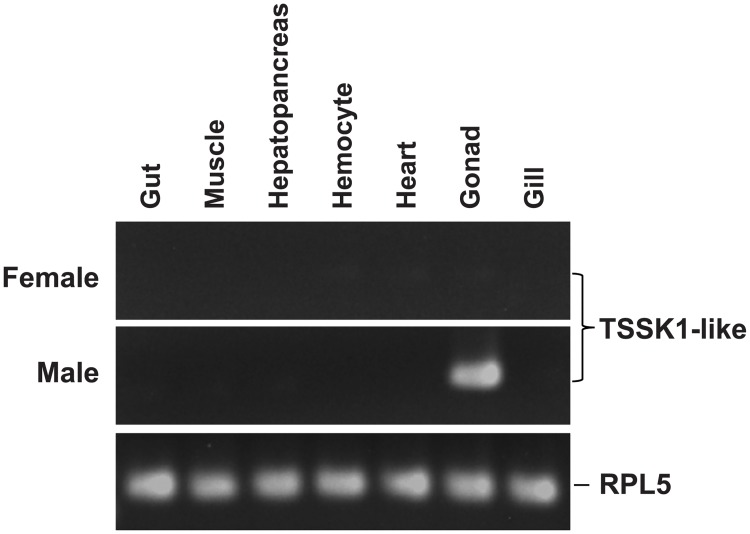
Representative RT-PCR gels showing testis-predominant expression of *Haliotis discus hannai* TSSK1-like transcripts. For internal control (ribosomal protein L5; RPL5) gene, only RT-PCR result with male tissues is shown.

End-point RT-PCR analysis of somatic and gonadic tissues selected from both mature female and male abalones (approximately 3 years old) clearly showed robust abalone TSSK1-like transcripts predominant in the testis under the present assay conditions (Fig 8). Although limited expression could be detected in other tissues, such as hemocyte and heart, depending on individuals under amplification conditions with extended PCR cycle numbers, these expression levels were almost negligible when compared to the testes.

### Age and reproductive cycle-dependent expression levels of TSSK1-like in testes

**Fig 9:**
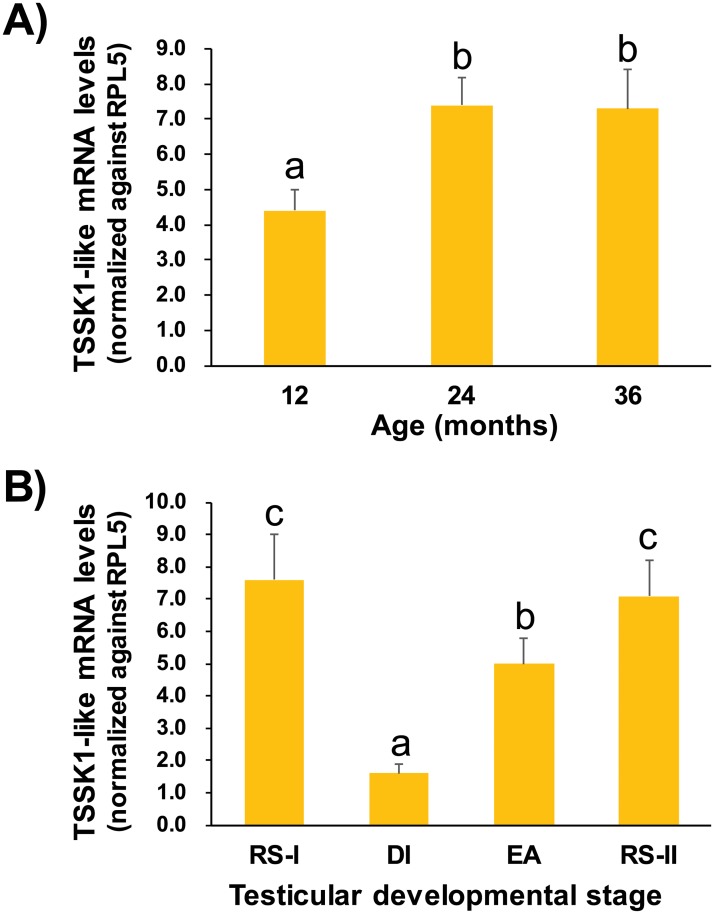
Testicular mRNA expression levels of TSSK1-like transcripts depending on male age and seasonal reproductive cycle. (A) TSSK1-like expression was compared with 1-, 2-, and 3-year-old males (N = 5 for each age group) at spawning season. (B) Expression assays were performed (N = 6 for each stage) at degenerative/inactive stage (DI), early active stage (EA), and ripe/spent stage (RS). The RS-I and RS-II stages correspond to 24 MPH and 36 MPH, respectively. Mean ± SDs with different letters (*a-c*) are significantly different based on ANOVA, followed by Duncan’s multiple range tests at p < 0.05.

RT-qPCR analysis of TSSK1-like transcripts was performed with testes obtained from 1-, 2-, and 3-year-old males at spawning season. In the histological analysis, 1-year-old males already displayed the presence of morphologically differentiated spermatozoa, spermatids, and spermatocytes in their testes. Testes from older males (2- and 3-year-old) also contained spermatocytes and spermatids; however, they were filled with a larger quantity of mature spermatozoa, compared to 1-year-old males (S11 Fig). There was no significant histological difference between 2-year-old and 3-year-old males. Results of RT-qPCR showed that testicular mRNA expression levels of TSSK1-like in relatively older (2- and 3-year-old) males were significantly higher (1.7-fold) than younger males (1-year-old), based on normalization with an internal control RPL5 (p < 0.05). However, there was no significant difference in expression levels between 2- and 3-year-old males (p > 0.05) (Fig 9A).

Testicular levels of TSSK1-like transcripts were affected by reproductive cycle. The robustly high expression level of TSSK1-like transcripts in the testes initially measured at the ripe/spent stage with 2-year-old males (RS-I) was significantly reduced at the degenerative/inactive stage (DI; corresponding to the 29 MPH), which was less than 20% of levels measured at RS-I (p < 0.05). The lowest expression level at DI increased at the early active stage (EA; 33 MPH) (p < 0.05), fully recovering at the next RS stage (RS-II; 36 MPH) (p < 0.05) (Fig 9B).

### Testis development and expression of TSSK1-like transcripts in diploid and triploid males

**Fig 10:**
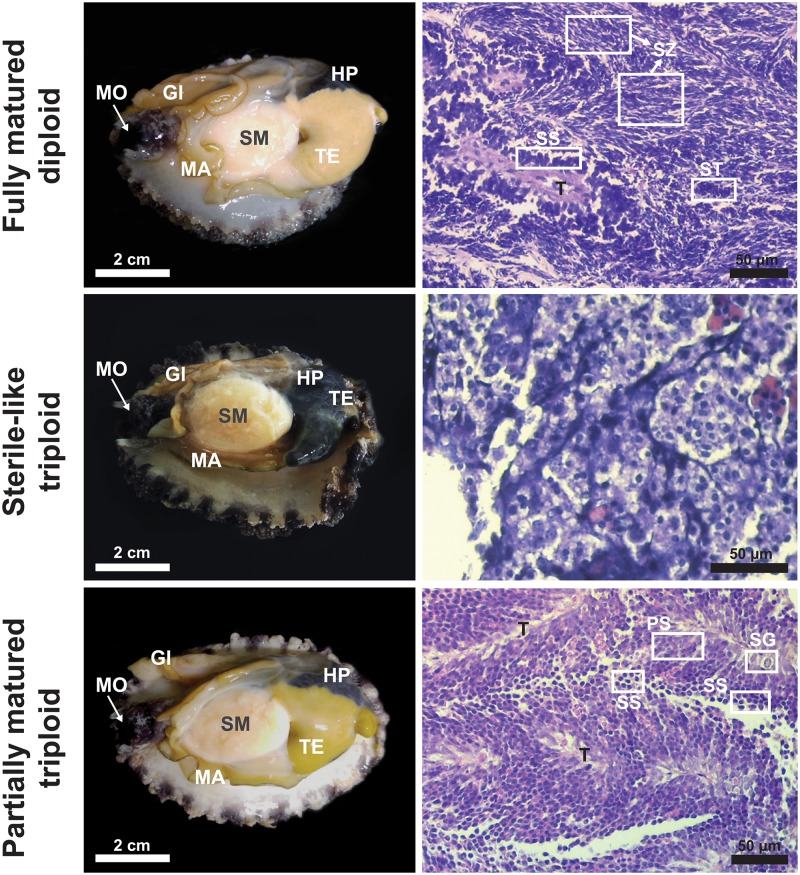
Representative photographs showing testicular appearance and gonad histology of 2-year-old diploid and triploid abalones. Abbreviations in external morphology are mouth (MO), gill (GI), hepatopancreas (HP), shell muscle (SM), mantle (MA), and testis (TE). Abbreviations in histological sections are spermatogonia (SG), primary spermatocyte (PS), secondary spermatocyte (SS), spermatid (ST), spermatozoon (SZ), and trabecula (T). Histological views of diploid and partially matured triploid males at low magnification are also provided in S13 Fig.

In order to select males for analyses of gonad histology and gene expression, we examined a 2-year-old half sibling group comprising 487 diploids and 511 triploids that had been communally grown under the same culture facility. At 24 months, diploid males [N = 237 (48.7%); sex identified easily based on the gonad examination with the naked eye] had well-developed, bright-yellowish colored testes at spawning season, although the extent of maturation was slightly variable among individuals based on visual inspection (Fig 10). Conversely, of same-aged 511 triploids, 219 and 76 individuals could be identified as male and female, respectively, whereas remaining 216 individuals (42.3%) were difficult for clear sexing with unaided eye at this age. Even phenotypically sexed, the 219 triploid males showed a larger variation in the external appearance of testis development among individuals, in which most of them (N = 185) represented poorly developed and remarkably small testis (termed ‘sterile-like’ triploid males in this study). However, about 15% triploid males (N = 34) showed a testicular appearance, which was readily comparable to their diploid counterparts, although size and color of their testes were generally smaller and darker than same-aged diploid males (called ‘partially matured’ triploid males) (Fig 10). HGSI scores (*i*.*e*., relative weight of gonad-hepatopancreas to total body mass, excluding shells) of those partially matured triploid males were significantly lower than those estimated for fully matured, same-aged diploid males (p < 0.05) (S12 Fig). Six individuals each from fully matured diploids, sterile-like triploids and partially matured triploids were chosen on the basis of similar gonadal appearances within a phenotypic group to be used for gonad histology and RT-qPCR assay. In addition, 10 males each from fully matured diploid and partially mature triploid groups were selected induced sperm release trial.

In the histological analysis, gonadal developments of sterile-like triploid individuals corresponded to the early developmental stage without any clear sign of successful meiotic progress. Partially matured triploid males showing considerably developed testis displayed a more progressed pattern of male germ cell differentiation in the histological analysis. Further, the histological analysis detected secondary spermatocytes, which were hardly seen in the testes of almost sterile-like triploids. Nevertheless, spermatids and morphological differentiated spermatozoa were not observed in the gonad histology of partially matured triploid males. Conversely, same-aged diploid males demonstrated a strikingly different pattern of testis development from triploid males. All diploid males examined showed well-developed testes filled with morphologically mature spermatozoa with a small portion of secondary spermatocytes and spermatids (Fig 10 and S13 Fig).

**Fig 11:**
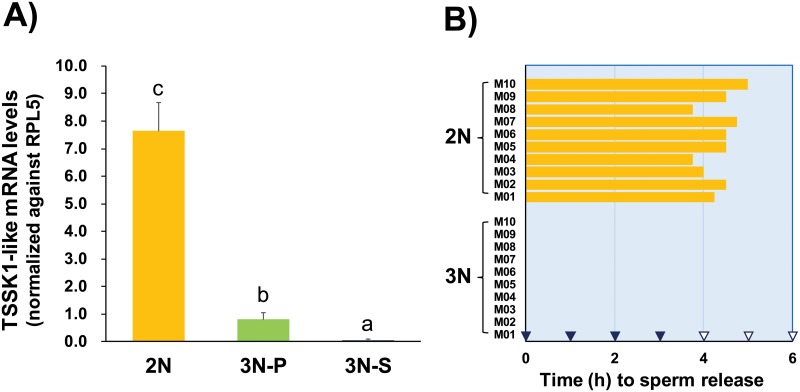
RT-qPCR analysis of testicular TSSK1-like expression and artificial induction of sperm release in 2-year-old diploid and triploid abalone males. In (A), the mean expression levels in diploids (2N; N = 6) were significantly higher than those in the partially matured (3N-P; N = 6) and sterile-like (3N-S; N = 6) triploids based on ANOVA followed by Duncan’s multiple range test (p < 0.05). Statistically different means are indicated by different letters (*a–c*). In (B), only diploid males showed sperm release in response to UV-irradiated seawater treatments. Each time point for UV-irradiated sea water treatment is indicated by *arrowheads* (filled *arrowheads* for both diploids and triploids; open only for triploids). Average body sizes and weights of diploid (N = 10) and triploid (N = 10) males used to induce sperm release, and fertilization rates of released sperm from selected diploid males are provided in S14 Fig.

Sterile-like triploids showed very limited to no expression of TSSK1-like transcripts, based on RT-qPCR results. Comparing TSSK1-like expression between mature diploids and partially mature triploids, testicular TSSK1-like mRNA expression levels were significantly higher in diploids than triploids, based on normalization against expression levels of the internal control RPL5 (p < 0.05). Although increased TSSK1-like expression was found in partially mature triploid males compared to sterile-like triploids, their expression levels were still approximately 10% of those observed in mature testes of diploid males (Fig 11A). In an experimental trial of sperm release, all diploid males (N = 10) began to release sperm within 5 h after the first treatment of UV-irradiated seawater. However, 0 of 10 triploid males (partially matured) induce sperm release under the same treatment conditions or following additional treatments of UV-irradiated seawater for an extended period (Fig 11B).

### Level of TSSK1-like mRNAs in released sperm

For RT-PCR normalization, we first tested expression levels of various housekeeping gene transcripts (as candidate normalization references) in released sperms in comparison with those in mature testes. Candidate genes tested were different ribosomal proteins (RPL3, RPL5, RPL7 and RPL8), cytoskeletal β-actin and glyceraldehyde 3-phosphate dehydrogenase (GAPDH). However, none of genes tested showed a stable expression between testis and sperm samples, in which expression level of each gene in released sperms was markedly lower (several hundred folds) than in mature testes, suggesting that the use of these housekeeping genes for RT-PCR normalization may be unrealistic for precise interpretation of RT-PCR data (data not shown). For this reason, we used direct cDNA quantification method [21], rather than the inclusion of housekeeping gene as a normalization reference in quantitative expression assay.

**Fig 12:**
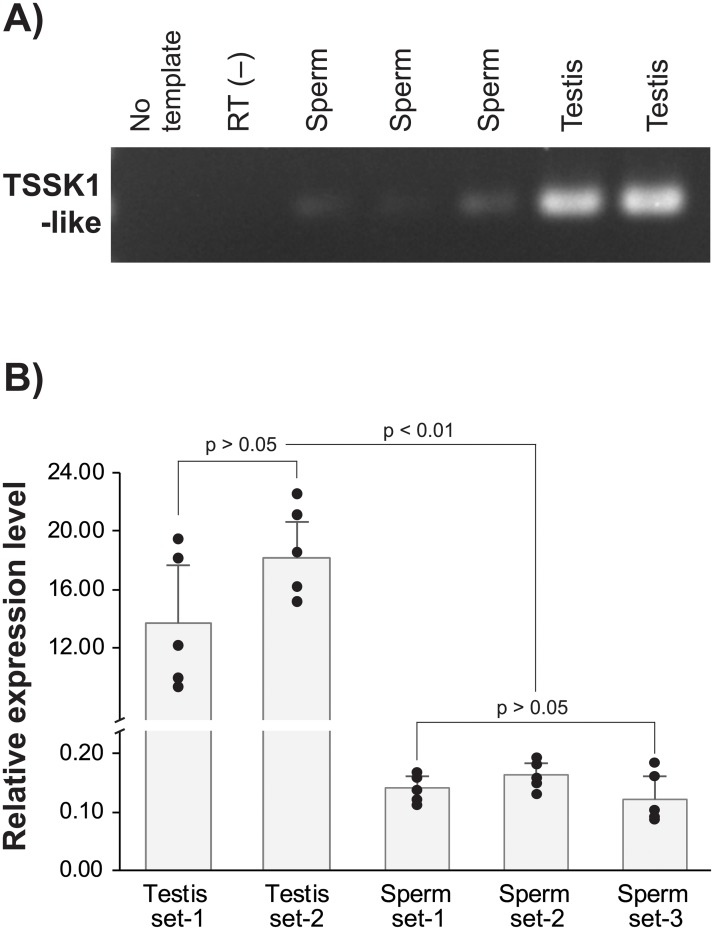
Expression analysis of TSSK1-like mRNAs in released sperms in comparison with those in mature testes. (A) Representative end-point RT PCR gel showing the amplified TSSK1-like bands from three sperm and two testis total RNA samples. Negative blank (no template) and no RT reaction [RT (−)] showed no amplification. (B) Quantitative measurement of TSSK1-like mRNAs using RT-qPCR analysis. Five testes samples were obtained each from two different half sibling male groups (Testis set-1 and Testis set-2), while five replicate sperm samples were collected from three independent sperm release induction trials (Sperm set-1, -2 and -3). Relative expression levels were normalized based on the direct quantification of cDNA [21].

End-point RT-PCR using sperm total RNA yielded the expected size of amplified product, whereas all the negative control reactions consistently showed a PCR negative signal. However, even if TSSK1-like amplified, intensities of RT-PCR bands with sperm samples were significantly weaker than those with testes. Conversely, RT-qPCR assay more highlighted the difference in expression levels of TSSK1-like between the testis and released sperm, in which the average difference between the two sample types (testis vs. sperm) was more than 160-fold. Collectively, abalone sperms retain TSSK1-like mRNAs even after release, however, the amount of mRNAs in the released sperm was significantly lower than that observed in the testes (Fig 12).

## Discussion

The first member of the TSSK family, TSSK1, has received much attention as a key player involved in the post-meiotic process of male germ cell differentiation [5,23]. In this study, we characterized a novel TSSK1-like gene from Pacific abalone *Haliotis discus hannai*, a commercially important marine gastropod, revisited its molecular phylogenetic relationship with other metazoan orthologs, and performed a series of gene expression assays regarding testis development and germ cell differentiation in diploid and triploid abalone models.

The abalone TSSK1-like polypeptide sequence contained a conserved catalytic STKc domain, which is also shared by its potential orthologs, including mammalian TSSK1s. Conserved features are highlighted in amino acid residues that are potentially associated with ATP binding, substrate binding, and activation loop formation in TSSK proteins [4]. Molecular phylogeny-based classification analysis suggests that abalone TSSK1-like cloned in this study has a phylogenetic affiliation to human TSSK1. Comparison of 3D structure between abalone TSSK1-like and human TSSK1 (TSSK1B) has also indicated that high similarity in overall shape and in several regions known to be essential in the structure and function of the TSSK1 protein, which is also broadly congruent with the 3D model predicted for the TSSK1-like protein from a bivalve species [10]. Taken together, cDNA and amino acid sequences suggest structural orthology of abalone TSSK1-like to human TSSK1.

However, at the genomic level, the abalone TSSK1-like gene showed a multi-intron structure (tetrapartite organization), which differs from the intronless structure of most mammalian TSSK1 genes, including human TSSK1B. In mammals, the intronless structure of TSSK1 genes serves as trace evidence of their retroposition (or retrotransposition)-mediated evolutionary origin [7]. Although intronless gene structure is observable in the molluscan TSSK-like gene family members (annotated TSSK1-, TSSK2-, TSSK3-, and/or TSSK6-like genes from different species; see Fig 3), molluscan TSSK1-like members showing close phylogenetic affiliation to human TSSK1 are four-exon genes that exhibit a conserved exon-intron partition pattern. Further, this partitioning pattern is similarly found in the TSSK1-like gene from a brachiopod species, suggesting that lophotrochozoans might share a common ancestor in the evolution of their TSSK1-like genes. Survey of organization patterns of TSSK1 (TSSK1-like) genes from other animal groups also suggests that retrogene-based duplication mechanism for TSSK1 genes may not always be common. Unlike the uniformity of the intronless gene organization in amniotic tetrapods (with exceptions in two Euarchontoglires species), actinopterygians possess either intronless TSSK1-like gene (in relatively earlier evolved fish groups) or bipartite gene (two-exon structure; in recently evolved fish group belonging to Acanthomorpha). Further, in ecdysozoans, more variability is found in the coding exon counts (ranging from intronless to seven exons). Collectively, our data suggest that different taxonomic groups might have undergone their own evolutionary history for TSSK1-like genes.

Additionally, significant polymorphisms were detected in both coding regions (SNPs) and introns (length polymorphisms and SNPs) in the abalone TSSK1-like gene. Increasing evidence has suggested that molluscan species have high genetic diversity and recombination rates, although specific mechanism(s) underlying the diversification and expansion should be further elucidated [24,25]. Genome-wide analysis using a model molluscan species *C*. *gigas* has also highlighted the suggestion of purifying selection as an important driving force in shaping genetic diversity [26]. For SNPs of human TSSK genes, certain SNPs in TSSK2 and TSSK4 genes have been reported to be closely associated with human spermatogenesis impairment and male infertility [27,28], suggesting possible further study to ascertain any potential relationship between SNP genotypes (nonsynonymous substitutions) and phenotypic consequences in the abalone species. Intronic length polymorphisms and sequence diversity associated with in/del and repeat regions have been observed in various genes of molluscan species (mostly bivalves), which have been considered as useful genetic markers for population studies and individual identification [29–32] as well as candidate sites for association analysis of specific physiology [33,34]. Undoubtedly, diverse polymorphisms detected in the abalone TSSK1-like gene could be useful resources to design additional population and functional studies.

In the phylogenetic tree reconstructed using conserved catalytic domain sequences of metazoan TSSK1 (and TSSK1-like), a large clade was recovered as a monophyletic group comprising all deuterostomes, protostomic lophotrochozoans, and a monotypic anthozoan species, although protostomic ecdysozoans branched separately from this clade. The large clade was further subdivided into subclades, of which branching topologies generally agreed with the previous taxonomic appraisal of metazoan members [35]. Although the evolutionary origin of TSSK1-like genes is still difficult to clearly elucidate using the present molecular phylogenetic tree, our phylogenetic data may propose that the evolutionary origin of TSSK1/TSSK1-like genes should be dated further back than previously suggested. In the previous study, the origin of the ancestral TSSK branch of protein kinases had been suggested to be found in ancient amniotes (between 380 and 316 MYA) after diversification between amphibians and amniotes, and TSSK1 first appeared as an intronless gene in the common ancestor of all mammalian species (between 316 and 166 MYA) [7]. However, in our phylogenetic tree, the intronless TSSK1-like genes found in a chondrichthyan taxon displayed a genetic affiliation to the amniotic tetrapodian clade, which may imply that emergence of the intronless prototypic TSSK1 gene might possibly date back to no later than the Osteichthyes-Chondrichthyes split (earlier than 400 MYA) [36,37]. Furthermore, if molluscan TSSK1-like genes (multi-intron) were accepted as true orthologs to mammalian TSSK1s (intronless), the earliest ancestral TSSK origin should be dated back to no later than the protostome-deuterostome split (earlier than 500 MYA) [38,39], although the orthologue relationship between molluscan and mammalian TSSK1s should be further validated. Therefore, the transcribed product (or its partial segment) of the earliest common ancestor of TSSK-like genes with multi-introns might be copied as an intronless retrogene to an ancestral chordate genome(s) after divergence between Tunicata and Craniata. Because most extant vertebrate descendants, except acanthopterygians (the recently emerged fish group containing the TSSK1-like two-exon structure) possess intronless TSSK1 or TSSK1-like gene, retrogene copy (intronless) might have subsequently replaced the parental template gene (multi-intron) in the vertebrate lineage. Further, neofunctionalization of the newly formed retrogene (i.e., testis-specific expression) might have drove negative selection, leading to pseudogenization of the parental gene [40,41]. Conversely, in protostomic and non-chordate groups, the retroposition event from the earliest ancestral gene (multi-intron) might have occurred in a lineage or species-specific fashion, because not all protostomic and non-chordate taxa have been reported to possess intronless TSSK1 gene. Yet, even if they have occurred, retrogenes might not have completely replaced the original multi-intron gene in those genomes, since several molluscans display coexistence of both multi-intron gene and intronless gene that are together annotated as TSSK1-like (and/or TSSK2-like), including *C*. *gigas* and *M*. *yessoensis* (see Fig 3). The coexistence pattern of both the intronless and multi-intron TSSK1-like genes within a given species could also be similarly found in several arthropodians (protostomic Ecdysozoa) from BLAST searches, using the arthropodian TSSK1-like sequences included in our phylogenetic analysis as queries (see also S10 Fig). Taken together, we suggest that the evolutionary history of metazoan TSSK1 (and TSSK1-like) genes should be carefully revised, especially with regards to dating of the emergence of the ancestral TSSK1 (TSSK1-like) origin as well as lineage- or species-specific duplication and divergence patterns of TSSK1 (TSSK1-like) genes in protostomic and/or non-chordate groups.

In the RT-PCR-based tissue expression assay, abalone TSSK1-like displayed a testis-predominant pattern of mRNA expression, which is typically observed in mammalian TSSK1 [4,42]. Furthermore, expression levels of abalone TSSK1-like transcripts were readily affected by both age and reproductive cycles of the males, in which there was a positive relationship between TSSK1-like expression levels and the degree of testis maturation. In this study, histological data were broadly in agreement with previously described male gametogenesis and age of broodstock abalones routinely used for seedling production [19,43]. Significantly higher expression of TSSK1-like transcripts in older males than in 1-year-old males allows, at least indirectly, a possible assumption of TSSK1-like involvement in male germ cell differentiation during late-phase spermatogenesis. However, additional studies monitoring for TSSK1-like expression in younger males may be required to confirm our hypothesis and verify the developmental stage showing the onset of TSSK1-like expression during spermatogenesis.

Nevertheless, the potential involvement of abalone TSSK1-like in the final preparation of mature spermatozoa could be assumed from temporal (seasonal) change of its expression pattern, according to the male reproductive cycle [18]. Although variations were found among male individuals, TSSK1-like expression levels increased during maturation. This finding supports our explanation that the abalone TSSK1-like gene plays a role in the late or final phase of male germ cell differentiation, which is essentially congruent with largely accumulated evidence with mammalian TSSK1s [4,5]. Data from this study are also well in agreement with previous observation of the TSSK1-like homolog in *Atrina pectinata* (pen shell) [10] and the TSSK2-like homolog in *Agropecten purpuratus* (scallop) [44]. However, there is a discordance between present findings and previous observation of TSSK1-like (partial suppression subtraction hybridization clone) in *Mytilus edulis* (mussel), in which the mussel TSSK1-like homolog was upregulated in early developing testis relative to mature testis [9]. Such discrepancies may come from the possibility that molluscan TSSK1-like mRNA expression according to maturation stages could be species-specific.

In triploidy (3N), an organism possesses an extra haploid chromosome set (N) in addition to the normal diploid (2N). Due to abnormal homologous chromosome pairing during meiosis, triploid organisms often display retarded or depressed gonadal development compared to diploids, and thus are commonly experience sterility or infertility [45]. However, unlike triploids induced in vertebrates, triploids induced in aquatic invertebrates often show plasticity in the degree of their sterility, in which some triploids display sexual maturation that is comparable to normal diploids, although their reproductive capability is not equal to diploids (called ‘partially fertile or partially matured’ triploid) [46,47].

In this study, Pacific abalone *H*. *discus hannai* also displayed apparent variability in gonad development among triploid individuals at 24 MPH. Some triploid males showed similar external morphology and maturation of the developed testis compared to same-aged sexually mature diploid males. This variability in triploid males could provide a useful model to study stage-dependent expression characteristics of the abalone TSSK1-like gene in gonad development. From the histological analysis, same-aged and similar-sized triploid and diploid males grown under communal culture conditions represented different stages of germ cell (or gamete) differentiation: (1) retention mostly at pre-meiotic division phase in sterile-like triploids, (2) early meiotic division phase in partially fertile triploids, and (3) post-meiotic differentiation phase in mature diploids.

Testes of sterile-like triploid males did not show successful meiotic division to form secondary spermatocytes, and testicular TSSK1-like mRNA expression was limited or negligible when compared to mature diploid testes, suggesting that TSSK1-like may not play a significant role in mitotic division of spermatogonia or the formation of primary spermatocytes. Impaired meiotic division in the triploid genotype due to the odd number of homologous chromosomes could be a main reason for their sterile-like phenotype, which is widely suggested in other triploid aquatic organisms [45]. Further, transcriptomic analysis in triploid oysters (*C*. *gigas*) has proposed an association between the sterile-like phenotype in triploids and disruption or dysregulation of gene expression related to control of gametogenesis at the early gonad developmental stage [47]. Conversely, partially fertile triploid males displayed testes containing a number of secondary spermatocytes, yet spermatids were hardly detected, suggesting successful progress of the first meiosis, but not yet the second meiosis, until this age (2 years old). TSSK1-like expression level in partially fertile triploid males were elevated compared to sterile-like triploids, but were still very low compared to diploid male testes, which were filled with spermatids (second meiosis progressed) and morphologically matured spermatozoa (spermiogenesis completed). Absence of mature spermatozoa in the testes of 2-year-old partially fertile triploid males was validated by an induced sperm-release experiment, which further supports our data on TSSK1-like expression and gonad histology. Collectively, our expression data suggest that the active TSSK1-like expression occurs mainly at the post-meiotic stage and that abalone TSSK1-like has presumed roles in preparation of metamorphosis and maturation of haploid male germ cells, which are essentially accordant with functions reported in mammalian TSSK1s [4,5]. Nevertheless, even if low, testis without spermatid development (i.e., in partially fertile triploid males) could exhibit TSSK1-like transcription, which is discordant with the expression pattern of mammalian TSSK1s that is restricted only to the post-meiotic stage [5]. Currently, it has yet to be understood whether a certain type of secondary spermatocytes (probably reaching the late phase of the second meiotic division) could express TSSK1-like in this abalone species, or whether this phenomenon could be specific to triploid males, possibly representing a prolonged retention of secondary spermatocytes. Therefore, further study is needed to verify TSSK1-like expression based on the detailed sorting of cell types in the diploid and triploid testes. Additionally, further examination of TSSK1-like gene expression patterns in parallel with testis maturation in older triploid males should be continued to investigate the stage-dependent functional roles of abalone TSSK1-like and to better understand mechanisms regulating spermatogenesis in triploid abalones [48].

Based on both end-point and real-tine RT-PCR assays, abalone sperms would retain TSSK1-like mRNAs even after release, but the retained amount was very lower when compared to that observed in mature testes. Particularly, from RT-qPCR assays, the raw quantification cycle (Cq) values measured in sperm samples were higher in 7–8 cycles than those in testes, which corresponded to about 160-fold lower level of relative expression in average. Transition of haploid spermatid to mature sperm capable of movement requires a lengthy duration and entails a series of physiological changes including the loss of cytoplasm and condensation of chromosomes, in which transcription is believed to be gradually silenced with the compaction of chromosomal structure [49]. Due to this general belief, RNAs retained in released or ejaculated sperm are hypothesized to exist as relics of spermatogenesis. Namely, before terminating of nuclear transcription, various mRNAs needed are transcribed in advance, retained for a certain period of time, and translated into proteins to ensure that all functions subsequent to nuclear transcription are continuing [50]. Within this context, the expression of TSSK1-like transcripts in abalone testes may occur mainly until the late phase of spermiogenesis, and then proteins translated from those transcripts subsequently play physiological roles in mature sperm. Although it is currently unclear why released sperms still retain TSSK1-like transcripts, they might be explained as remnants taking into account only the minute level in released sperm relative to in testes. Thus, functional involvement TSSK1 in released abalone sperm might occur at the protein level rather than at the transcription level, similar to previous reports in mammals [5]. However, to solidify this in abalone, subcellular localization pattern of TSSK1-like with both testes and sperms should be clarified with the development of abalone TSSK1-specific antibody in future study. Additional efforts to discover other TSSK members should be pursued to extend our knowledge on specific and/or coordinated roles between TSSK members in the reproduction and fertility of diploid and triploid males of this abalone species.

In summary, TSSK1-like identified in Pacific abalone *Haliotis discus hannai* exhibits conserved structural features shared by mammalian TSSK1s and other potential orthologs from other metazoan members, especially with respect to the catalytic STKc domain and some important amino acid residues known to be essential for the structure and function of TSSK proteins. Phylogenetically, abalone TSSK1-like has a genetic affiliation with its molluscan orthologs and human TSSK1. Further, abalone TSSK1-like gene has a tetrapartite exon-intron architecture, unlike the intronless structure of most amniotic tetrapodian TSSK1s. Molecular phylogeny with metazoan TSSK1 (and TSSK1-like) orthologs may propose possible revision of the phylogenetic relationship between the origin of the earliest ancestral TSSK1 and lineage-dependent patterns of gene duplication and divergence. From the expression study, abalone TSSK1-like showed testis-predominant expression, which was significantly influenced by the age of males and seasonal reproductive cycles. Comparisons between diploid and triploid males showing differential degrees of testis maturation suggested that TSSK1-like gene expression occurred primarily at the post-meiotic stage. Additionally, TSSK1-like mRNAs could be detectable in released sperm, however, the quantity was much lower than that observed in mature testes. Therefore, our findings could provide a good basis for further studies to better understand or control male reproduction of this commercially important marine gastropod and to gain a deeper insight into the sterility and/or partial fertility of induced male triploidy in this and other related abalone species.

## Supporting information

S1 raw images Original, uncropped raw images of ethidium bromide-stained gels supporting all gel results reported in figures of this manuscript. (PDF)

S1 Fig Representative histograms showing average cellular DNA contents of diploid (upper) and triploid (lower) *Haliotis discus hannai*, based on flow cytometric analysis. Flow cytometry was performed using a CyFlow Ploidy Analyzer (Sysmex, UK) in propidium iodide (PI)-stained mantle cells.
(PDF)

S2 Fig Potential polymorphisms detected in Pacific abalone *Haliotis discus hannai* TSSK1-like gene. (A) Non-synonymous single nucleotide polymorphisms (SNPs) observed in coding region. (B) Major length polymorphisms in intron 1. A large insertion/deletion and TAG-microsatellite locus are notable in intron 1. Within intron 1, base pair numbering is made in accordance with the reference sequence from the abalone individual #1. PCR primers (INT1-1F/1R) to detect individual variations for length polymorphisms are indicated in the above diagram. (C) Length polymorphisms detected in intron 2. Relative positions of three main loci associated with insertion/deletion (in/del) in intron 2 are indicated in the diagram. Multiple sequence alignments of those polymorphic loci. (D) A minisatellite detected in intron 3 of abalone *Haliotis discus hannai* TSSK1-like gene. A 27-bp unit sequence repeating different numbers among individuals (as shown, 62 times). The consensus sequence (CTCGCAGTACGATGCCAGTGKTACCC) of the 27-bp repetitive unit is drawn using Web Logo (http://weblogo.berkeley.edu/logo.cgi).
(PDF)

S3 Fig Sequence comparison between *Haliotis discus hannai* TSSK1-like and human TSSK1/TSSK2 proteins. Of 385 aligned positions, 134 residues are shared by all three sequences.
(PDF)

S4 Fig Three-dimensional structural modeling of abalone TSSK1-like in comparison to human TSSK1B. Models were built using the template (2hak.6) in ExPASy SWISS-MODEL (https://swissmodel.expasy.org/). Overall shape and topology, in particular α-helix and β-spread sheet structures (A), and some essential regions such as putative ATP binding sites, substrate binding sites, and the activation loop region (B) of abalone TSSK1-like were compared to human TSSK1B using the Swiss-PdbViewer (ver. 4.1.0; http://www.expasy.org/spdbv/).
(PDF)

S5 Fig Neighbor-joining (NJ) molecular phylogenetic tree constructed using polypeptide sequences of molluscan TSSK-like members and human TSSKs as reference sequences. Bootstrap scores are estimated based on 1000 replicates.
(PDF)

S6 Fig Multiple sequence alignments of conserved STKc domain sequences from metazoan TSSK1 (TSSK1-like) proteins. In total, 82 taxa were sampled to examine structural diversity and molecular phylogenetic analyses in the metazoan lineage.
(PDF)

S7 Fig Comparison of molecular weight and theoretical pI value of *Haliotis discus hannai* TSSK1-like protein against its potential metazoan orthologs. Molecular weights and pI values are estimated using the ExPASy ProtParam tool (https://web.expasy.org/protparam/).
(PDF)

S8 Fig Mean amino acid sequence identities between abalone TSSK1-like and its potential orthologs from representative animal groups. Sequence identity was calculated with an equation [Identity (%) = (number of identical residues/length of multiple sequence alignment) × 100] using a web tool (SIAS; http://imed.med.ucm.es/Tools/sias.html). T-bar on each histogram is the standard deviation. In the molluscan histogram, the identity with a brachiopod sequence is also indicated with open circle. Sequence identity of each ortholog to abalone TSSK1-like is also provided in S2 Table.
(PDF)

S9 Fig Conserved positions of exon-intron junctions for phylogenetically affiliated lophotrochozoan TSSK1-like genes with four coding exons. In multiple sequence alignments, conserved amino acid residues are indicated by *asterisks*, and the putative STKc domain are shaded *grey*.
(PDF)

S10 Fig Neighbor-joining (NJ) tree showing phylogenetic relationships inferred using complete protein region of TSSK1s (TSSK1-like) sequences in the metazoan lineage. Information on the sequence from each taxon is referred to in S2 Table.
(PDF)

S11 Fig Representative microphotographs showing development status of testes from 1-year-old (1-yr) and 2-year-old (2-yr) *Haliotis discus hannai* males. Histological samples were embedded in paraplast, sectioned at 6 μm thickness, and stained with Mayer’s hematoxylin-eosin. Abbreviations are primary spermatocyte (PS; indicated by *white arrows*), secondary spermatocyte (SS; *yellow arrows*), spermatid (ST; *red arrows*), and morphologically differentiated spermatozoon (SZ; *blue arrows*). Under our culture conditions, full maturation of male abalone is usually obtained from 2 years old. The year classes (1-year-old and 2-year-old) presented here were produced in same month (June) of another year and sampled on the same day during spawning season.
(PDF)

S12 Fig Testis development of 2-year-old mature diploid and partially mature triploid abalone males. (A) Representative photograph showing external appearance of partially mature triploid testis, as compared to fully matured diploid testis. (B) Hepatopancreas-gonadosomatic index (HGSI) scores of triploid males (N = 18), for which testicular appearances were similar to that shown in (A), were compared with scores of mature diploid males (N = 18) in (B). Statistical difference in HGSI score between diploids and triploids was found based on Student’s *t*-test.
(PDF)

S13 Fig Histological sections of testes from 2-year-old diploid (2N) and partially matured triploid (3N) abalone males. Abbreviations are spermatogonia (SG), primary spermatocyte (PS), secondary spermatocyte (SS), spermatid (ST), spermatozoon (SZ), and trabecula (T).
(PDF)

S14 Fig Induced sperm release in 2-year-old diploid and triploid *Haliotis discus hannai* males. (A) Body size and weights of diploid and triploid males subjected to induced sperm release. No significant difference was found between diploid and triploid males (N = 10 each). (B) Representative photographs showing external appearance of testis development in diploid and triploid males. Sperm release in response to UV-irradiated sea water treatment was typically observed with diploid males, but not triploid males. (C) Fertilization rates (%) of spermatozoa released from diploid males shown as the percentage of embryos commencing successful early cleavages (i.e., 2-cell to 4-cell stages) at 2–3 h post-insemination. Mean ± SDs were calculated based on triplicate estimations using at least 110 embryos for each male.
(PDF)

S1 Table Oligonucleotide primers used in this study. (PDF)

S2 Table Information on TSSK1 (TSSK1-like) sequences used in the molecular phylogenetic analyses in the metazoan lineage. (PDF)
