# Supplementary material for: Characterization of testis-specific serine/threonine kinase 1-like (TSSK1-like) gene and expression patterns in diploid and triploid Pacific abalone (Haliotis discus hannai; Gastropoda; Mollusca) males
Source: PLoS One. 2019 Dec 11;14(12):e0226022. doi: 10.1371/journal.pone.0226022 (PMC6905558; doi:10.1371/journal.pone.0226022)
Supplement: S1 raw images — (PDF) [file pone.0226022.s001.pdf]

Information on Raw Gel Images

S1_Raw_Images. Raw images of gels used in Figures (Fig 2, Fig 8 and Fig 12). In this manuscript, Fig 2, Fig 8 and Fig 12 contained 3, 3 and 1 electrophoresed agarose gel images, respectively.

Samples were electrophoresed onto 1.5% or 2% agarose gels at 100 V. The molecular weight size marker for each gel was 1 Kb Plus Ladder (Invitrogen; 100, 200, 300, 400, 500, 650, 850, 1,000, 1650 bp, and 2 to 12 kb, from the bottom). DNA loading dye is a conventional dye containing bromophenol blue (0.25%), xylene cyanol FF (0.25%) and glycerol (30%). For each gel, sample loading to well was made in order from left to right lanes. After electrophoresis, gel was stained with 1 μg/ml ethidium bromide (Sigma-Aldrich) for 10 min and washed with distilled water for 15 min.

Gel image was captured with 'Image Lab Software^{TM}' implemented in Gel doc XR+ imager (Bio-Rad) under default setting conditions. Any adjustment of brightness, color, contrast and others was not made, hence gel photographs in S1_Raw_Images were truly raw gel images automatically captured by the Gel doc software. Each gel image captured with the software was exported to TIF file (1200 pixel). The exported TIF file was opened with Adobe Photoshop CS6 and converted to Photoshop PDF file using 'Save As' function. Resultant seven pdf files (three, three and one for Fig 2, Fig 8 and Fig 12, respectively) were combined into a single pdf file using Adobe Acrobat Pro software. With the 'Comment Tool' of the Acrobat software, the identity of each gel was annotated and lanes not used for figure were also noticed as X.

From these raw image files (TIF files), background adjustment was minimally applied to the entire gel area in order to display similar background brightness/darkness across gels. No adjustment on the specific region was made and no scientific information was changed. Then, band regions from each gel was cropped to be used in Figures.

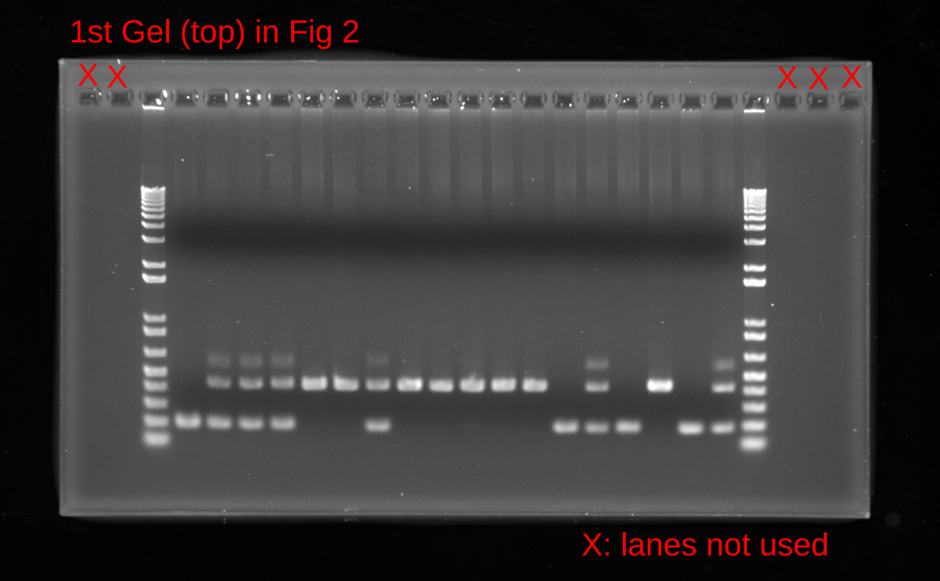
1st Gel (top) in Fig 2
X X
X X X
X: lanes not used

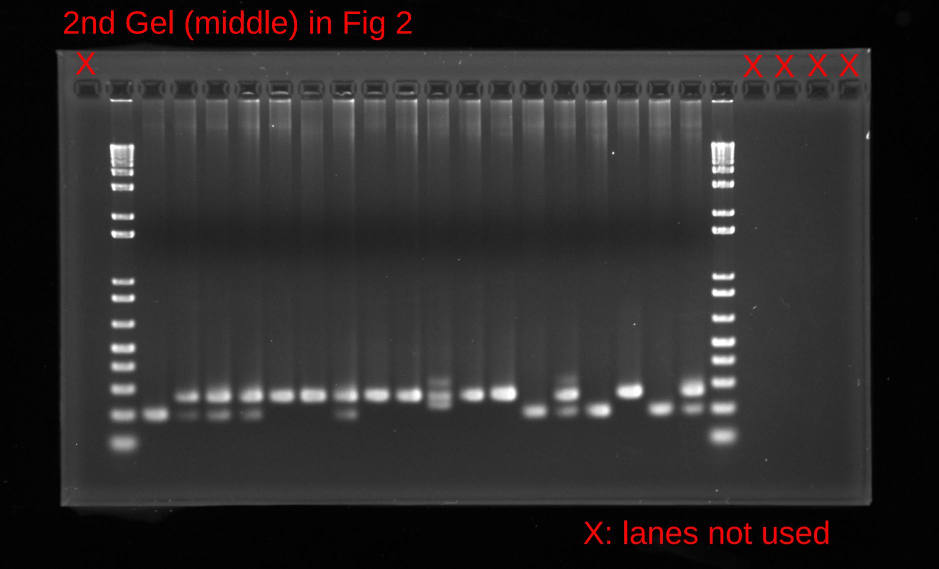
2nd Gel (middle) in Fig 2
X
X X X X
X: lanes not used

3rd Gel (bottom) in Fig 2

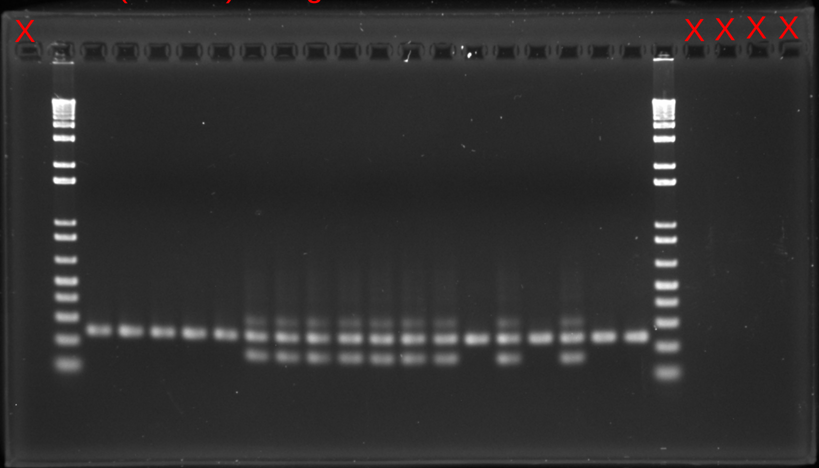

X: lanes not used

1st Gel (top) in Fig 8

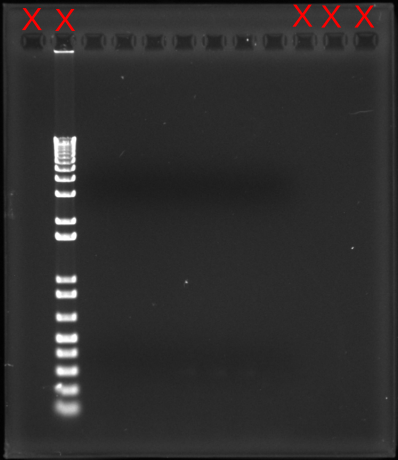

X: lanes not used

2nd Gel (middle) in Fig 8

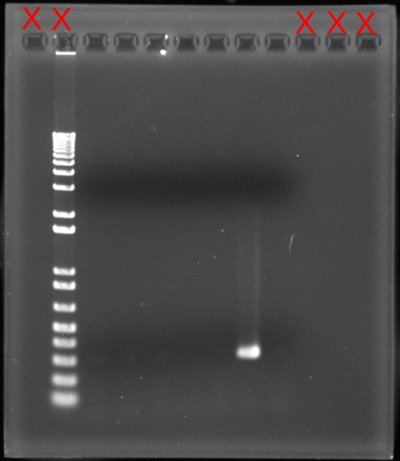

X: lanes not used

3rd Gel (bottom) in Fig 8

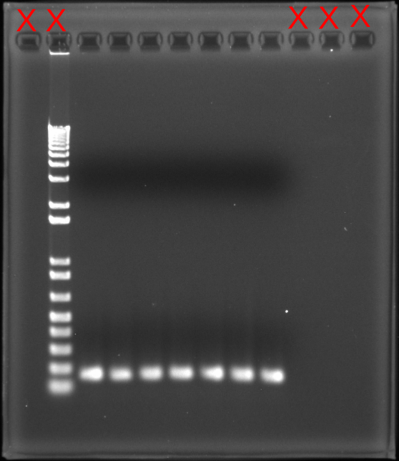

X: lanes not used

Gel in Fig 12A

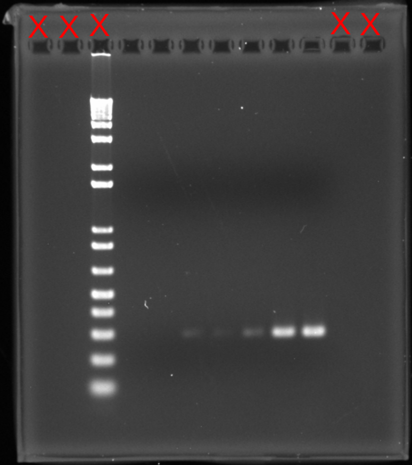

X: lanes not used
