# Supplementary figures and images for: Characterization of testis-specific serine/threonine kinase 1-like (TSSK1-like) gene and expression patterns in diploid and triploid Pacific abalone (Haliotis discus hannai; Gastropoda; Mollusca) males
Source: PLoS One. 2019 Dec 11;14(12):e0226022. doi: 10.1371/journal.pone.0226022 (PMC6905558; doi:10.1371/journal.pone.0226022)

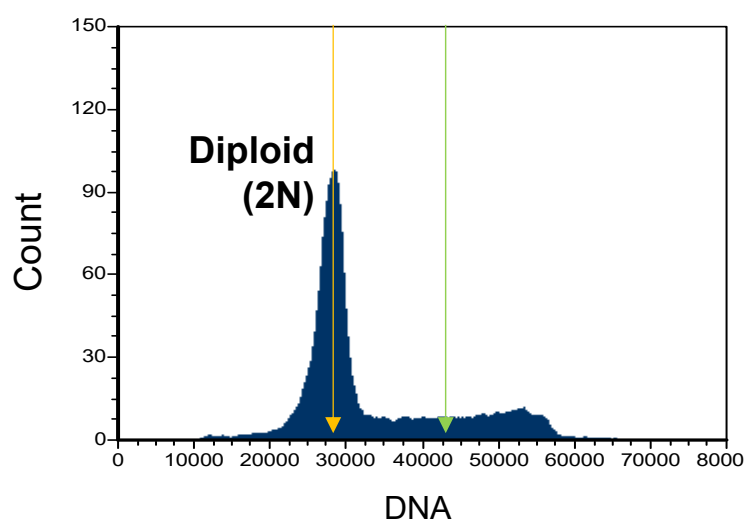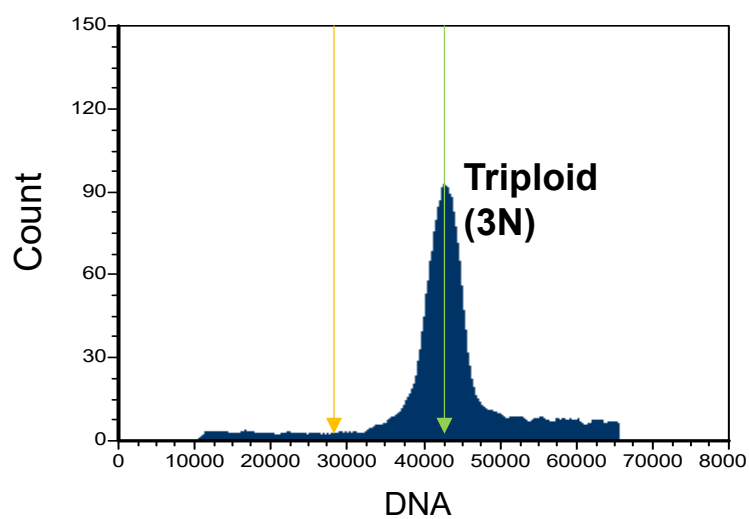

Supplement: S1 Fig — Flow cytometry was performed using a CyFlow Ploidy Analyzer (Sysmex, UK) in propidium iodide (PI)-stained mantle cells. (PDF) [file pone.0226022.s002.pdf]

*Haliotis discus hannai*  
TSSK1-like

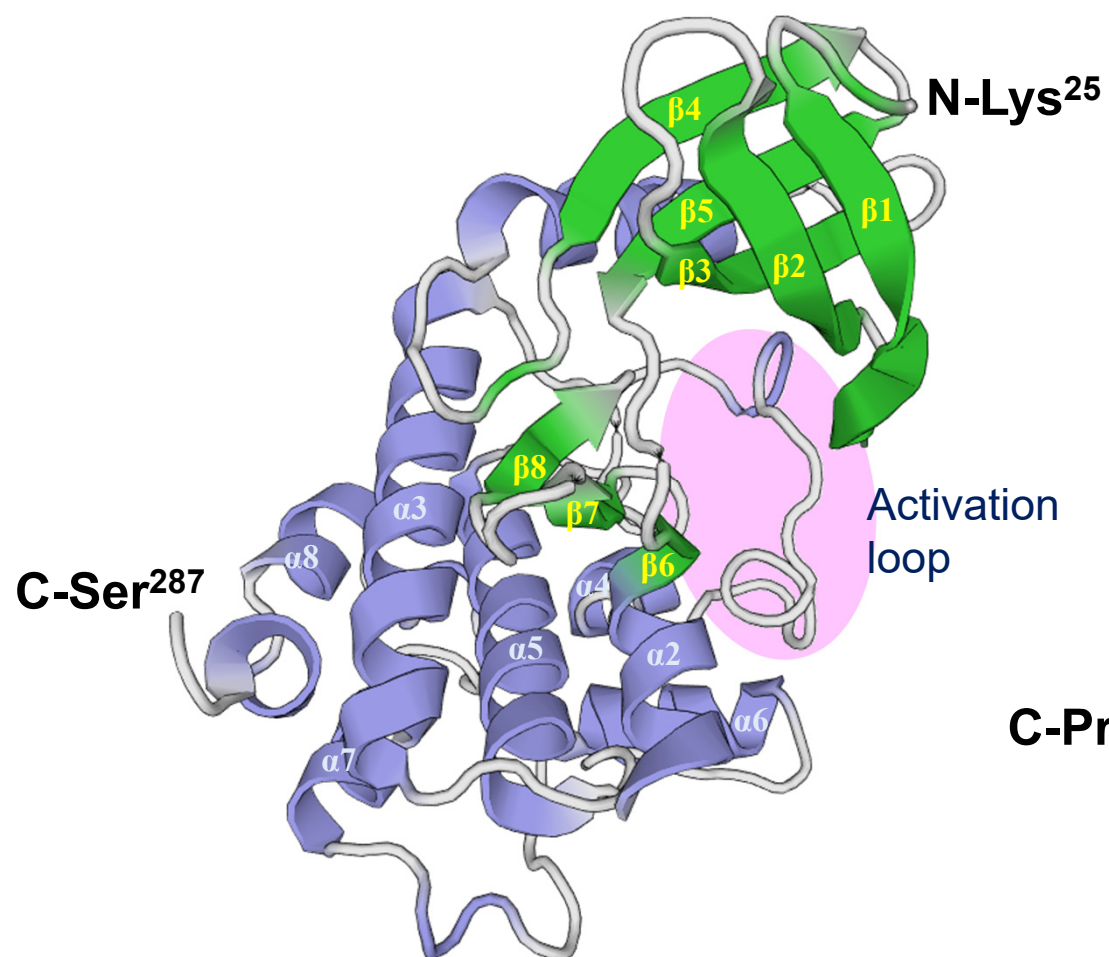

*Homo sapiens* TSSK1B

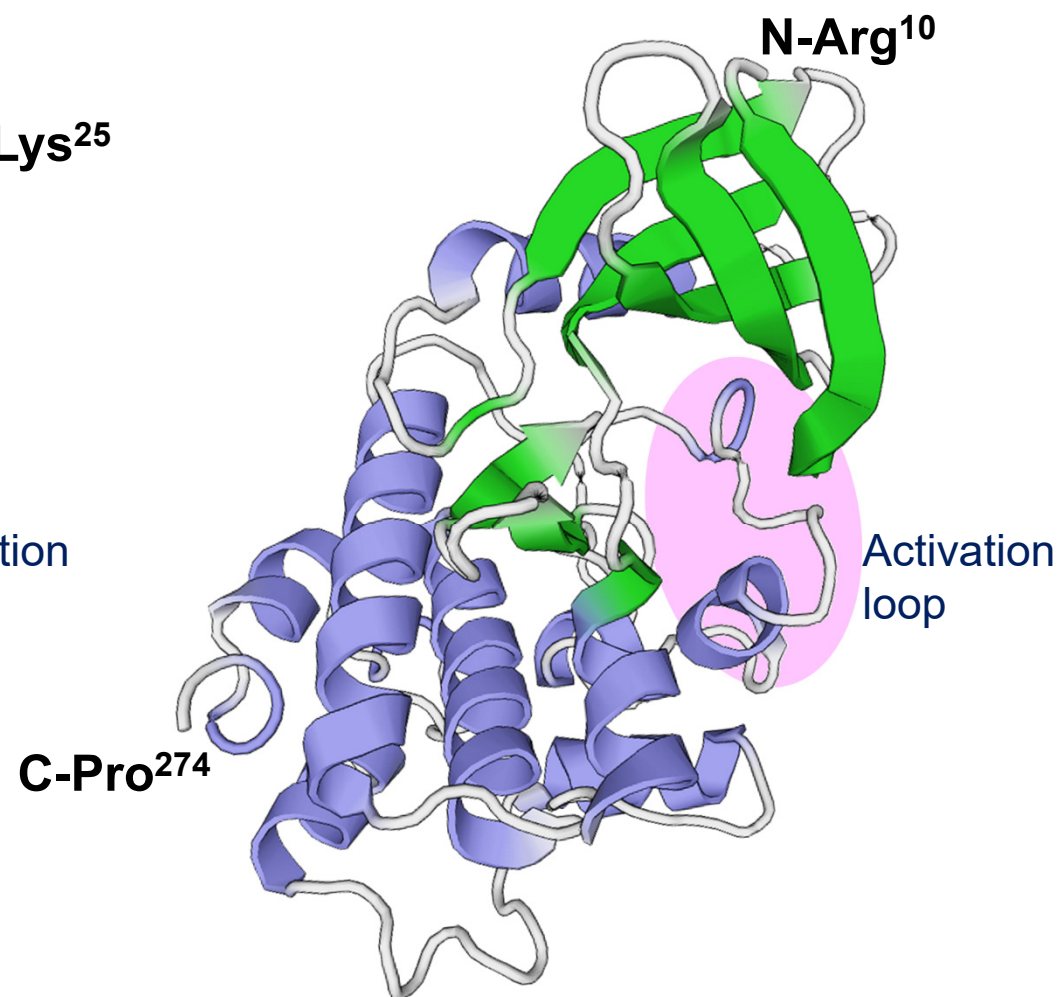

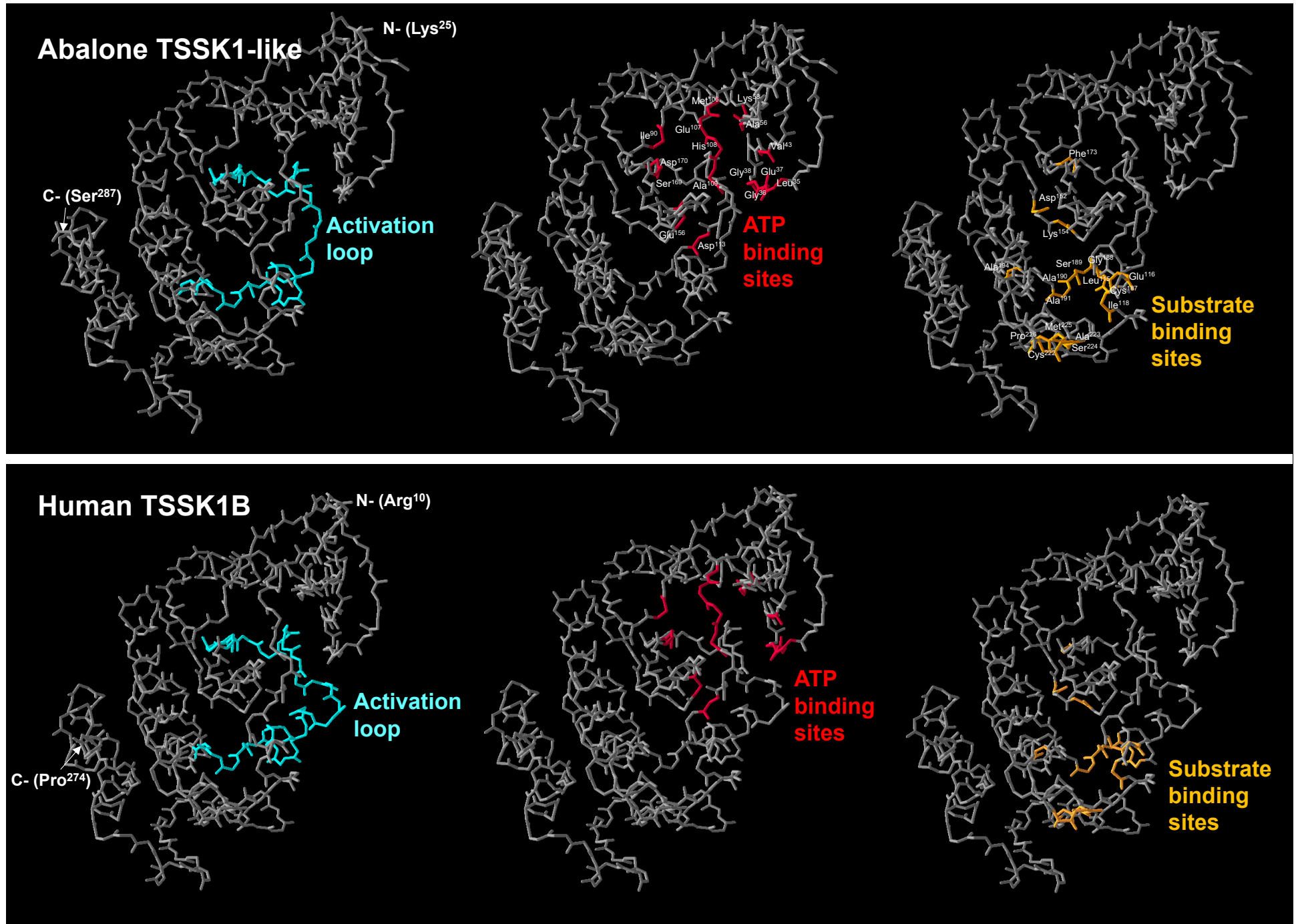

Supplement: S4 Fig — Models were built using the template (2hak.6) in ExPASy SWISS-MODEL (https://swissmodel.expasy.org/). Overall shape and topology, in particular α-helix and β-spread sheet structures (A), and some essential regions such as putative ATP binding sites, substrate binding sites, and the activation loop region (B) of abalone TSSK1-like were compared to human TSSK1B using the Swiss-PdbViewer (ver. 4.1.0; http://www.expasy.org/spdbv/). (PDF) [file pone.0226022.s005.pdf]

S7 Fig

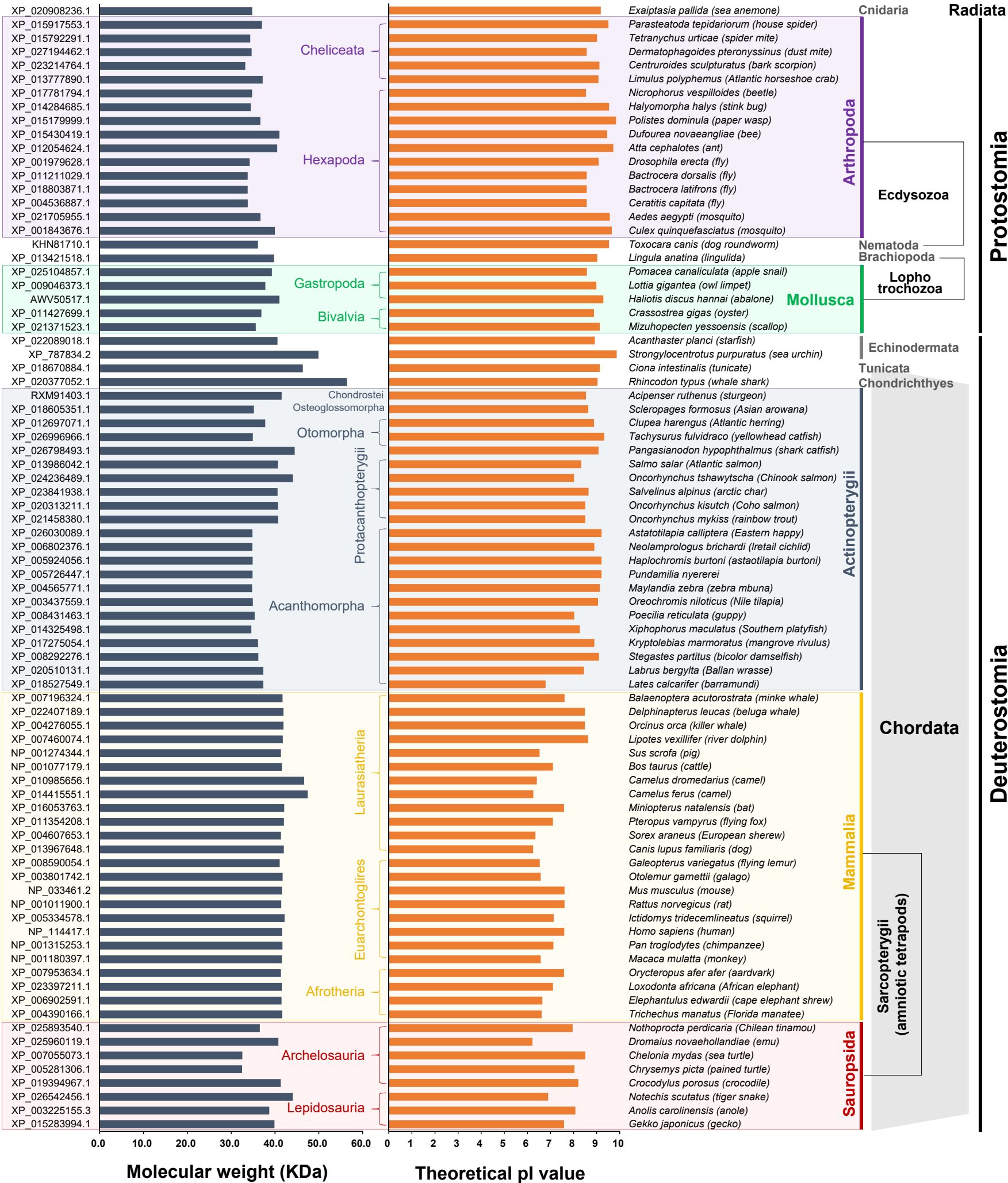

Supplement: S7 Fig — Molecular weights and pI values are estimated using the ExPASy ProtParam tool (https://web.expasy.org/protparam/). (PDF) [file pone.0226022.s008.pdf]

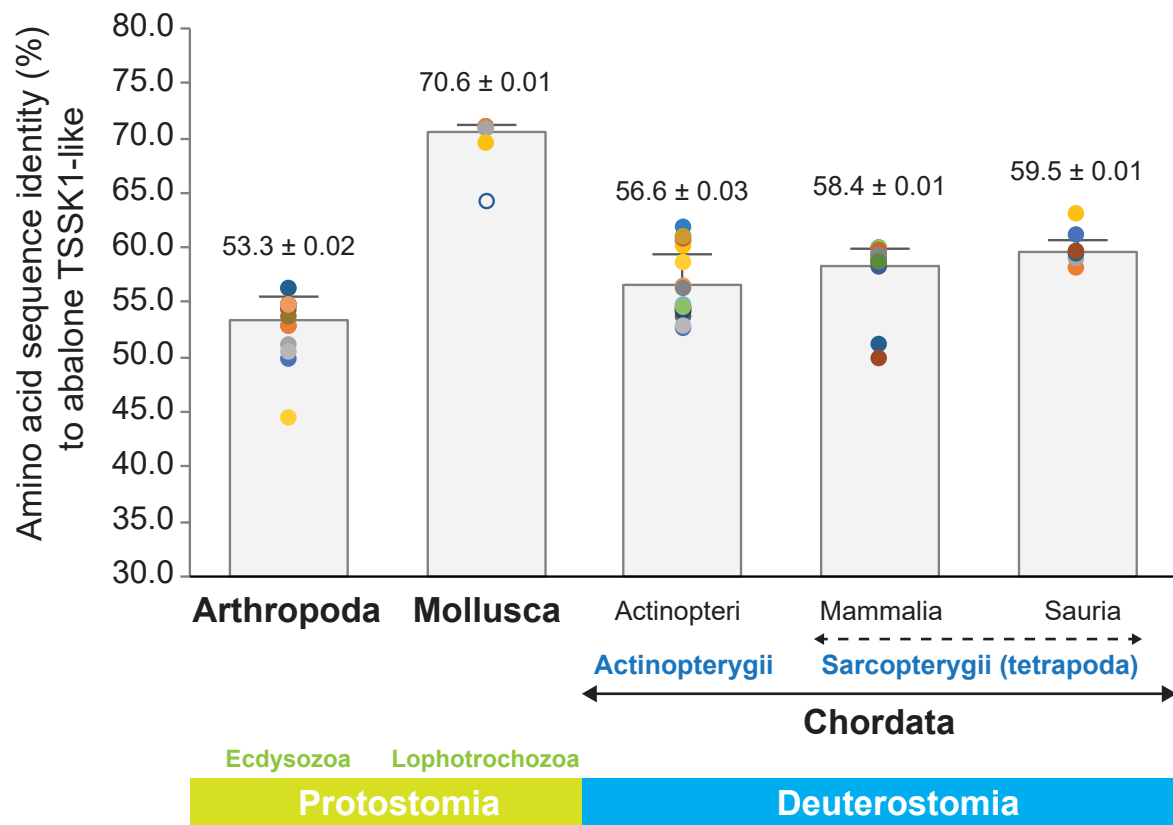

Supplement: S8 Fig — Sequence identity was calculated with an equation [Identity (%) = (number of identical residues/length of multiple sequence alignment) × 100] using a web tool (SIAS; http://imed.med.ucm.es/Tools/sias.html). T-bar on each histogram is the standard deviation. In the molluscan histogram, the identity with a brachiopod sequence is also indicated with open circle. Sequence identity of each ortholog to abalone TSSK1-like is also provided in S2 Table. (PDF) [file pone.0226022.s009.pdf]

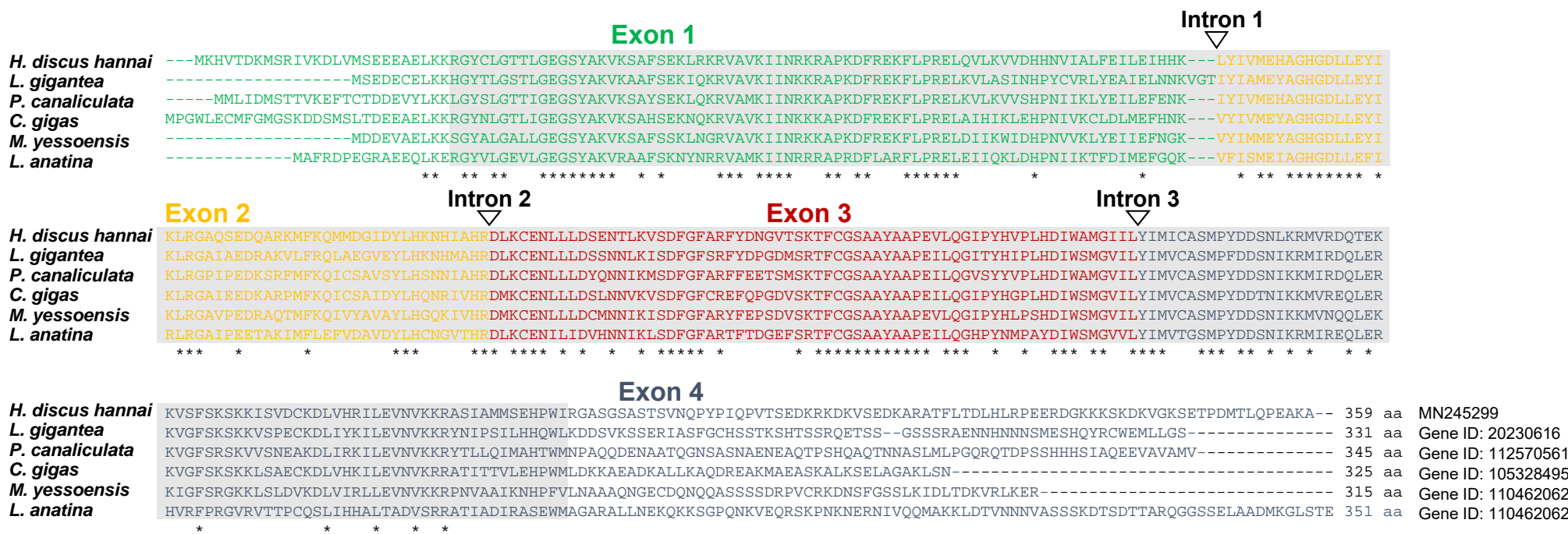

Supplement: S9 Fig — In multiple sequence alignments, conserved amino acid residues are indicated by asterisks, and the putative STKc domain are shaded grey. (PDF) [file pone.0226022.s010.pdf]

S10 Fig

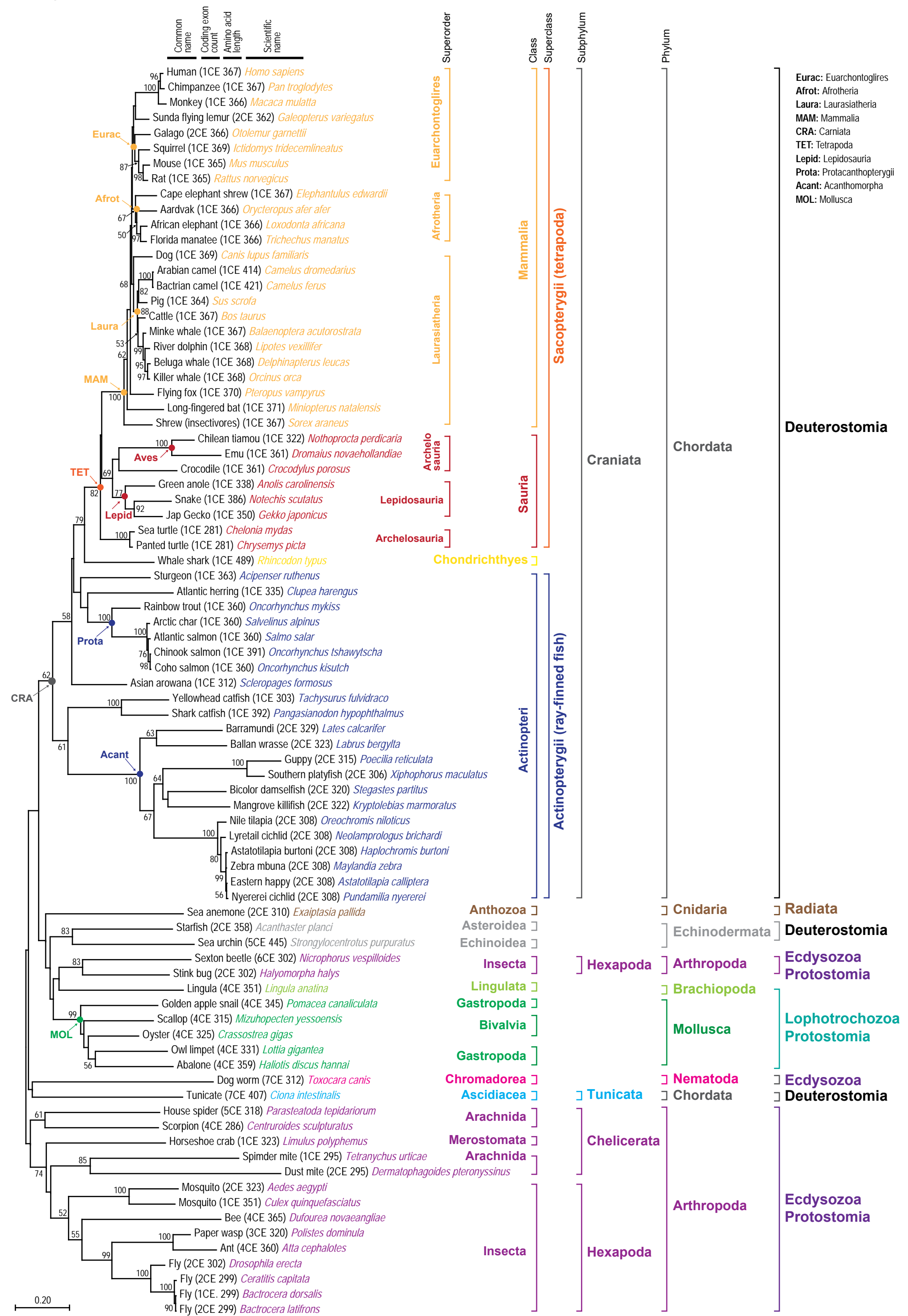

Supplement: S10 Fig — Information on the sequence from each taxon is referred to in S2 Table. (PDF) [file pone.0226022.s011.pdf]

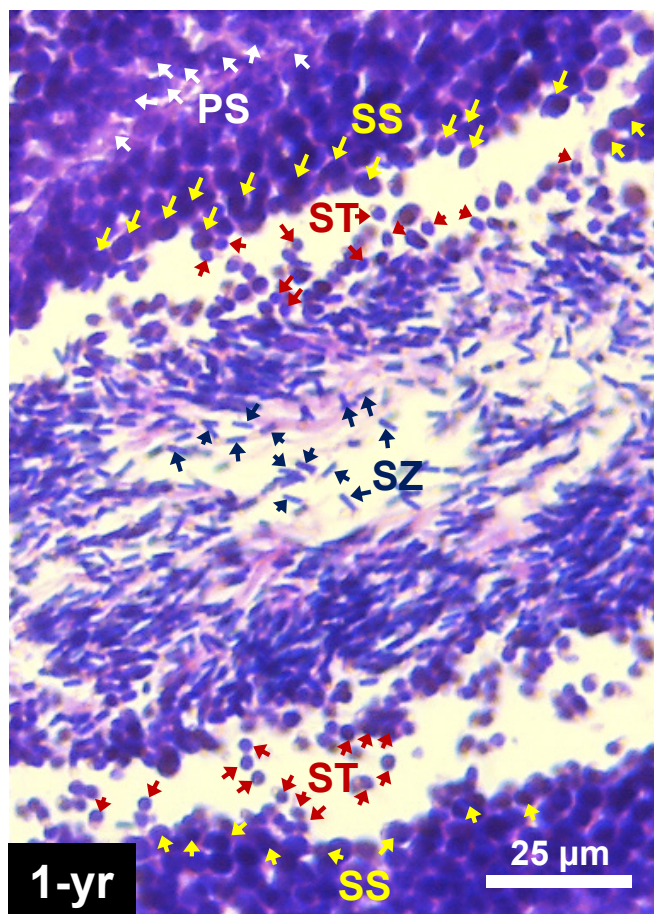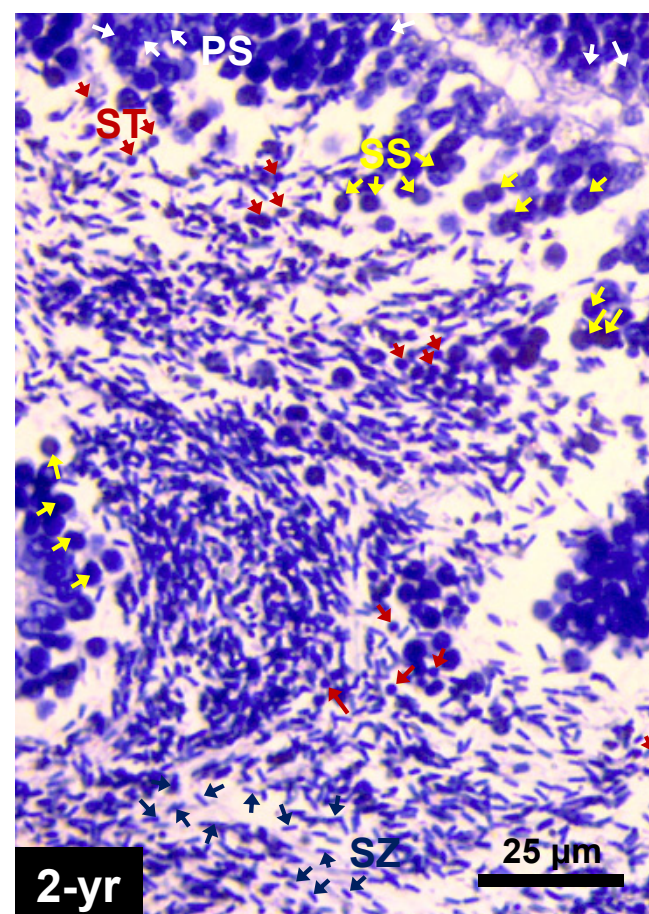

Supplement: S11 Fig — Histological samples were embedded in paraplast, sectioned at 6 μm thickness, and stained with Mayer’s hematoxylin-eosin. Abbreviations are primary spermatocyte (PS; indicated by white arrows), secondary spermatocyte (SS; yellow arrows), spermatid (ST; red arrows), and morphologically differentiated spermatozoon (SZ; blue arrows). Under our culture conditions, full maturation of male abalone is usually obtained from 2 years old. The year classes (1-year-old and 2-year-old) presented here were produced in same month (June) of another year and sampled on the same day during spawning season. (PDF) [file pone.0226022.s012.pdf]

A)

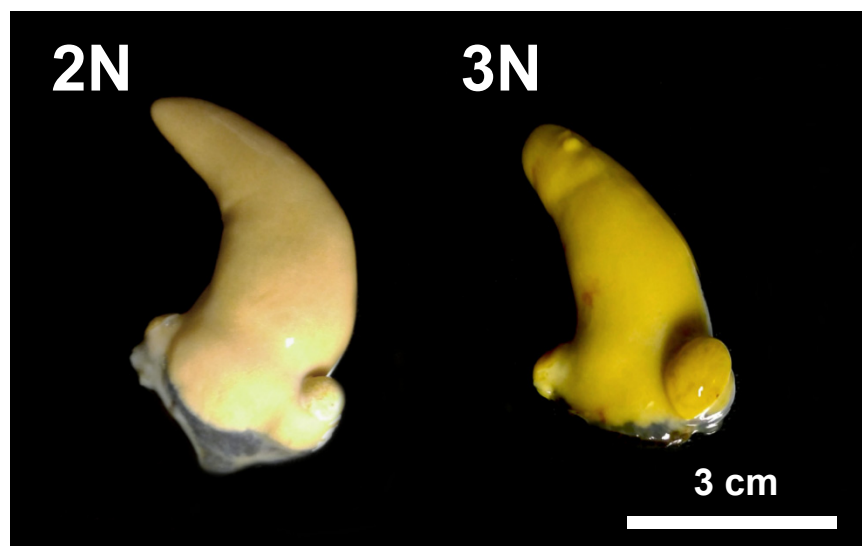

B)

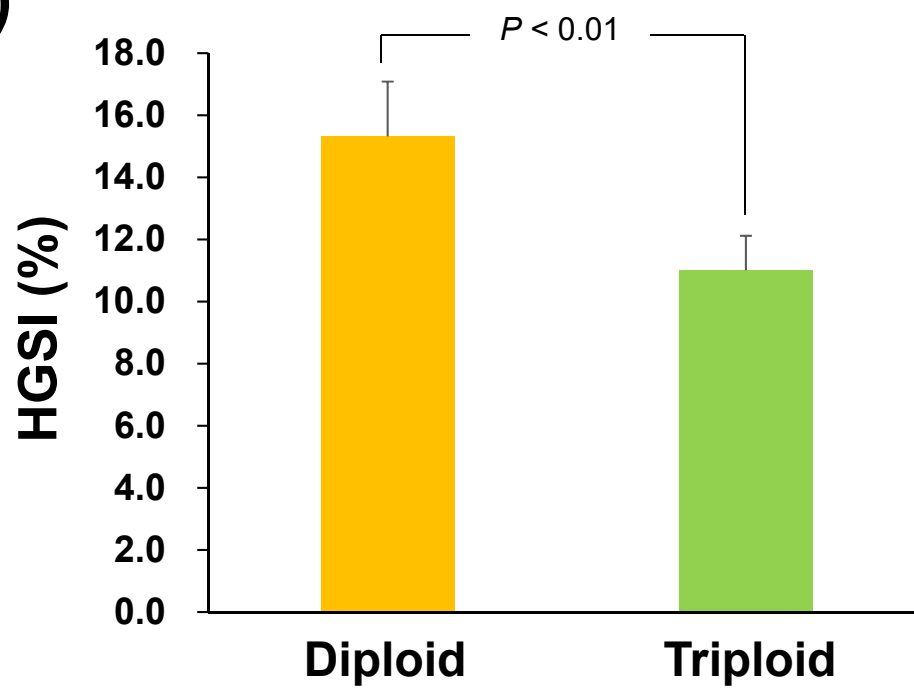

Supplement: S12 Fig — (A) Representative photograph showing external appearance of partially mature triploid testis, as compared to fully matured diploid testis. (B) Hepatopancreas-gonadosomatic index (HGSI) scores of triploid males (N = 18), for which testicular appearances were similar to that shown in (A), were compared with scores of mature diploid males (N = 18) in (B). Statistical difference in HGSI score between diploids and triploids was found based on Student’s t-test. (PDF) [file pone.0226022.s013.pdf]

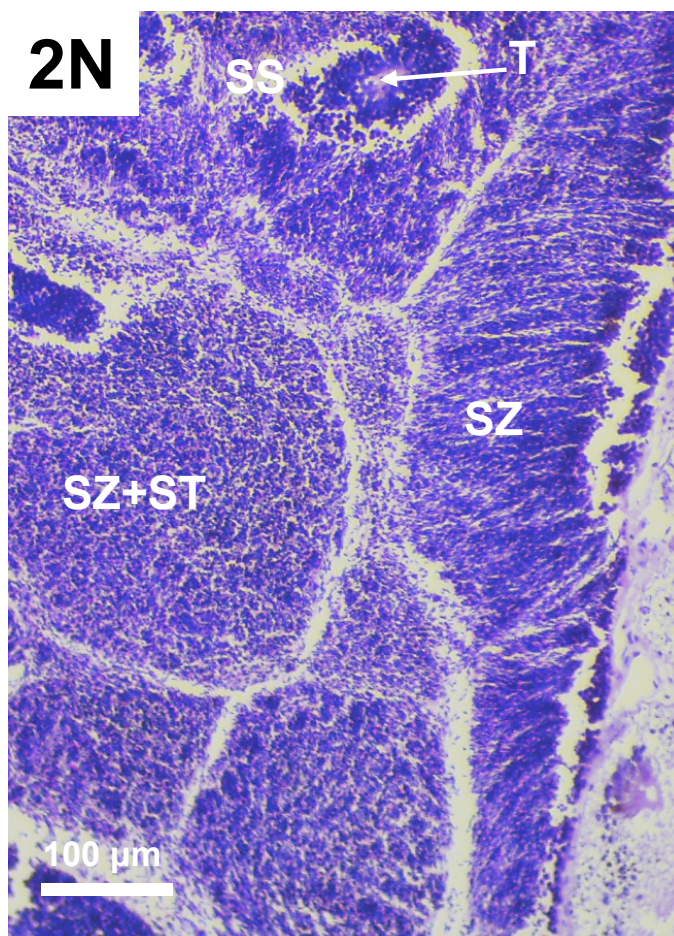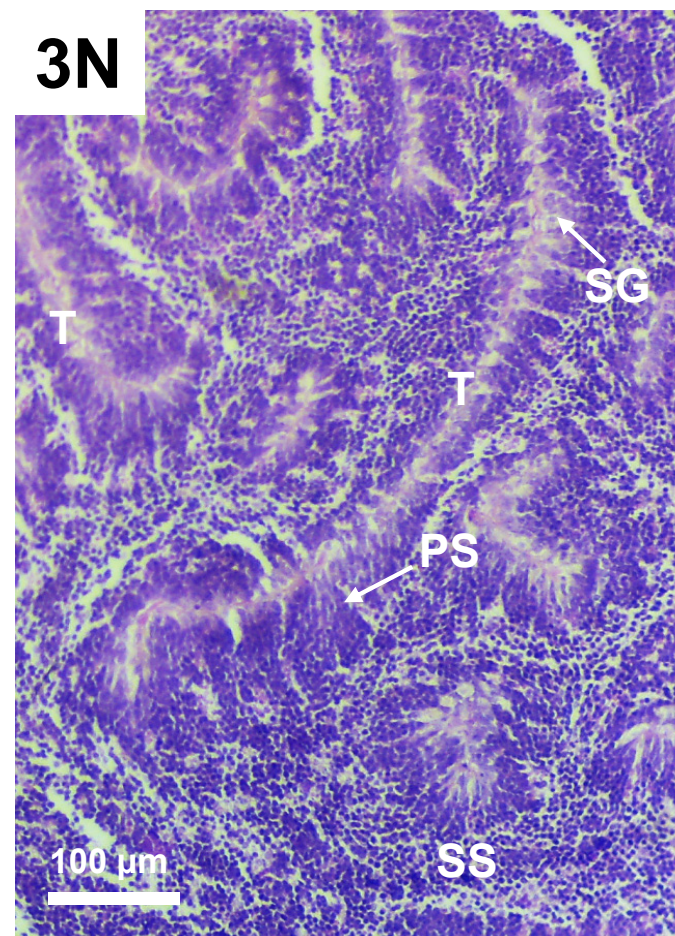

Supplement: S13 Fig — Abbreviations are spermatogonia (SG), primary spermatocyte (PS), secondary spermatocyte (SS), spermatid (ST), spermatozoon (SZ), and trabecula (T). (PDF) [file pone.0226022.s014.pdf]

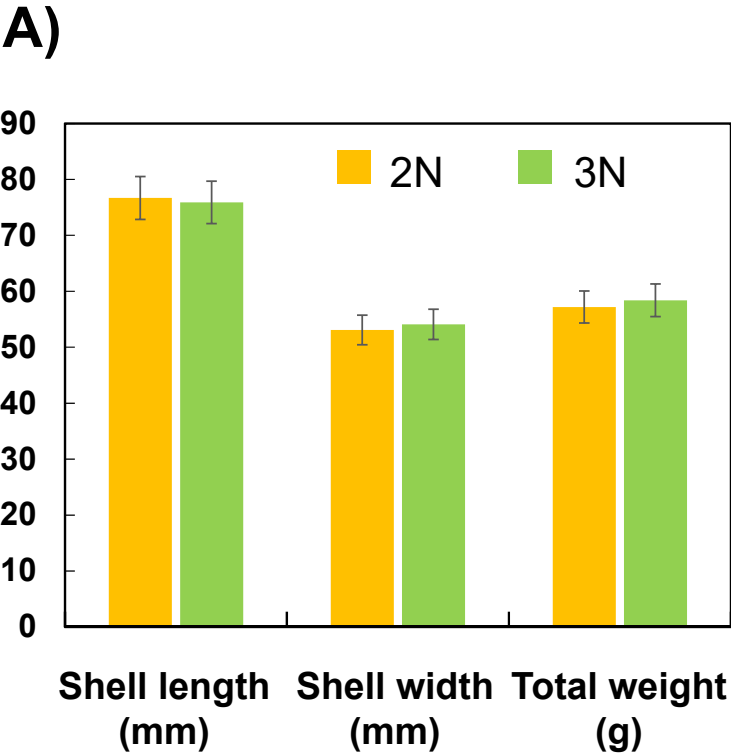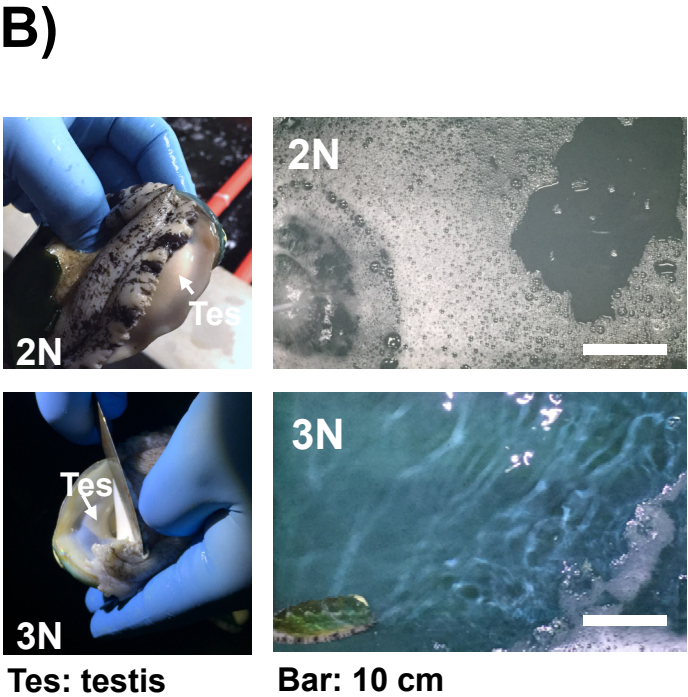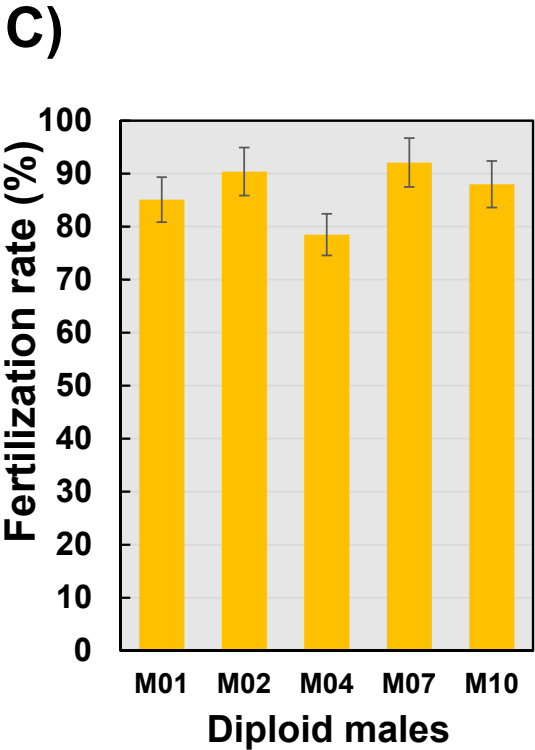

Supplement: S14 Fig — (A) Body size and weights of diploid and triploid males subjected to induced sperm release. No significant difference was found between diploid and triploid males (N = 10 each). (B) Representative photographs showing external appearance of testis development in diploid and triploid males. Sperm release in response to UV-irradiated sea water treatment was typically observed with diploid males, but not triploid males. (C) Fertilization rates (%) of spermatozoa released from diploid males shown as the percentage of embryos commencing successful early cleavages (i.e., 2-cell to 4-cell stages) at 2–3 h post-insemination. Mean ± SDs were calculated based on triplicate estimations using at least 110 embryos for each male. (PDF) [file pone.0226022.s015.pdf]
