# Supplementary material for: Characterization of testis-specific serine/threonine kinase 1-like (TSSK1-like) gene and expression patterns in diploid and triploid Pacific abalone (Haliotis discus hannai; Gastropoda; Mollusca) males
Source: PLoS One. 2019 Dec 11;14(12):e0226022. doi: 10.1371/journal.pone.0226022 (PMC6905558; doi:10.1371/journal.pone.0226022)
Supplement: S2 Fig — (A) Non-synonymous single nucleotide polymorphisms (SNPs) observed in coding region. (B) Major length polymorphisms in intron 1. A large insertion/deletion and TAG-microsatellite locus are notable in intron 1. Within intron 1, base pair numbering is made in accordance with the reference sequence from the abalone individual #1. PCR primers (INT1-1F/1R) to detect individual variations for length polymorphisms are indicated in the above diagram. (C) Length polymorphisms detected in intron 2. Relative positions of three main loci associated with insertion/deletion (in/del) in intron 2 are indicated in the diagram. Multiple sequence alignments of those polymorphic loci. (D) A minisatellite detected in intron 3 of abalone Haliotis discus hannai TSSK1-like gene. A 27-bp unit sequence repeating different numbers among individuals (as shown, 62 times). The consensus sequence (CTCGCAGTACGATGCCAGTGKTACCC) of the 27-bp repetitive unit is drawn using Web Logo (http://weblogo.berkeley.edu/logo.cgi). (PDF) [file pone.0226022.s003.pdf]

S2A Fig

(Nonsynonymous substitutions in coding region)

```
   1 ATGAAACATGTAACAGACAAAATGAGTCGTATTGTCAAAGATTTA
           G
      M  K  H  V  T  D  K  M  S  R  I  V  K  D  L
            D

  46 GTCATGTCGGAGGAAGAAGCGGAGCTGAAGAAAAGGGGATATTGT
        G
      V  M  S  E  E  E  A  E  L  K  K  R  G  Y  C
         V

  91 CTCGGAACTACCCTTGGAGAAGGTTCTTATGCTAAGGTGAAGAGC
      L  G  T  T  L  G  E  G  S  Y  A  K  V  K  S

 136 GCGTTTTCCGAGAAGCTTCGCAAGAGGGTTGCGGTCAAAATTATT
      A  F  S  E  K  L  R  K  R  V  A  V  K  I  I

 181 AATCGCAAACGCGCGCCAAAGGACTTTCGGGAAAAATTTCTGCCG
      N  R  K  R  A  P  K  D  F  R  E  K  F  L  P

 226 CGGGAACTTCAAGTGTTGAAAGTAGTGGATCATCATAATGTTATC
              G                             A
      R  E  L  Q  V  L  K  V  V  D  H  H  N  V  I
               E                             I

 271 GCACTGTTTGAAATCCTGGAGATTCATCACAAGTTATACATAGTG
      A  L  F  E  I  L  E  I  H  H  K  L  Y  I  V

 316 ATGGAGCATGCAGGTCACGGTGACCTCTTGGAGTATATAAAATTG
      M  E  H  A  G  H  G  D  L  L  E  Y  I  K  L

 361 AGGGGGGCCCAGTCTGAAGATCAAGCCCGCAAGATGTTCAAGCAG
      R  G  A  Q  S  E  D  Q  A  R  K  M  F  K  Q

 406 ATGATGGATGGGATAGATTATCTCCACAAGAATCACATAGCCCAC
      M  M  D  G  I  D  Y  L  H  K  N  H  I  A  H

 451 AGGGATCTCAAGTGCGAGAACCTTCTGCTAGACAGTGAGAACACA
      R  D  L  K  C  E  N  L  L  L  D  S  E  N  T

 496 CTCAAGGTATCGGACTTTGGCTTTGCCCGGTTCTATGACAACGGA
      L  K  V  S  D  F  G  F  A  R  F  Y  D  N  G

 541 GTCACCAGTAAGACATTCTGTGGAAGTGCTGCCTACGCAGCTCCG
      V  T  S  K  T  F  C  G  S  A  A  Y  A  A  P

 586 GAAGTTCTCCAAGGTATCCCGTATCACGTGCCTCTTCATGACATC
      E  V  L  Q  G  I  P  Y  H  V  P  L  H  D  I

 631 TGGGCAATGGGGATCATCCTCTATATTATGATATGTGCCTCAATG
      W  A  M  G  I  I  L  Y  I  M  I  C  A  S  M

 676 CCGTACGACGACTCGAACCTGAAGCGCATGGTCAGAGACCAAACT
      P  Y  D  D  S  N  L  K  R  M  V  R  D  Q  T

 721 GAAAAGAAGGTCAGTTTCTCCAAGTCCAAGAAGATCTCCGTGGAT
      E  K  K  V  S  F  S  K  S  K  K  I  S  V  D

 766 TGTAAGGATTTGGTACACCGAATACTCGAGGTGAACGTCAAGAAA
      C  K  D  L  V  H  R  I  L  E  V  N  V  K  K

 811 CGCGCCTCCATTGCCATGATGTCGGAGCATCCGTGGATAAGAGGA
      R  A  S  I  A  M  M  S  E  H  P  W  I  R  G

 856 GCCAGTGGCTCCGCAAGCACATCAGTGAACCAGCCATACCCAATA
      A  S  G  S  A  S  T  S  V  N  Q  P  Y  P  I

 901 CAGCCAGTCACCAGCGAGGACAAGAGGAAGGACAAAGTGAGCGAA
      Q  P  V  T  S  E  D  K  R  K  D  K  V  S  E

 946 GACAAAGCGCGCGCAACATTTCTGACTGACCTTCACCTTCGACCG
            T
      D  K  A  R  A  T  F  L  T  D  L  H  L  R  P
            V

 991 GAAGAAAGAGATGGCAAAAAGAAGTCCAAGGACAAAGTTGGAAAG
      E  E  R  D  G  K  K  K  S  K  D  K  V  G  K

1036 AGTGAGACGCCGGATATGACGCTTCAGCCGGAGGCTAAAGCGTGA
      S  E  T  P  D  M  T  L  Q  P  E  A  K  A  *
```

S2B Fig

(Intronic length polymorphism: Intron 1)

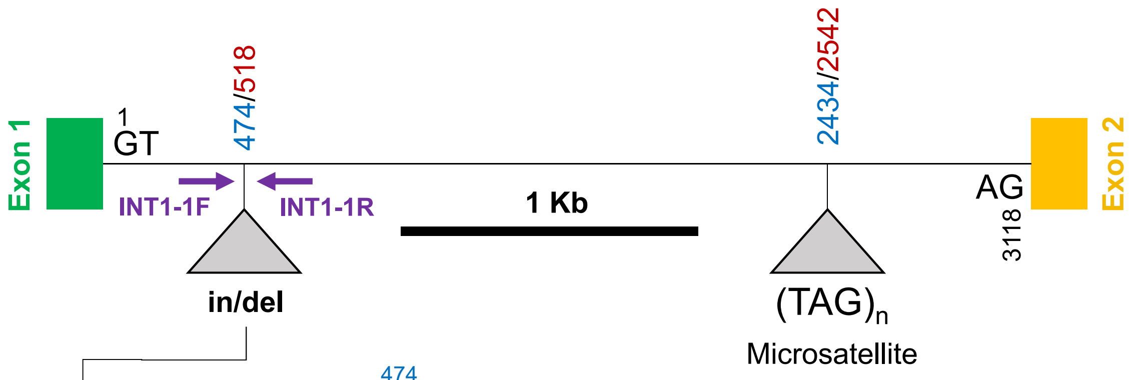

474

```
Abalone-1 TTATAGCTCTGTGGTGTTATAACGGAAGAAAA-CTTACGTTTCGACTC-GTCGAAA----
Abalone-2 TTATAGCTCTGTGGTGTTATAACGGAAGAAAA-CTTACGTTTCGACTC-GTCGAAA----
Abalone-3 TTATAGCTCTGTGGTGTTATAACGGAAGAAAA-CTTACGTTTCGACTC-GTCGAAA----
Abalone-4 TTATAGCTCTGTGGTGTTATAAAGGACGAAAAACTCGTGAAACTACTTTGTCTGAATATC
          ********************** *** ***** **    *    * ***   ***  **

Abalone-1 ----------AAAACCTCGTG---------------------------------------
Abalone-2 ----------AAAACCTCGTG---------------------------------------
Abalone-3 ----------AAAACCTCGTG---------------------------------------
Abalone-4 GGTTTTCTATAAGACTTCGTGTTTGAAAAGTTTTAAATATAAGTAAATTATGTCTCACCT
                    ** ** *****

Abalone-1 ----------AAAGGACTTTGTCT------------------------------------
Abalone-2 ----------AAAGGACTTTGTCT------------------------------------
Abalone-3 ----------AAAGGACTTTGTCT------------------------------------
Abalone-4 ATCTGTACCAAAACGATTTGGTCTTACATATAATAATTCTTTTCTAACCTTATAGTGAGA
                    *** ** ** ****

Abalone-1 -----------------------------------------------GAAT---------
Abalone-2 -----------------------------------------------GAAT---------
Abalone-3 -----------------------------------------------GAAT---------
Abalone-4 AACTGATCCAATAGCGTAGCTCAGTCCGCCACCATATATAATTGCTAGAATACTTTTCAG
                                                         ****

Abalone-1 ---GTCGGTTTTTTCAC-------------------------------------------
Abalone-2 ---GTCGGTTTTTTCAC-------------------------------------------
Abalone-3 ---GTCGGTTTTTTCAC-------------------------------------------
Abalone-4 TGCGTCGAACTACTCACTCACTCACCCAATGACAAGACGTTGAATTATCCTTATTTACTA
             ****    *   ****
                                      518
Abalone-1 ----------------------------CCCTATAATAAGTACTGTGTCTTCCTCGATTG
Abalone-2 ----------------------------CCCTATAATAAGTACTGTGTCTTCCTCGATTG
Abalone-3 ----------------------------CCCTTTAATAAGTACTGTGTCTTCCTCGATTG
Abalone-4 TATAAACTGGTAAAGTGATTATATCACCCCTTATAATAAGGATTGTGTCTTCCTCGATTG
                                      ** *  ******* *  *****************
```

2434

```
Abalone-1 TAGTAGTAGTAGTAGTAGTAGTAGTAGTAGTAGTAGTAGTAGTAGTAGTAGTAGTAG
Abalone-2 TAGTAGTAGTAGTAGTAGTAGTAGTAGTAGTAGTAGTAGTAGTAGTTGTAGCAGTAG
Abalone-3 TAGTAGTAGTAGTAGTAGTAGTAGTAGTAGTAGTAGTAGTAGTAGTTGTAGCAGTAG
Abalone-4 TAGTAGTAGTAGTAGTAGTAGTAGTAGTAGTAGTAGTAGTAGTAG---------

                                                          2542
Abalone-1 TAGTAGTAGTAGTAGTAGTAGTTTTTAGTTGTAGCAGCAGTAGTAGTAGTAGTAG
Abalone-2 TAGTAGTAGTAGTAGTAGTAG----------------------------------
Abalone-3 TAGTAGTAGTAGTAGTAGTAG----------------------------------
Abalone-4 ---------------------TTTTTAGTTGAAGCAGTAGTAGTAGTAG------
```

INT1-1F: 5´-GACCGGTAAGAACAGATCGC-3´

INT1-1R: 5´-GTTGACTCCATCAGGCATTG-3´

Expected size of longer amplicon = 430 bp

Expected size of shorter amplicon = 197 bp

S2C Fig

(Intronic length polymorphism: Intron 2)

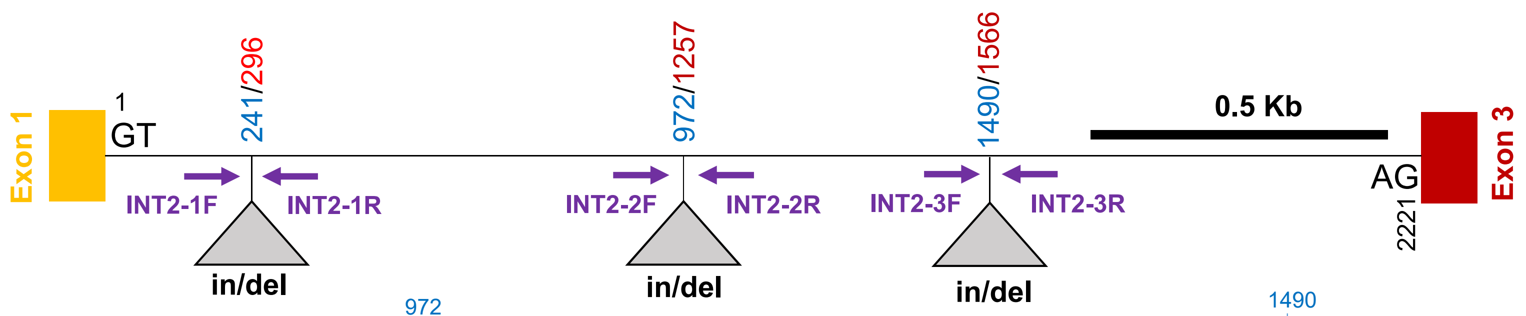


```
241
Abalone-1 CCTGAGGCGCTGCAGTTCGTTCTGAAGAGT
Abalone-2 CCTGAGGCGCTGCAGTTCGTTCTGAAGAGT
Abalone-3 CCTGAGGCGCTGCAGTTCGTTCTGAAGAGT
Abalone-4 TCTGAGGCGCTGCAGTTCGTTCTGAAGAGT
           *****************************

Abalone-1 TGCGTCCCTTAGCCATGCCAGAATC-----
Abalone-2 TGCGTCCCTTAGCCATGCCAGAATC-----
Abalone-3 TGCGTCCCTTAGCCATGCCAGAATC-----
Abalone-4 TGCGTCCCTTAGCCATACCAGAATCAAGCT
          **************** ********

Abalone-1 ------------------------------
Abalone-2 ------------------------------
Abalone-3 ------------------------------
Abalone-4 CCTCACAGTGTACCGAGAGTGTAACGAAGG

Abalone-1 ------------------------------
Abalone-2 ------------------------------
Abalone-3 ------------------------------
Abalone-4 TGTCACGCGCTGGGAAGGAGCTTGTACCAG

Abalone-1 ----CAAGCTCCGCACAGCGATAGCCCGTT
Abalone-2 ----CAAGCTCCKCACAGCGATAGCCCGTT
Abalone-3 ----CAAGCTCCTCACAGCGATAGCCCGTT
Abalone-4 AATCCGAGCTCCTCACAGCGATAGCCCGTT
              * ****** *****************
                                         296
Abalone-1 AGCGGAGCTCTCTAATGCTATCCGCTGCCA
Abalone-2 AGCGGAGCTCTCTAATGCTATCCGCTGCCA
Abalone-3 AGCGGAGCTCTCTAATGCTATCCGCTGCCA
Abalone-4 CACGGAGCTCTCTGATGCTATCCGCGGCCA
            *********** *********** ****
```

```
972
ACATTAAGTGAGAGAGTGAGTGAGTTGGGTTTAACGCCGCTTTTAACAGTATTCCAGCTG
ACATTAAGTGAGAGAGTGAGTGAGTTGGGTTTAACGCCGCTTTTAACAGTATTCCAGCTG
ACATTAAGTGAGAGAGTGAGTGAGTTGGGTTTAACGCCGCTTTTAACAGTATTCCAGCTG
CCATTAAGTGAGAGAGTGAGTGAGT-----------------------------------
 ************************

TATCGCGGCGTGTCAGCTTATTTTGGGAGGAAAGCCCCAAGTGATGCAGGCATCAGACGT
TATCGCGGCGTGTCAGCTTATTTTGGGAGGAAAGCCCCAAGTGATGCAGGCATCAGACGT
TATCGCGGCGTGTCAGCTTATTTTGGGAGGAAAGCCCCAAGTGATGCAGGCATCAGACGT
--------------------------------------GAGTGAGTTGGGTTTTAAGC--
*****    **   *  *   *

TTACCACTTCGGCGAAACCCACGAGCACGGGTATGGTGCT-ACGAACCTAGACACATAAT
TTACCACTTCGGCGAAACCCACGAGCACGGGTATGGTGCT-ACGAACCTAGACACATAAT
TTACCACTTCGGCGAAACCCACGAGCACGGGTATGGTGCTGACAAACCTAGACACATAAT
----CGCT----------------------------------------------------
    * **

CGGGGCGAACCAATAAATCAAATATTATAGCGATCGCGAGAACAATATCTACTGGTCAAC
CGGGGCGAACCAATAAATCAAATATTATAGCGATCGCGAGAACAATATCTACTGGTCAAC
CGGGGCGAACCAATAAATCAAATATTATAGCGATCGCGAGAACAATATCTACTGGTCAAC
------------------------------------------------------------

ATCCGTGGGCCTACCAATTTCGATCAGCAGATTATGGCATCCGCATCTGACTCAGTGTAG
ATCCGTGGGCCTACCAATTTCGATCAGCAGATTATGGCATCCGCATCTGACTCAGTGTAG
ATCCGTGGGCCTACCAATTTCGATCAGCAGATTATGGCATCCGCATCTGACTCAGTGTAG
-----------------TTT----------------------------------------
                 ***
                                                             1257
TGAGTGAGTTAGAACAGTGATCCAGTTATATCGCGGCGTGTCAGCTTAATGTGGGAGGAA
TGAGTGAGTTAGAACAGTGATCCAGTTATATCGCGGCGTGTCAGCTTAATGTGGGAGGAA
TGAGTGAGTTAGAACAGTGATCCAGTTATATCGCGGCGTGTCAGCTTAATGTGGGAGGAA
------------AACAGTATTCCAGTTATATCGCGGCGTGTCAGCTTAATGTGGGAGAAA
            ******  ************************************* **
```

```
1490
CTGGGTCACCCGTGCCCCAGACTCAGTGAA
CTGGGTCACCCGTGCCCCAGACTCAGTGAA
CTGGGTCACCCGTGCCCCAGACTCAGTGAA
CTGG--------------------------
****

TAATACTGATTAATACATGTACGTTGAATA
TAATACTGATTAATACATGTACGTTGAATA
TAATACTGATTAATACATGTATGTTGAATA
------------------------------

TAACATTGACTAAGAGTTAGGTGATATTGA
TAACATTGACTAAGAGTTAGGTGATATTGA
TAACATTGACTAAGAGTTAGGTGATATTGA
---------------------TGATATTGA
                     *********
                                1566
TAGATGTCATCTCTGAATACTGCCAAAAGT
TAGATGTCATCTCTGAATACTGCCAAAAGT
TAGATGTCATCTCTGAATACTGCCAAAAGT
TAGATGCCATCTCTGAATACTGCCAAAAGT
****** ***********************
```

INT2-1F: 5´-TCGTTCTGAAGAGTTGCGTC-3´
INT2-1R: 5´-GGTAAGTGTATCGGCGTCA-3´
Expected size of longer amplicon = 271 bp
Expected size of shorter amplicon = 202 bp

INT2-2F: 5´-CACCTTCCATGTGTGGTTTG-3´
INT2-2R: 5´-CTTTGCAGAGTTGCGTCCCT-3´
Expected size of longer amplicon = 549 bp
Expected size of shorter amplicon = 290 bp

INT2-3F: 5´-AACTCTGCAAAGCGAATCGC-3´
INT2-3R: 5´-TCACGTGTCCCTCATACCAT-3´
Expected size of longer amplicon = 237 bp
Expected size of shorter amplicon = 160 bp

(Minisatellite in Intron 3)

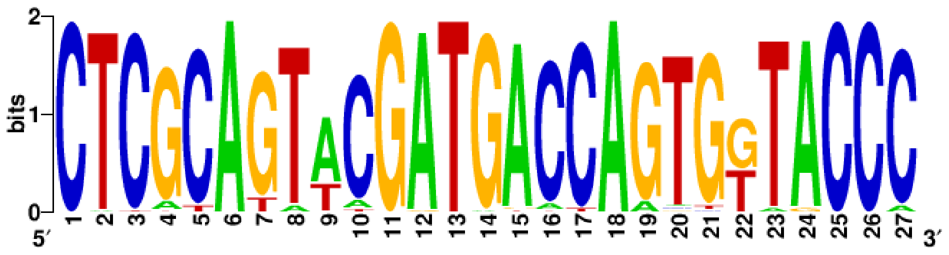

1 9 18 27

CTCGCAGTACGATGACCAGTG**T**TACCC 1
CTCGCAG**AT**CGATGACCAGTG**T**TACCC
CTCGCAGT**T**CGATGACCAGTG**T**TACCC
-TC**T**CAGTACGATGACCAGTGGTACCC
CTCGCAGTACGATGACCAGTGGTACCC 5
CTCGCAGTACGATGACCAGT**TT**TACCC
CTCGCAGTA**T**GATGACCAGTG**T**TACCC
CTCGCAGTACGATGACCAGTGGTACCC
CTCGCAGT**T**CG**G**TGACCAGTGGTACCC
CTCGCAGTACGATGACCAGTG**T**TACC**A** 10
CTCGCAGTACGATGACCAGTGGTACCC
CTCGCAGTACGATGACCAGTGGTACCC
CTC**A**CA**T**TACGATGACCAGTGGTACCC
CTCGCAGT**T**CGATGACCAGTG**T**TACCC
CTCGCAGTACGATGACCAGTGGTACCC 15
CTCGCAGTACGATGACCAGTG**T**TACCC
CTC**AT**AGT**T**CGATGAACAGTGGTACCC
CTCGCAGTACGATGACCAGTGGTACCC
CTCGCAGTACGATGACCAGTGGTACCC
CTCGCAGTA**T**GATGAC**T**AGTG**T**TACCC 20
CTCGCAGTACGAT**A**ACCAGTG**T**TACCC
CTCGCA**T**TACGATGACCAGTGGTACCC
CTCGCAGT**T**CGATGACCAG**G**GGTACCC
CTCGCAGTACGATGACCAGTG**T**TACCC
CTCGCAGTACGATGACCAGTGGTACCC 25
CTCGCAGT**T**CGATGACCAGTGGTACCC
CTCGCAGTACGATGACCAGTG**TA**ACCC
CTC**AT**AGT**T**CGATGA**A**CAGTGGTACCC
CTCGCAGTACGATGACCAATG**T**TACCC
CTCGCA**T**TACGATGACCAGTGGTACCC 30
CTCGCAGTACGATGACCAGTGGTACCC

1 9 18 27

CTC**A**CA**T**TACGATGACCAGTGGTACCC
CTCGCAGTACGATGACCAGTG**T**TACCC
CTCGCAGT**T**CGATGACCAGTG**T**TACCC
CT**T**GCAGTACGATGACCAGTGGTACCC 35
CTCGCAGTACGATGACCAGTG**T**TACC**A**
CTCGCAGTACGATGACCAGTG**T**TACCC
CTCGCAGT**-**CGATGACCAGTGGTACCC
CTCGCA**T**TACGATGACCAGTG**T**TACCC
CTCGCAGTACGATGACCAGTGGTACCC 40
CTCGCAGTA**A**GATGACCAGTGGTACCC
C**A**CGCAGTACGATGACCAGTG**T**TACCC
CTCGCAGTACGATGACCAGTGGTACCC
CTCGCAGTA**A**GATGACCAGTGGTACCC
CTCGCAG**AT**CGATGACCA**A**TG**T**TACCC 45
CTCGCAGT**T**CGATGACCAGTGGTACCC
CTCGCAGT**T**CGATGACCA**A**TGGTACCC
CTCGCAGT**T**CGATGACCAGTG**T**TACC**A**
CTCGCAGTACGATGACCAGT**TTA**ACCC
CTCGCAGTACGATGA**T**CAG**CCT**TACCC 50
CTCGCAGTA**A**GATGACCAGTGGTACCC
CTCGCAGT**T**CGATGACCAGTGGTACCC
CTCGCAGT**T**CGATGACCAGTGGTACCC
CTCGCAGTA**A**GATGACCAGTGGTACCC
CTCGCAG**AT**CGATGACCA**A**TG**T**TACCC 55
CTCGCAGT**T**CGATGACCAGTGGTACCC
CTCGCAGT**T**CGATGACCAGTGGTACCC
CTCGCAGTACGATGACCAGTGGTACCC
CTCGCAGTACGATG**T**CCAGTG**T**TACCC
CTCGCA**T**TACGATGACCAG**A**G**T**T**G**CCC 60
CTCGCA**T**TACGATGACCAGTG**T**T**G**CCC
CTCG**T**AGT**C**CGATG**GAT**A**A**TG**T**TACCC
