# Supplementary material for: Characterization of testis-specific serine/threonine kinase 1-like (TSSK1-like) gene and expression patterns in diploid and triploid Pacific abalone (Haliotis discus hannai; Gastropoda; Mollusca) males
Source: PLoS One. 2019 Dec 11;14(12):e0226022. doi: 10.1371/journal.pone.0226022 (PMC6905558; doi:10.1371/journal.pone.0226022)
Supplement: S3 Fig — Of 385 aligned positions, 134 residues are shared by all three sequences. (PDF) [file pone.0226022.s004.pdf]

S3 Fig

```
                             1                            30                            60
H. sapiens TSSK1             -----------------MDDAAVLKRRGYLLGINLGEGSYAKVKSAYSERLKFNVAIKII
H. sapiens TSSK2             -----------------MDDATVLRKKGYIVGINLGKGSYAKVKSAYSERLKFNVAVKII
H. discus hannai TSSK1-like  MKHVTDKMSRIVKDLVMSEEEAELKKRGYCLGTTLGEGSYAKVKSAFSEKLRKRVAVKII
                                                    *   **  *  ** ********* ** *   ** ***

                             61                           90                           120
H. sapiens TSSK1             DRKKAPADFLEKFLPREIEILAMLNHCSIIKTYEIFETSHGKVYIVMELAVQGDLLELIK
H. sapiens TSSK2             DRKKTPTDFVERFLPREMDILATVNHGSIIKTYEIFETSDGRIYIIMELGVQGDLLEFIK
H. discus hannai TSSK1-like  NRKRAPKDFREKFLPRELQVLKVVDHHNVIALFEILEIHH-KLYIVMEHAGHGDLLEYIK
                              **  * ** * *****   *    *   *   ** *      ** **    ***** **

                             121                          150                          180
H. sapiens TSSK1             TRGALHEDEARKKFHQLSLAIKYCHDLDVVHRDLKCDNLLLDKDFNIKLSDFSFSKRCLR
H. sapiens TSSK2             CQGALHEDVARKMFRQLSSAVKYCHDLDIVHRDLKCENLLLDKDFNIKLSDFGFSKRCLR
H. discus hannai TSSK1-like  LRGAQSEDQARKMFKQMMDGIDYLHKNHIAHRDLKCENLLLDSENTLKVSDFGFARFYDN
                               **  ** *** * *      * *     ****** *****     * *** *

                             181                          210                          240
H. sapiens TSSK1             DDSGRMALSKTFCGSPAYAAPEVLQGIPYQPKVYDIWSLGVILYIMVCGSMPYDDSNIKK
H. sapiens TSSK2             DSNGRIILSKTFCGSAAYAAPEVLQSIPYQPKVYDIWSLGVILYIMVCGSMPYDDSDIRK
H. discus hannai TSSK1-like  G-----VTSKTFCGSAAYAAPEVLQGIPYHVPLHDIWAMGIILYIMICASMPYDDSNLKR
                                     ******* ********* ***     ***  * ***** * *******

                             241                          270                          300
H. sapiens TSSK1             MLRIQKEHRVNFPRSKHLTGECKDLIYHMLQPDVNRRLHIDEILSHCWMQPKARGSPSVA
H. sapiens TSSK2             MLRIQKEHRVDFPRSKNLTCECKDLIYRMLQPDVSQRLHIDEILSHSWLQPPKPKATSSA
H. discus hannai TSSK1-like  MVRDQTEKKVSFSKSKKISVDCKDLVHRILEVNVKKRASIAMMSEHPWIRGASGSASTSV
                             * * * *  * *  **     ****    *   *  *  *     * *

                             301                          330                          360
H. sapiens TSSK1             INKEGESSRGTEPLWTPEPGSDKKSATKLEPEGEAQPQAQPETKPEGTAMQMSRQSEILG
H. sapiens TSSK2             SFK-----REGEGKYRAECKLDTKTDLRPDHRPDHKLGAKTQHRLLVVPENENRMEDRLA
H. discus hannai TSSK1-like  NQP-------------YPIQPVTSEDKRKDKVSEDKARATFLTDLHLRPEERDGKKKSKD
                                                                   *

                             361                      385
H. sapiens TSSK1             FPSKPSTMETEEGPPQQPPETRAQ 367 aa (AAK27734.1)
H. sapiens TSSK2             ETSRAKDHHISGAEVGKAST---- 358 aa (AAH37781.1)
H. discus hannai TSSK1-like  KVGKSETPDMTLQPEAKA------ 359 aa (AWV50517.1)
```

- Total aligned positions including gaps: **385** positions
- No. of residues conserved in all the three proteins: **134** residues (*)
- No. of *H. discus hannai* TSSK1 residues conserved with human TSSK1 but not with human TSSK2: **20** residues
- No. of *H. discus hannai* TSSK1 residues conserved with human TSSK2 but not with human TSSK1: **10** residues

Potential ATP binding sites

Potential polypeptide substrate binding sites

Activation loop

Catalytic domain
