# Supplementary material for: Characterization of testis-specific serine/threonine kinase 1-like (TSSK1-like) gene and expression patterns in diploid and triploid Pacific abalone (Haliotis discus hannai; Gastropoda; Mollusca) males
Source: PLoS One. 2019 Dec 11;14(12):e0226022. doi: 10.1371/journal.pone.0226022 (PMC6905558; doi:10.1371/journal.pone.0226022)
Supplement: S5 Fig — Bootstrap scores are estimated based on 1000 replicates. (PDF) [file pone.0226022.s006.pdf]

S5 Fig

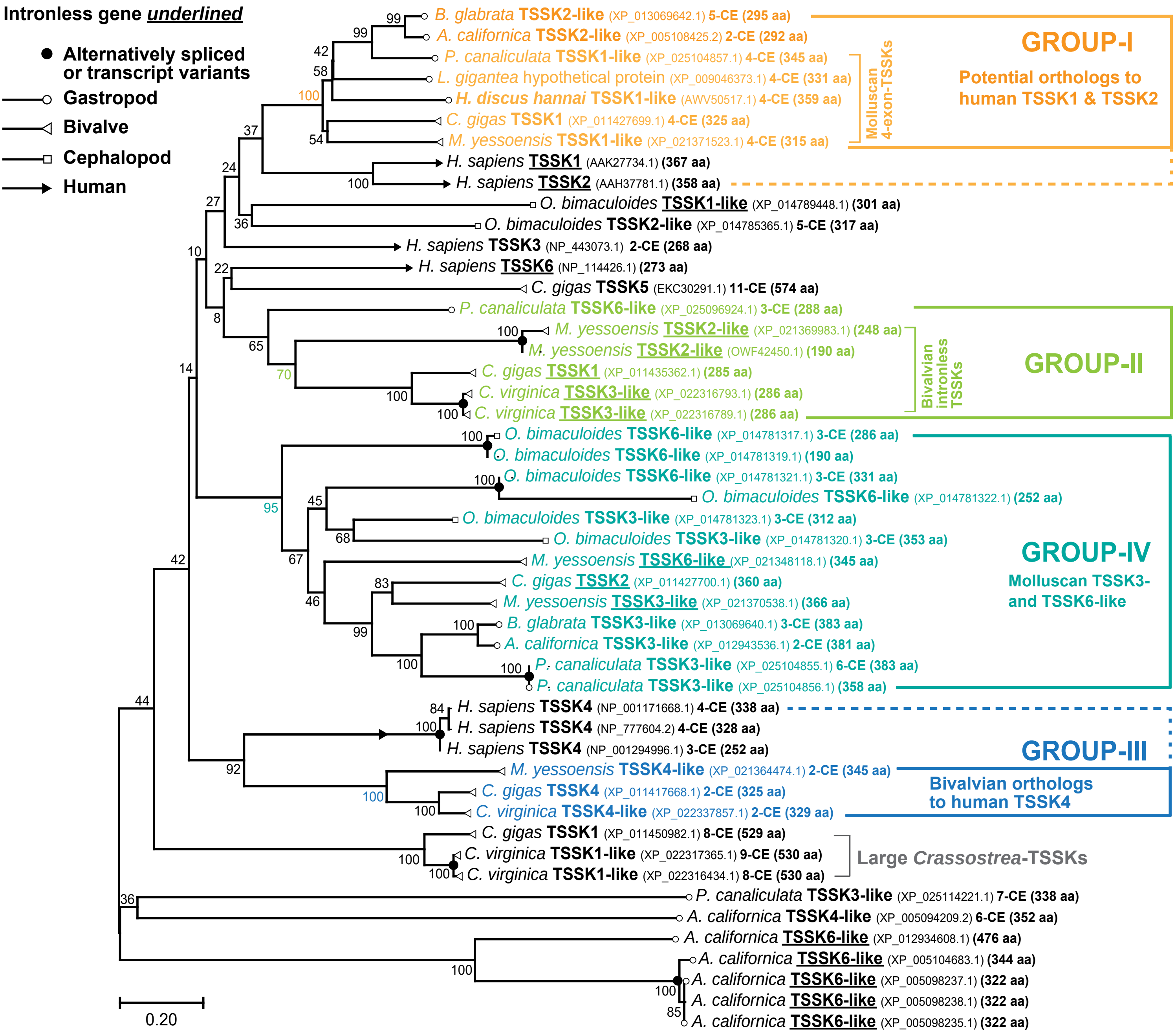

Intronless gene underlined
Alternatively spliced or transcript variants
Gastropod
Bivalve
Cephalopod
Human
B. glabrata TSSK2-like (XP_013069642.1) 5-CE (295 aa)
A. californica TSSK2-like (XP_005108425.2) 2-CE (292 aa)
P. canaliculata TSSK1-like (XP_025104857.1) 4-CE (345 aa)
L. gigantea hypothetical protein (XP_009046373.1) 4-CE (331 aa)
H. discus hannai TSSK1-like (AWV50517.1) 4-CE (359 aa)
C. gigas TSSK1 (XP_011427699.1) 4-CE (325 aa)
M. yessoensis TSSK1-like (XP_021371523.1) 4-CE (315 aa)
H. sapiens TSSK1 (AAK27734.1) (367 aa)
H. sapiens TSSK2 (AAH37781.1) (358 aa)
O. bimaculoides TSSK1-like (XP_014789448.1) (301 aa)
O. bimaculoides TSSK2-like (XP_014785365.1) 5-CE (317 aa)
H. sapiens TSSK3 (NP_443073.1) 2-CE (268 aa)
H. sapiens TSSK6 (NP_114426.1) (273 aa)
C. gigas TSSK5 (EKC30291.1) 11-CE (574 aa)
P. canaliculata TSSK6-like (XP_025096924.1) 3-CE (288 aa)
M. yessoensis TSSK2-like (XP_021369983.1) (248 aa)
M. yessoensis TSSK2-like (OWF42450.1) (190 aa)
C. gigas TSSK1 (XP_011435362.1) (285 aa)
C. virginica TSSK3-like (XP_022316793.1) (286 aa)
C. virginica TSSK3-like (XP_022316789.1) (286 aa)
O. bimaculoides TSSK6-like (XP_014781317.1) 3-CE (286 aa)
O. bimaculoides TSSK6-like (XP_014781319.1) (190 aa)
O. bimaculoides TSSK6-like (XP_014781321.1) 3-CE (331 aa)
O. bimaculoides TSSK6-like (XP_014781322.1) (252 aa)
O. bimaculoides TSSK3-like (XP_014781323.1) 3-CE (312 aa)
O. bimaculoides TSSK3-like (XP_014781320.1) 3-CE (353 aa)
M. yessoensis TSSK6-like (XP_021348118.1) (345 aa)
C. gigas TSSK2 (XP_011427700.1) (360 aa)
M. yessoensis TSSK3-like (XP_021370538.1) (366 aa)
B. glabrata TSSK3-like (XP_013069640.1) 3-CE (383 aa)
A. californica TSSK3-like (XP_012943536.1) 2-CE (381 aa)
P. canaliculata TSSK3-like (XP_025104855.1) 6-CE (383 aa)
P. canaliculata TSSK3-like (XP_025104856.1) (358 aa)
H. sapiens TSSK4 (NP_001171668.1) 4-CE (338 aa)
H. sapiens TSSK4 (NP_777604.2) 4-CE (328 aa)
H. sapiens TSSK4 (NP_001294996.1) 3-CE (252 aa)
M. yessoensis TSSK4-like (XP_021364474.1) 2-CE (345 aa)
C. gigas TSSK4 (XP_011417668.1) 2-CE (325 aa)
C. virginica TSSK4-like (XP_022337857.1) 2-CE (329 aa)
C. gigas TSSK1 (XP_011450982.1) 8-CE (529 aa)
C. virginica TSSK1-like (XP_022317365.1) 9-CE (530 aa)
C. virginica TSSK1-like (XP_022316434.1) 8-CE (530 aa)
P. canaliculata TSSK3-like (XP_025114221.1) 7-CE (338 aa)
A. californica TSSK4-like (XP_005094209.2) 6-CE (352 aa)
A. californica TSSK6-like (XP_012934608.1) (476 aa)
A. californica TSSK6-like (XP_005104683.1) (344 aa)
A. californica TSSK6-like (XP_005098237.1) (322 aa)
A. californica TSSK6-like (XP_005098238.1) (322 aa)
A. californica TSSK6-like (XP_005098235.1) (322 aa)
GROUP-I
Potential orthologs to human TSSK1 & TSSK2
Molluscan 4-exon-TSSKs
GROUP-II
Bivalvian intronless TSSKs
GROUP-IV
Molluscan TSSK3- and TSSK6-like
GROUP-III
Bivalvian orthologs to human TSSK4
Large Crassostrea-TSSKs
0.20
