# Supplementary material for: Characterization of testis-specific serine/threonine kinase 1-like (TSSK1-like) gene and expression patterns in diploid and triploid Pacific abalone (Haliotis discus hannai; Gastropoda; Mollusca) males
Source: PLoS One. 2019 Dec 11;14(12):e0226022. doi: 10.1371/journal.pone.0226022 (PMC6905558; doi:10.1371/journal.pone.0226022)

**S6 Fig**

# Information on sequences used in alignments

| Code in alignment | Group | Species | GenBank # |
|---|---|---|---|
| CD103Ho | Mammalia | *Homo sapiens* | NP_114417.1 |
| CD082 | Mammalia | *Delphinapterus leucas* | XP_022407189.1 |
| CD083 | Mammalia | *Orcinus orca* | XP_004276055.1 |
| CD094 | Mammalia | *Otolemur garnettii* | XP_003801742.1 |
| CD086 | Mammalia | *Bos taurus* | NP_001077179.1 |
| CD081 | Mammalia | *Balaenoptera acutorostrata* | XP_007196324.1 |
| CD089 | Mammalia | *Miniopterus natalensis* | XP_016053763.1 |
| CD101 | Mammalia | *Elephantulus edwardii* | XP_006902591.1 |
| CD092 | Mammalia | *Canis lupus familiaris* | XP_013967648.1 |
| CD085 | Mammalia | *Sus scrofa* | NP_001274344.1 |
| CD099 | Mammalia | *Loxodonta africana* | XP_023397211.1 |
| CD102 | Mammalia | *Trichechus manatus* | XP_004390166.1 |
| CD087 | Mammalia | *Camelus dromedarius* | XP_010985656.1 |
| CD088 | Mammalia | *Camelus ferus* | XP_014415551.1 |
| CD095 | Mammalia | *Mus musculus* | NP_033461.2 |
| CD097 | Mammalia | *Ictidomys tridecemlineatus* | XP_005334578.1 |
| CD096 | Mammalia | *Rattus norvegicus* | NP_001011900.1 |
| CD090 | Mammalia | *Pteropus vampyrus* | XP_011354208.1 |
| CD084 | Mammalia | *Lipotes vexillifer* | XP_007460074.1 |
| CD098 | Mammalia | *Orycteropus afer afer* | XP_007953634.1 |
| CD093 | Mammalia | *Galeopterus variegatus* | XP_008590054.1 |
| CD091 | Mammalia | *Sorex araneus* | XP_004607653.1 |
| CD104 | Mammalia | *Pan troglodytes* | NP_001315253.1 |
| CD105 | Mammalia | *Macaca mulatta* | NP_001180397.1 |
| CD108 | Sauropsida | *Nothoprocta perdicaria* | XP_025893540.1 |
| CD109 | Sauropsida | *Dromaius novaehollandiae* | XP_025960119.1 |
| CD111 | Sauropsida | *Anolis carolinensis* | XP_003225155.3 |
| CD112 | Sauropsida | *Gekko japonicus* | XP_015283994.1 |
| CD110 | Sauropsida | *Notechis scutatus* | XP_026542456.1 |
| CD117 | Sauropsida | *Crocodylus porosus* | XP_019394967.1 |
| CD114 | Sauropsida | *Chelonia mydas* | XP_007055073.1 |
| CD115 | Sauropsida | *Chrysemys picta* | XP_005281306.1 |
| CD050 | | *Rhincodon typus* | XP_020377052.1 |
| CD071 | Actinopterygii | *Oncorhynchus tshawytscha* | XP_024236489.1 |
| CD079 | Actinopterygii | *Oncorhynchus mykiss* | XP_021458380.1 |
| CD077 | Actinopterygii | *Oncorhynchus kisutch* | XP_020313211.1 |
| CD073 | Actinopterygii | *Salvelinus alpinus* | XP_023841938.1 |
| CD057 | Actinopterygii | *Salmo salar* | XP_013986042.1 |
| CD051 | Actinopterygii | *Acipenser ruthenus* | RXM91403.1 |
| CD076 | Actinopterygii | *Scleropages formosus* | XP_018605351.1 |
| CD058 | Actinopterygii | *Clupea harengus* | XP_012697071.1 |

| Code in alignment | Group | Species | GenBank # |
|---|---|---|---|
| CD053 | Actinopterygii | *Astatotilapia calliptera* | XP_026030089.1 |
| CD066 | Actinopterygii | *Maylandia zebra* | XP_004565771.1 |
| CD065 | Actinopterygii | *Pundamilia nyererei* | XP_005726447.1 |
| CD063 | Actinopterygii | *Haplochromis burtoni* | XP_005924056.1 |
| CD062 | Actinopterygii | *Neolamprologus brichardi* | XP_006802376.1 |
| CD067 | Actinopterygii | *Oreochromis niloticus* | XP_003437559.1 |
| CD060 | Actinopterygii | *Poecilia reticulata* | XP_008431463.1 |
| CD064 | Actinopterygii | *Xiphophorus maculatus* | XP_014325498.1 |
| CD061 | Actinopterygii | *Stegastes partitus* | XP_008292276.1 |
| CD074 | Actinopterygii | *Kryptolebias marmoratus* | XP_017275054.1 |
| CD068 | Actinopterygii | *Lates calcarifer* | XP_018527549.1 |
| CD078 | Actinopterygii | *Labrus bergylta* | XP_020510131.1 |
| CD069 | Actinopterygii | *Tachysurus fulvidraco* | XP_026996966.1 |
| CD070 | Actinopterygii | *Pangasianodon hypophthalmus* | XP_026798493.1 |
| CD118 | | *Ciona intestinalis* **(tunicate)** | XP_018670884.1 |
| CD044LG | Mollusca | *Lottia gigantea* | XP_009046373.1 |
| **CD045HD** | Mollusca | ***Haliotis discus hannai*** | **AWV50517.1** |
| CD043PC1 | Mollusca | *Pomacea canaliculata* | XP_025104857.1 |
| CD046CG1 | Mollusca | *Crassostrea gigas* | XP_011427699.1 |
| CD047MY1 | Mollusca | *Mizuhopecten yessoensis* | XP_021371523.1 |
| CD039 | Lingulida | *Lingula anatina* **(Brachiophoda)** | XP_013421518.1 |
| CD121 | Starfish | *Acanthaster planci* **(Echinodermata)** | XP_022089018.1 |
| CD124 | Sea urchin | *Strongylocentrotus purpuratus* | XP_787834.2 |
| CD001 | Sea anemones | *Exaiptasia pallida* **(Cnidaria)** | XP_020908236.1 |
| CD036 | Dog roundworm | *Toxocara canis* **(Nematoda)** | KHN81710.1 |
| CD020 | Arthropoda | Nicrophorus vespilloides | XP_017781794.1 |
| CD023 | Arthropoda | Halyomorpha halys | XP_014284685.1 |
| CD010 | Arthropoda | Parasteatoda tepidariorum | XP_015917553.1 |
| CD015 | Arthropoda | Centruroides sculpturatus | XP_023214764.1 |
| CD011 | Arthropoda | Tetranychus urticae | XP_015792291.1 |
| CD013 | Arthropoda | Dermatophagoides pteronyssinus | XP_027194462.1 |
| CD017 | Arthropoda | Limulus polyphemus | XP_013777890.1 |
| CD024 | Arthropoda | Polistes dominula | XP_015179999.1 |
| CD028 | Arthropoda | Atta cephalotes | XP_012054624.1 |
| CD030 | Arthropoda | Bactrocera dorsalis | XP_011211029.1 |
| CD031 | Arthropoda | Bactrocera latifrons | XP_018803871.1 |
| CD032 | Arthropoda | Ceratitis capitata | XP_004536887.1 |
| CD029 | Arthropoda | Drosophila erecta | XP_001979628.1 |
| CD033 | Arthropoda | Aedes aegypti | XP_021705955.1 |
| CD034 | Arthropoda | Culex quinquefasciatus | XP_001843676.1 |
| CD026 | Arthropoda | Dufourea novaeangliae | XP_015430419.1 |

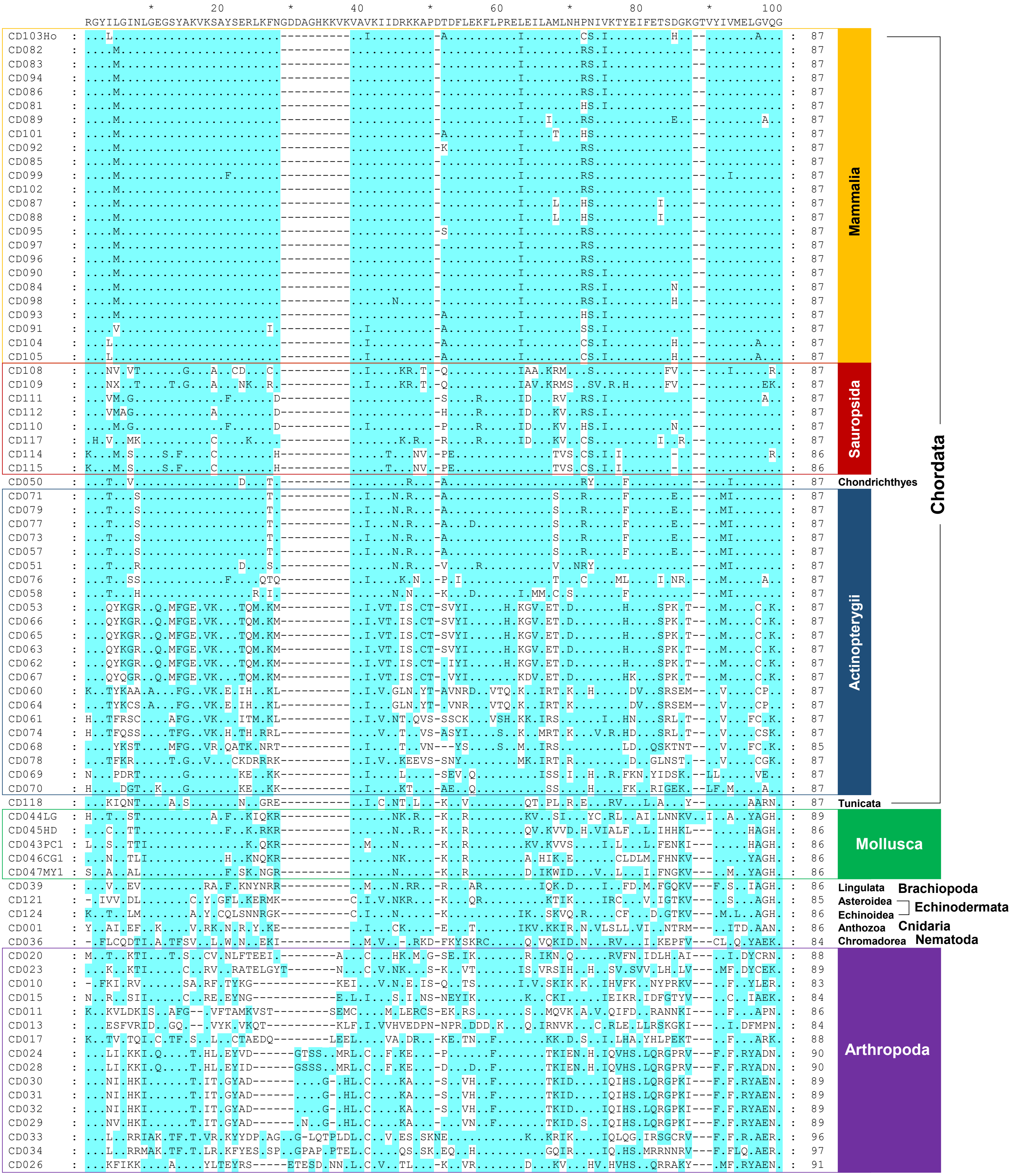
RGYILGINLGEGSYAKVKSAYSERLKFNGDDAGHKKVKVAVKIIDRKKAPDTDFLEKFLPRELEILAMLNHPNIVKTYEIFETSDGKGTVYIVMELGVQG
Mammalia
Sauropsida
Chondrichthyes
Actinopterygii
Tunicata
Chordata
Mollusca
Lingulata
Brachiopoda
Asteroidea
Echinoidea
Echinodermata
Anthozoa
Cnidaria
Chromadorea
Nematoda
Arthropoda

Alignment continued

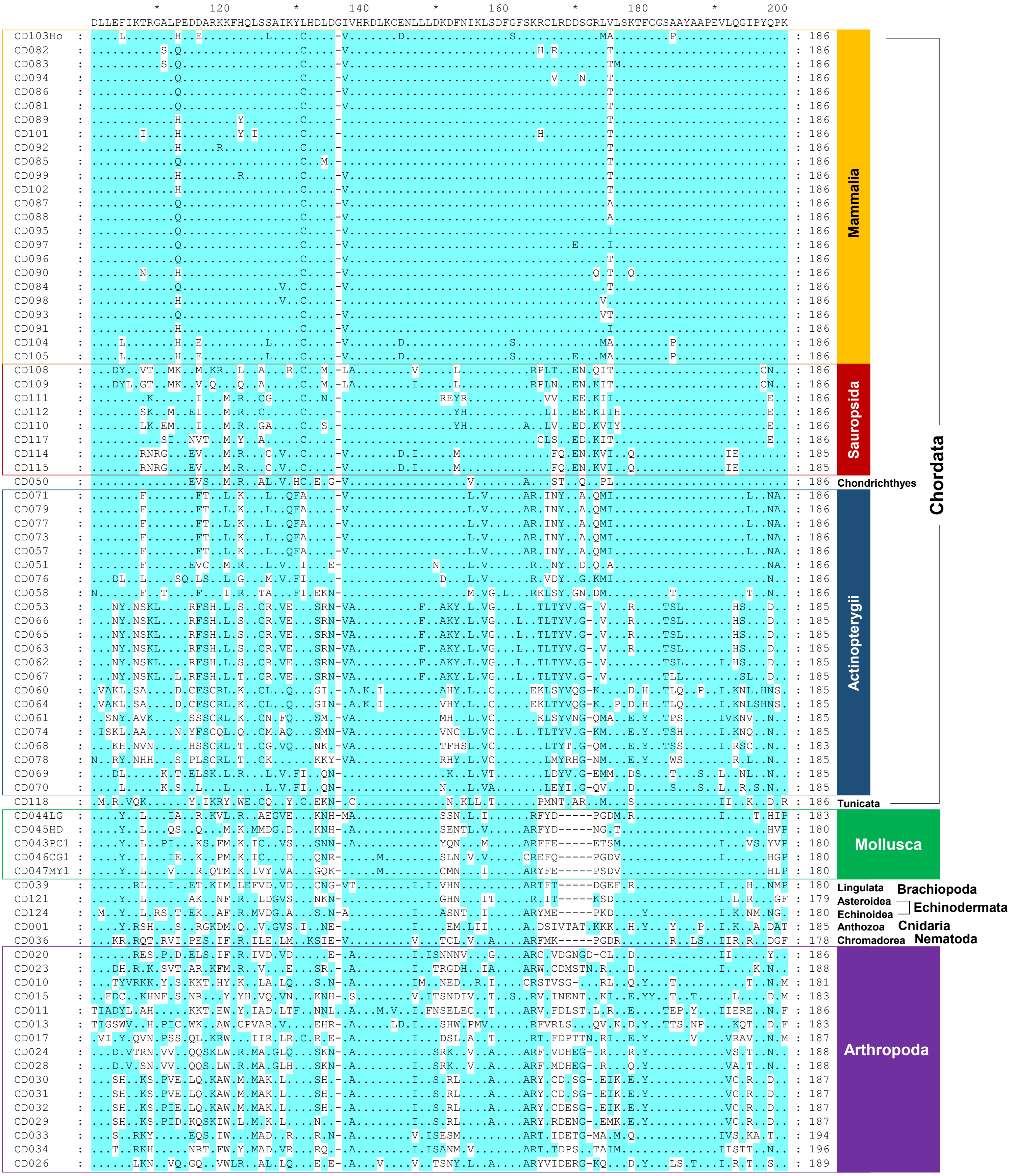

# Alignment continued

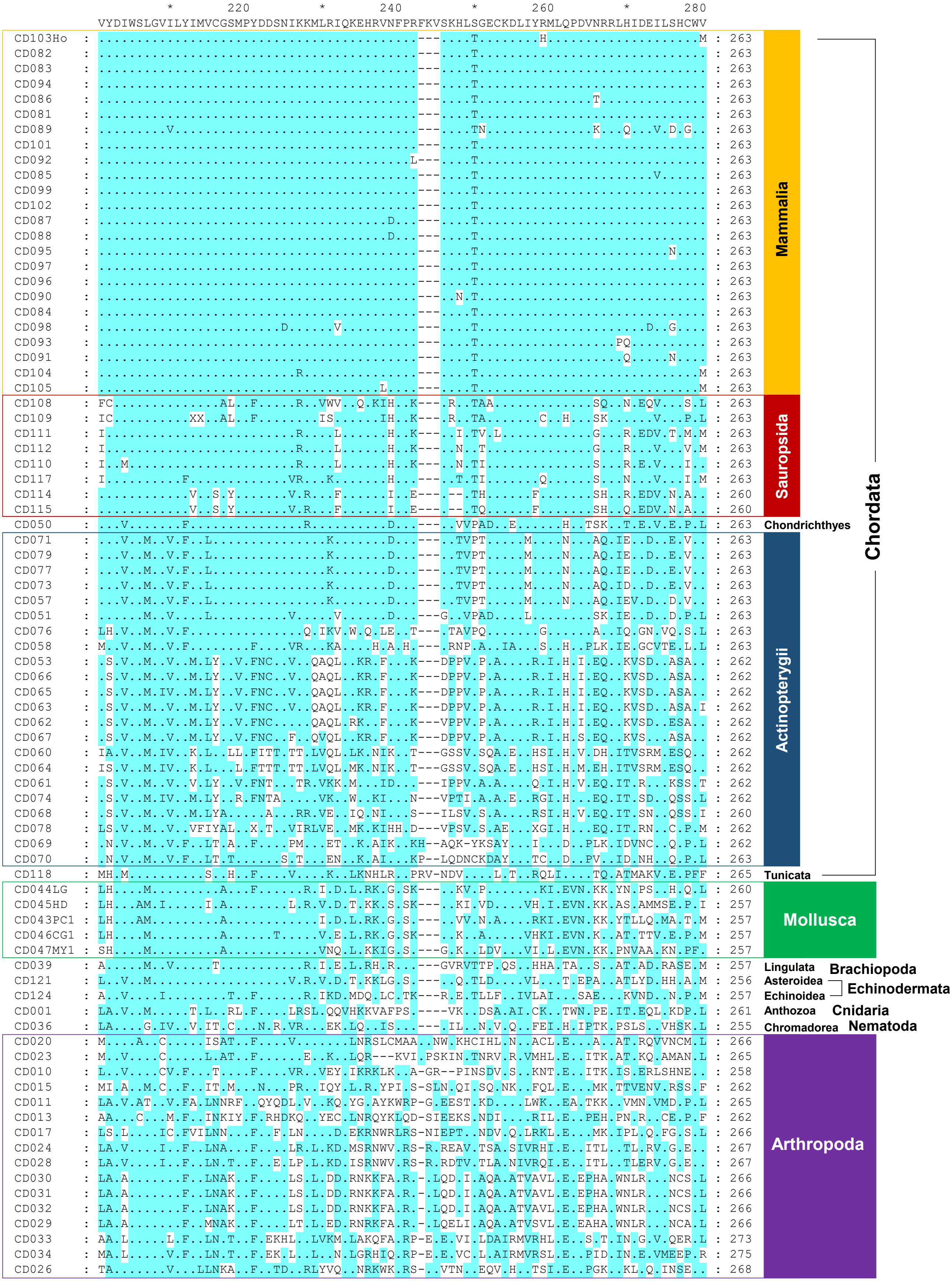

Supplement: S6 Fig — In total, 82 taxa were sampled to examine structural diversity and molecular phylogenetic analyses in the metazoan lineage. (PDF) [file pone.0226022.s007.pdf]
