# Supplementary material for: Characterization of testis-specific serine/threonine kinase 1-like (TSSK1-like) gene and expression patterns in diploid and triploid Pacific abalone (Haliotis discus hannai; Gastropoda; Mollusca) males
Source: PLoS One. 2019 Dec 11;14(12):e0226022. doi: 10.1371/journal.pone.0226022 (PMC6905558; doi:10.1371/journal.pone.0226022)
Supplement: S1 Table — (PDF) [file pone.0226022.s016.pdf]

S1 Table. Oligonucleotide primers used in this study

Primer name	Sequence (5´-3´)	Purpose
HHTSSK-3RACE-1	TATGTGCCTCAATGCCGTACGA	3´RACE
HHTSSK-3RACE-2	GCATGGTCAGAGACCAAACTGA	
HHTSSK-5RACE-1	TCGAAGGTGAAGGTCAGTCAGA	5´RACE
HHTSSK-5RACE-2	TTGTCTTCGCTCACTTTGTCCT	
HHTSSK1-cDNA FW1	ATTCTACGCCGCGCGATGGT	RT-PCR isolation of full-length ORF
HHTSSK1-cDNA RV1	CCTTCGAAACGAGTGAATAACG	
HHTSSK1-CDS FW1	CCCTGTCTGTAGAGATTGTT	RT-PCR isolation of coding region for testing sequence polymorphism
HHTSSK1-CDS RV1	ACGCGTTAACAGCGTTAGAG	
HHTSSK1-gF1	TCGCCCCTGTCTGTAGAGATT	PCR isolation & cloning of genomic gene
HHTSSK1-gR1	GGGCTATGTGATTCTTGTGG	
HHTSSK1-gF2	ACGGTGACCTCTTGGAGTAT	
HHTSSK1-gR2	TAGAGGATGATCCCCATTGC	
HHTSSK1-gF3	CAAGTGCGAGAACCTTCTGC	
HHTSSK1-gR3	GTCATATCCGGCGTCTCACT	
HHTSSK1-gF4	ATATGTGCCTCAATGCCGTAC	
HHTSSK1-gR4	CGAGTGCATAACGATTAGTACAG	
HHTSSK1g-Seq1F	TTCTACGCCGCGCGATGGTC	PCR isolation of overlapping gDNA fragment for sequence validation of genomic gene
HHTSSK1g-Seq1R	CCGACATGGGTACAATGTGT	
HHTSSK1g-Seq2F	TGGCCGACATTTCATGGAAG	
HHTSSK1g-Seq2R	GAATGGTCGAAGGCAGCAAT	
HHTSSK1g-Seq3F	TGTGTCATGGTTTCTCGGCA	
HHTSSK1g-Seq3R	GCATGTCATCGATCCCAATG	
HHTSSK1g-Seq4F	TCCAGCTATATGACGGCGGT	
HHTSSK1g-Seq4R	AACGCGTTAACAGCGTTAGAG	
HHTSSK1g-Seq5F	GTGTGAAGCCCATTTCTGGT	
HHTSSK1g-Seq5R	TAGTAATAACCTTCGAAACGAGTG	
INT1-1F	GACCGGTAAGAACAGATCGC	Cloning analysis and PCR typing of intronic length polymorphisms
INT1-1R	GTTGACTCCATCAGGCATTG	
INT2-1F	TCGTTCTGAAGAGTTGCGTC	
INT2-1R	GGTAAGTGTATCGGCGTCA	
INT2-2F	CACCTTCCATGTGTGGTTTG	
INT2-2R	CTTTGCAGAGTTGCGTCCCT	
INT2-3F	AACTCTGCAAAGCGAATCGC	
INT2-3R	TCACGTGTCCCTCATACCAT	
HH-TSSK1 q1F	AGGTGAACGTCAAGAAACGC	End point RT-PCR and RT-qPCR assays of TSSK1-like transcripts
HH-TSSK1 q1R	ATTCACTCAACCGCACTCAC	
HH-RPL5 q1F	AGATGAGGATGGCAAACCAG	An internal control for end point RT-PCR and RT-qPCR assays
HH-RPL5 q1R	TCGCTGCTCTCAGAGTCAAA	
