# Supplementary material for: Characterization of testis-specific serine/threonine kinase 1-like (TSSK1-like) gene and expression patterns in diploid and triploid Pacific abalone (Haliotis discus hannai; Gastropoda; Mollusca) males
Source: PLoS One. 2019 Dec 11;14(12):e0226022. doi: 10.1371/journal.pone.0226022 (PMC6905558; doi:10.1371/journal.pone.0226022)
Supplement: S2 Table — (PDF) [file pone.0226022.s017.pdf]

S2 Table. Information on TSSK1 (TSSK1-like) sequences used in the molecular phylogenetic analyses in the metazoan lineage

Species (common name)	Taxonomic position	GenBank name	GenBank accession code	No. AA residues	No. of coding exon	Mw	pI	Identity to abalone TSSK1-like
**Cnidaria**								
Exaiptasia pallida (sea anemone; sponge)	Anthozoa>Actiniaria	TSSK1	XP_020908236.1	310	2	34791.37	9.18	55.5%
**Protostomia**								
Parasteatoda tepidariorum (house spider)	Ecdysozoa>Arthropoda>Cheliceata>Arachnida	TSSK1-like	XP_015917553.1	318	5	37016.93	9.51	53.1%
Tetranychus urticae (spider mite)	Ecdysozoa>Arthropoda>Cheliceata>Arachnida	TSSK1-like	XP_015792291.1	295	1	34357.93	9.02	53.1%
Dermatophagoides pteronyssinus (dust mite)	Ecdysozoa>Arthropoda>Cheliceata>Arachnida	TSSK1-like	XP_027194462.1	295	2	34675.00	8.57	51.3%
Centruroides sculpturatus (bark scorpion)	Ecdysozoa>Arthropoda>Cheliceata>Arachnida	TSSK1-like	XP_023214764.1	286	4	33230.62	9.12	54.8%
Limulus polyphemus (Atlantic horseshoe crab)	Ecdysozoa>Arthropoda>Cheliceata>Merostomata	TSSK1-like	XP_013777890.1	323	1	37181.97	9.08	50.1%
Nicrophorus vespilloides (beetle)	Ecdysozoa>Arthropoda>Hexapoda>Insecta	TSSK1-like	XP_017781794.1	302	6	34770.77	8.54	56.4%
Halyomorpha halys (stink bug)	Ecdysozoa>Arthropoda>Hexapoda>Insecta	TSSK1	XP_014284685.1	302	2	34462.45	9.54	56.4%
Polistes dominula (paper wasp)	Ecdysozoa>Arthropoda>Hexapoda>Insecta	TSSK1	XP_015179999.1	320	3	36671.66	9.84	54.5%
Dufourea novaeangliae (bee)	Ecdysozoa>Arthropoda>Hexapoda>Insecta	TSSK1-like	XP_015430419.1	355	4	41004.03	9.46	55.0%
Atta cephalotes (ant)	Ecdysozoa>Arthropoda>Hexapoda>Insecta	TSSK1	XP_012054624.1	360	4	40495.72	9.72	53.8%
Drosophila erecta (fly)	Ecdysozoa>Arthropoda>Hexapoda>Insecta	TSSK1	XP_001979628.1	302	2	34218.28	9.09	54.8%
Bactrocera dorsalis (fly)	Ecdysozoa>Arthropoda>Hexapoda>Insecta	TSSK1	XP_011211029.1	299	1	33771.81	8.57	55.0%
Bactrocera latifrons (fly)	Ecdysozoa>Arthropoda>Hexapoda>Insecta	TSSK1	XP_018803871.1	299	1	33771.81	8.57	55.0%
Ceratitis capitata (fly)	Ecdysozoa>Arthropoda>Hexapoda>Insecta	TSSK1	XP_004536887.1	299	1	33785.84	8.57	55.0%
Aedes aegypti (mosquito)	Ecdysozoa>Arthropoda>Hexapoda>Insecta	TSSK1-like	XP_021705955.1	323	1	36674.94	9.57	50.6%
Culex quinquefasciatus (mosquito)	Ecdysozoa>Arthropoda>Hexapoda>Insecta	TSSK1	XP_001843676.1	351	1	39971.09	9.66	44.6%
Toxocara canis (dog roundworm)	Ecdysozoa>Nematoda	TSSK1	KHN81710.1	312	7	36121.15	9.54	56.1%
Lingula anatina (lingulida)	Lophotrochozoa>Brachiopoda	TSSK1	XP_013421518.1	351	4	39759.45	9.03	63.1%
Pomacea canaliculata (apple snail)	Lophotrochozoa>Mollusca>Gastropoda	TSSK1-like	XP_025104857.1	345	4	39291.31	8.58	71.2%
Lottia gigantea (owl limpet)	Lophotrochozoa>Mollusca>Gastropoda	Hypothetical	XP_009046373.1	331	4	37797.29	8.99	71.0%
Haliotis discus hannai (abalone)	Lophotrochozoa>Mollusca>Gastropoda	TSSK1 (present study)	AWV50517.1	359	4	40999.50	9.29	100%
Crassostrea gigas (oyster)	Lophotrochozoa>Mollusca>Bivalvia	TSSK1	XP_011427699.1	325	4	36891.04	8.89	70.8%
Mizuhopecten yessoensis (scallop)	Lophotrochozoa>Mollusca>Bivalvia	TSSK1-like	XP_021371523.1	315	4	35614.36	9.14	69.6%
**Deuterostomia**								
Acanthaster planci (starfish)	Echinodermata>Asterozoa	TSSK1-like	XP_022089018.1	358	2	40565.03	8.92	64.0%
Strongylocentrotus purpuratus (sea urchin)	Echinodermata>Echinozoa>Echinoidea	TSSK1-like	XP_787834.2	445	5	49899.13	9.87	47.3%
Ciona intestinalis (tunicate)	Chordata>Tunicata>Ascidiacea	TSSK1-like	XP_018670884.1	407	7	46348.96	9.14	48.3%
Rhincodon typus (whale shark)	Chordata>Craniata>Vertebrata> Chondrichthyes	TSSK1-like	XP_020377052.1	489	1	56410.99	9.04	37.6%
**Teleostomi> Euteleostomi>Actinopterygii**								
Acipenser ruthenus (sturgeon)	Chondrostei	TSSK1	RXM91403.1	363	1	41520.61	8.54	60.6%
Scleropages formosus (Asian arowana)	Osteoglossomorpha>Osteoglossiformes	TSSK1-like	XP_018605351.1	312	1	35183.76	8.64	61.9%
Clupea harengus (Atlantic herring)	Otomorpha>Clupei> Clupeiformes	TSSK1-like	XP_012697071.1	335	1	37767.72	8.89	60.1%
Tachysurus fulvidraco (yellowhead catfish)	Otomorpha>Ostariophysi>Siluriformes	TSSK1-like	XP_026996966.1	303	1	34951.12	9.33	56.4%
Pangasianodon hypophthalmus (shark catfish)	Otomorpha>Ostariophysi>Siluriformes	TSSK1-like	XP_026798493.1	392	1	44494.60	9.08	52.7%
Salmo salar (Atlantic salmon)	Euteleosteomorpha>Protacanthopterygii>Salmoniformes	TSSK1-like	XP_013986042.1	360	1	40649.65	8.33	60.6%
Oncorhynchus tshawytscha (Chinook salmon)	Euteleosteomorpha>Protacanthopterygii>Salmoniformes	TSSK1-like	XP_024236489.1	391	1	44052.63	8.02	58.5%
Salvelinus alpinus (arctic char)	Euteleosteomorpha>Protacanthopterygii>Salmoniformes	TSSK1-like	XP_023841938.1	360	1	40584.62	8.65	61.0%

Oncorhynchus kisutch (Coho salmon)	Euteleosteomorpha>Protacanthopterygii>Salmoniformes	TSSK1-like	XP_020313211.1	360	1	40694.74	8.51	60.8%	
Oncorhynchus mykiss (rainbow trout)	Euteleosteomorpha>Protacanthopterygii>Salmoniformes	TSSK1-like	XP_021458380.1	360	1	40708.76	8.51	61.0%	
Astatotilapia calliptera (Eastern happy)	Euteleosteomorpha>Neoteleostei>Acanthomorpha>Cichliformes	TSSK1-like	XP_026030089.1	308	2	34863.44	9.21	54.1%	
Neolamprologus brichardi (lretail cichlid)	Euteleosteomorpha>Neoteleostei>Acanthomorpha>Cichliformes	TSSK1-like	XP_006802376.1	308	2	34844.39	8.90	54.3%	
Haplochromis burtoni (astaotilapia burtoni)	Euteleosteomorpha>Neoteleostei>Acanthomorpha>Cichliformes	TSSK1-like	XP_005924056.1	308	2	34907.49	9.21	54.3%	
Pundamilia nyererei	Euteleosteomorpha>Neoteleostei>Acanthomorpha>Cichliformes	TSSK1-like	XP_005726447.1	308	2	34877.46	9.21	54.3%	
Maylandia zebra (zebra mbuna)	Euteleosteomorpha>Neoteleostei>Acanthomorpha>Cichliformes	TSSK1-like	XP_004565771.1	308	2	34764.30	9.14	54.1%	
Oreochromis niloticus (Nile tilapia)	Euteleosteomorpha>Neoteleostei>Acanthomorpha>Cichliformes	TSSK1	XP_003437559.1	308	2	34938.54	9.06	54.5%	
Poecilia reticulata (guppy)	Euteleosteomorpha>Neoteleostei>Acanthomorpha>Cyprinodontiformes	TSSK1-like	XP_008431463.1	315	2	35356.18	8.03	52.5%	
Xiphophorus maculatus (Southern platyfish)	Euteleosteomorpha>Neoteleostei>Acanthomorpha>Cyprinodontiformes	TSSK1-like	XP_014325498.1	306	2	34616.23	8.27	53.6%	
Kryptolebias marmoratus (mangrove rivulus)	Euteleosteomorpha>Neoteleostei>Acanthomorpha>Cyprinodontiformes	TSSK1-like	XP_017275054.1	322	2	36129.70	8.90	54.5%	
Stegastes partitus (bicolor damselfish)	Euteleosteomorpha>Neoteleostei>Acanthomorpha>Ovalentaria incertae	TSSK1-like	XP_008292276.1	320	2	36200.77	9.10	53.6%	
Labrus bergylta (Ballan wrasse)	Euteleosteomorpha>Neoteleostei>Acanthomorpha>Labriformes	TSSK1-like	XP_020510131.1	323	2	37326.39	8.45	56.2%	
Lates calcarifer (barramundi)	Euteleosteomorpha>Neoteleostei>Acanthomorpha>Carangaria incertae	TSSK1-like	XP_018527549.1	329	2	37333.69	6.79	54.7%	
**Teleostomi>Euteleostomi>Sarcopterygii>Tetrapoda>Amniota**									
Balaenoptera acutorostrata (minke whale)	Mammalia>Theria>Eutheria>Boreoeutheria>Laurasiatheria	TSSK1	XP_007196324.1	367	1	41719.83	7.61	59.4%	
Delphinapterus leucas (beluga whale)	Mammalia>Theria>Eutheria>Boreoeutheria>Laurasiatheria	TSSK1	XP_022407189.1	368	1	41934.10	8.49	58.9%	
Orcinus orca (killer whale)	Mammalia>Theria>Eutheria>Boreoeutheria>Laurasiatheria	TSSK1	XP_004276055.1	368	1	41937.20	8.49	58.9%	
Lipotes vexillifer (river dolphin)	Mammalia>Theria>Eutheria>Boreoeutheria>Laurasiatheria	TSSK1-like	XP_007460074.1	368	1	41781.04	8.63	58.7%	
Sus scrofa (pig)	Mammalia>Theria>Eutheria>Boreoeutheria>Laurasiatheria	TSSK1	NP_001274344.1	364	1	41341.28	6.53	59.2%	
Bos taurus (cattle)	Mammalia>Theria>Eutheria>Boreoeutheria>Laurasiatheria	TSSK1	NP_001077179.1	367	1	41568.62	7.11	59.4%	
Camelus dromedarius (camel)	Mammalia>Theria>Eutheria>Boreoeutheria>Laurasiatheria	TSSK1	XP_010985656.1	414	1	46646.30	6.41	51.1%	
Camelus ferus (camel)	Mammalia>Theria>Eutheria>Boreoeutheria>Laurasiatheria	TSSK1	XP_014415551.1	421	1	47453.98	6.26	49.9%	
Miniopterus natalensis (bat)	Mammalia>Theria>Eutheria>Boreoeutheria>Laurasiatheria	TSSK1-like	XP_016053763.1	371	1	42090.11	7.59	59.2%	
Pteropus vampyrus (flying fox)	Mammalia>Theria>Eutheria>Boreoeutheria>Laurasiatheria	TSSK1	XP_011354208.1	370	1	42031.22	7.11	58.7%	
Sorex araneus (European sherew)	Mammalia>Theria>Eutheria>Boreoeutheria>Laurasiatheria	TSSK1	XP_004607653.1	367	1	41368.29	6.35	59.9%	
Canis lupus familiaris (dog)	Mammalia>Theria>Eutheria>Boreoeutheria>Laurasiatheria	TSSK1	XP_013967648.1	369	1	42021.05	6.25	58.7%	
Galeopterus variegatus (flying lemur)	Mammalia>Theria>Eutheria>Boreoeutheria>Euarchontoglires	TSSK1	XP_008590054.1	362	2	41069.98	6.54	59.4%	
Otolemur garnettii (galago)	Mammalia>Theria>Eutheria>Boreoeutheria>Euarchontoglires	TSSK1	XP_003801742.1	366	2	41735.72	6.58	59.4%	
Mus musculus (mouse)	Mammalia>Theria>Eutheria>Boreoeutheria>Euarchontoglires	TSSK1	NP_033461.2	365	1	41588.51	7.61	59.2%	
Rattus norvegicus (rat)	Mammalia>Theria>Eutheria>Boreoeutheria>Euarchontoglires	TSSK1	NP_001011900.1	365	1	41420.27	7.61	59.2%	
Ictidomys tridecemlineatus (squirrel)	Mammalia>Theria>Eutheria>Boreoeutheria>Euarchontoglires	TSSK1	XP_005334578.1	369	1	42189.29	7.14	58.9%	
Homo sapiens (human)	Mammalia>Theria>Eutheria>Boreoeutheria>Euarchontoglires	TSSK1	NP_114417.1	367	1	41617.93	7.60	58.2%	
Pan troglodytes (chimpanzee)	Mammalia>Theria>Eutheria>Boreoeutheria>Euarchontoglires	TSSK1	NP_001315253.1	367	1	41699.95	7.13	58.3%	
Macaca mulatta (monkey)	Mammalia>Theria>Eutheria>Boreoeutheria>Euarchontoglires	TSSK1	NP_001180397.1	366	1	41582.97	6.58	58.7%	
Orycteropus afer afer (aardvark)	Mammalia>Theria>Eutheria>Boreoeutheria>Afrotheria	TSSK1	XP_007953634.1	366	1	41350.31	7.59	59.9%	
Loxodonta africana (African elephant)	Mammalia>Theria>Eutheria>Boreoeutheria>Afrotheria	TSSK1	XP_023397211.1	366	1	41549.47	7.11	59.2%	
Elephantulus edwardii (cape elephant shrew)	Mammalia>Theria>Eutheria>Boreoeutheria>Afrotheria	TSSK1-like	XP_006902591.1	367	1	41486.41	6.65	59.8%	
Trichechus manatus (Florida manatee)	Mammalia>Theria>Eutheria>Boreoeutheria>Afrotheria	TSSK1	XP_004390166.1	366	1	41651.61	6.62	59.4%	
Nothoprocta perdicaria (Chilean tinamou)	Sauropsida>Sauria>Archelosauria>Dinosauria>Aves>Palaeognathae	TSSK1-like	XP_025893540.1	322	1	36539.27	7.96	59.2%	
Dromaius novaehollandiae (emu)	Sauropsida>Sauria>Archelosauria>Dinosauria>Aves>Palaeognathae	TSSK1-like	XP_025960119.1	361	1	40782.42	6.22	57.6%	
Chelonia mydas (sea turtle)	Sauropsida>Sauria>Archelosauria>Tesudines	TSSK1	XP_007055073.1	281	1	32559.00	8.51	59.1%	
Chrysemys picta (pained turtle)	Sauropsida>Sauria>Archelosauria>Tesudines	TSSK1-like	XP_005281306.1	281	1	32493.88	8.04	59.1%	
Crocodylus porosus (crocodile)	Sauropsida>Sauria>Archelosauria>Crocodylia	TSSK1-like	XP_019394967.1	361	1	41298.67	8.21	59.2%	
Notechis scutatus (tiger snake)	Sauropsida>Sauria>Lepidosauria>Squamata	TSSK1-like	XP_026542456.1	386	1	44035.64	6.90	58.5%	
Anolis carolinensis (anole)	Sauropsida>Sauria>Lepidosauria>Squamata	TSSK1-like	XP_003225155.3	338	1	38690.80	8.08	62.7%	
Gekko japonicus (gecko)	Sauropsida>Sauria>Lepidosauria>Squamata	TSSK1-like	XP_015283994.1	350	1	39870.10	7.59	60.8%	
